# Prognostic Impact of miR-34a in Head and Neck Squamous Cell Carcinoma: A Systematic Review with Meta-Analysis and Trial Sequential Analysis

**DOI:** 10.3390/ijms27114909

**Published:** 2026-05-29

**Authors:** Mario Dioguardi, Stefania Cantore, Ciro Guerra, Diego Sovereto, Giorgia Pia Camerino, Angelo Martella, Raffaele Piccinonno, Antonio Lo Muzio, Mariarosaria Boccellino, Lorenzo Lo Muzio, Andrea Ballini, Alfredo De Rosa

**Affiliations:** 1 Department of Clinical and Experimental Medicine, University of Foggia, Via Rovelli 50, 71122 Foggia, Italy; mario.dioguardi@unifg.it (M.D.); ciro_guerra.556675@unifg.it (C.G.); diego_sovereto.546709@unifg.it (D.S.); giorgia_camerino.612844@unifg.it (G.P.C.); lorenzo.lomuzio@unifg.it (L.L.M.); 2 Consorzio Interuniversitario Nazionale per la Bio-Oncologia (CINBO), 66100 Chieti, Italy; m.boccellino@unilink.it (M.B.); a.ballini@unilink.it (A.B.); a.derosa@unilink.it (A.D.R.); 3 Department of Life Science, Health and Health Professions, Link Campus University, 00165 Rome, Italy; 4 DataLab, Department of Engineering for Innovation, University of Salento, 73100 Lecce, Italy; angelo.martella@unisalento.it; 5 Unità Operativa Semplice Dipartimentale (U.O.S.D.) di Odontoiatria e Stomatologia del Presidio Ospedaliero “V. Fazzi”, 73100 Lecce, Italy; raffaele.piccinonno@asl.lecce.it; 6 School of Dentistry, Saint Camillus International University of Health and Medical Sciences, 00131 Rome, Italy; u.004026@students.unicamillus.org

**Keywords:** HNSCC, LSCC, OSCC, OPSCC, miR-34a, oral cancer, non-coding RNA, microRNA

## Abstract

Dysregulated microRNA (miR) expression has emerged as a potential prognostic tool in head and neck squamous cell carcinoma (HNSCC), but the clinical value of miR-34a remains unclear. This systematic review, meta-analysis, and trial sequential analysis (TSA) evaluated the association between tumor tissue miR-34a expression and survival outcomes in HNSCC. Following a protocol registered in PROSPERO (n. CRD420251238772), PubMed/MEDLINE, Scopus, ScienceDirect, CENTRAL, Google Scholar, and grey literature sources were searched for studies reporting overall survival (OS) or disease-free survival (DFS) stratified by miR-34a expression in HNSCC or its subsites. Hazard ratios (HRs) were extracted directly or reconstructed from Kaplan–Meier (KM) curves using the Tierney method, supported by a dedicated Python application (KM2HR). Four retrospective studies, corresponding to six study/site-specific cohorts and 318 patients, met the inclusion criteria. For OS (four cohorts), the fixed-effects model yielded a pooled HR of 2.25 (95% CI 1.48–3.41) for low versus high miR-34a expression, indicating worse survival in the low-expression group. However, the random-effects model attenuated the association (HR 1.32, 95% CI 0.32–5.54), with substantial heterogeneity (I^2^ ≈ 77%). For DFS (two studies), the fixed-effects model suggested poorer outcomes with low miR-34a (HR 2.92, 95% CI 1.24–6.88), whereas the random-effects model reversed the direction of effect with extremely wide confidence intervals (HR 0.19, 95% CI ≈ 0.00–129.34; I^2^ = 91%). TSA for OS (accrued information size 225 patients; estimated power ≈66%) crossed the monitoring boundary but did not reach the *a priori* information size, supporting only a tentative signal. A bioinformatic exploration of the TCGA HNSCC cohort (n = 522) showed a non-significant trend towards worse OS with low miR-34a (HR 1.24, 95% CI 0.93–1.65) and was excluded from pooling. Overall, low tumor miR-34a expression appears to be associated with poorer OS, but the evidence is limited by retrospective design, small sample size, and marked heterogeneity. miR-34a is a promising biomarker for prognostic stratification in HNSCC, yet larger, prospective, site-specific studies with standardized assays, pre-defined cut-offs, and appropriate adjustment for HPV status and clinical covariates are required before clinical implementation can be recommended.

## 1. Introduction

The head and neck squamous cell carcinoma (HNSCC) are a heterogeneous group of tumors originating from the mucosal epithelium and mainly affecting the oral cavity (OSCC), larynx (LSCC), hypopharynx (HSCC), nasopharynx (NPC), and oropharynx (OPSCC) [1]. Globally, HNSCC is the sixth most common malignancy, with approximately 960,000 new diagnoses annually (4.8% of all cancers), a five-year survival rate of around 30% in advanced stages (stages III–IV), and approximately 450,000 deaths annually (4.9% of global cancer mortality) [2].

Tobacco smoking and alcohol consumption are the main risk factors; furthermore, human papillomavirus (HPV) infection [3], particularly genotypes 16 and 18, is closely implicated in oropharyngeal carcinogenesis and characterizes subgroups with a generally more favorable prognosis, likely due to increased radiosensitivity [4].

The neoplastic transformation of HNSCC reflects the interaction of genetic and epigenetic alterations with environmental factors [5], with consequences on proliferation, invasion, migration, and cell death pathways.

Clinically, HNSCC prognosis is still largely driven by anatomical staging and a limited set of clinicopathological features; however, outcomes remain highly variable even within the same stage and treatment category [5]. Molecular biomarkers capable of capturing tumor biology (including proliferation, invasion, and treatment resistance) could, therefore, support risk stratification, treatment de-escalation/escalation decisions, and follow-up intensity in a more individualized way.

Nevertheless, differences in pre-analytical handling, assay platforms, normalization strategies, and cut-off definitions may substantially influence estimated prognostic effects; interpreting the literature, therefore, requires an explicit appraisal of these methodological sources of variability.

Between non-coding RNAs, microRNAs (miRs) are attractive candidates because they are relatively stable in formalin-fixed paraffin-embedded tissue and can be quantified with established laboratory workflows. In this context, miRs regulate gene expression at the transcriptional and post-transcriptional levels, modulating physiological and pathological processes [6,7]. Specific deregulation patterns are observed in head and neck cancers: some miRs are overexpressed (e.g., miR-155 [8], miR-196a, miR-196b [9], miR-1246 [10]), others are reduced (e.g., miR-375 [11], miR-101 [12], miR-29c [13]) and these profiles have been explored as biomarkers with prognostic value for survival [14].

Among the most interesting miRs, miR-34a a key node in the TP53-associated tumor suppressor circuit emerges as a biologically plausible candidate for prognostic stratification [15]: its reduced expression has been linked to more aggressive phenotypes and treatment resistance in several solid tumors [16].

The location of miR-34a is on chromosome 1 (1p36.22), specifically chr1:9151668–9151777 (− strand, GRCh38); its stem-loop (miRBase: MI0000268) sequence is ggccagcugugaguguuucuuUGGCAGUGUCUUAGCUGGUUGUugugagcaauaguaaggaagCAAUCAGCAAGUAUACUGCCCUagaagugcugcacguuguggggccc (uppercase segments correspond to the mature arms) [17]. It generates two mature forms: hsa-miR-34a-5p UGGCAGUGUCUUAGCUGGUUGU and hsa-miR-34a-3p CAAUCAGCAAGUAUACUGCCCU (miRBase MIMAT0000255 and MIMAT0004557).

In the context of HNSCC, the working hypothesis is that lower miR-34a levels are associated with worse overall survival (OS), regardless of site, quantification method, and HPV status. In light of this, the present study systematically evaluates the association between miR-34a expression and OS, exploring the main sources of clinical-biological and methodological heterogeneity.

## 2. Materials and Methods

### 2.1. Protocol

The planning of this systematic review and meta-analysis was conducted following the recommendations of the Cochrane Handbook for Systematic Reviews of Interventions [18]. Our manuscript was prepared in accordance with the PRISMA (Preferred Reporting Items for Systematic Reviews and Meta-Analysis) guidelines [19]. The protocol was registered on the PROSPERO platform (International Prospective Register of Systematic Reviews) under the registration number CRD420251238772 before proceeding with article selection.

### 2.2. Eligibility Criteria, Information Sources, Risk of Bias, Search and Selection

Then, we conducted a systematic review to evaluate whether miR-34a expression is associated with prognostic survival endpoints in HNSCC. Review question (PICO adapted for prognostic factor studies):

Population (P): adults with HNSCC (incl. OSCC, LSCC, HSCC, OPSCC, NPC).

Exposure/Index (I): altered miR-34a expression, operationalized as low vs. high tumor-tissue expression according to study-defined cut-offs.

Comparator (C): the opposite expression category (e.g., high when the index is low).

Outcomes (O): OS (Overall Survival) (primary); PFS (Progression Free Survival), RFS (RecurrenceFree Survival) DFS (Disease Free Survival), CSS (Cancer Specific Survival), as reported via HR (Hazard Ratio)/Cox models, relative risks, or Kaplan–Meier analyses.

Inclusion: primary studies of any design (randomized, non-randomized, prospective or retrospective cohorts/case-control) that report survival outcomes stratified by tumor tissue miR-34a expression in HNSCC or its histological subtypes.

Exclusion: No data on tumor tissue miR-34a expression in HNSCC (e.g., only circulating miR without tissue data); non-English publications; no prognostic survival endpoints (OS/PFS/RFS/DFS/CSS) or insufficient data to estimate an effect measure; reviews, case reports, case series (retained only for reference-checking).

Searches were run in PubMed/MEDLINE, Scopus, ScienceDirect, and the Cochrane Central Register of Controlled Trials (CENTRAL). We also screened Google Scholar and grey literature sources (e.g., OpenGrey) and checked reference lists of relevant reviews on miRNAs and HNSCC. No date limits were imposed at the search stage; non-English records were excluded during screening.

The following search strategies were employed across the selected databases: PubMed: Search: (miR-34a OR microRNA-34 OR miR34) AND (LSCC OR OPSCC OR OSCC OR HNSCC OR ORAL CANCER) Sort by: Most Recent:

(“miR-34a”[All Fields] OR “microRNA-34”[All Fields] OR “miR34”[All Fields]) AND (“LSCC”[All Fields] OR (“opscc”[All Fields] OR “opsccs”[All Fields]) OR “OSCC”[All Fields] OR (“hnsccs”[All Fields] OR “squamous cell carcinoma of head and neck”[MeSH Terms] OR (“squamous”[All Fields] AND “cell”[All Fields] AND “carcinoma”[All Fields] AND “head”[All Fields] AND “neck”[All Fields]) OR “squamous cell carcinoma of head and neck”[All Fields] OR “hnscc”[All Fields]) OR (“mouth neoplasms”[MeSH Terms] OR (“mouth”[All Fields] AND “neoplasms”[All Fields]) OR “mouth neoplasms”[All Fields] OR (“oral”[All Fields] AND “cancer”[All Fields]) OR “oral cancer”[All Fields])). Translations: OPSCC: “opscc”[All Fields] OR “opsccs”[All Fields];

HNSCC: “hnsccs” [All Fields] OR “squamous cell carcinoma of head and neck”[MeSH Terms] OR (“squamous”[All Fields] AND “cell”[All Fields] AND “carcinoma”[All Fields] AND “head”[All Fields] AND “neck”[All Fields]) OR “squamous cell carcinoma of head and neck”[All Fields] OR “hnscc”[All Fields];

ORAL CANCER: “mouth neoplasms”[MeSH Terms] OR (“mouth”[All Fields] AND “neoplasms”[All Fields]) OR “mouth neoplasms”[All Fields] OR (“oral”[All Fields] AND “cancer”[All Fields]) OR “oral cancer”[All Fields].

SCOPUS: TITLE-ABS-KEY ((miR-34a OR microRNA-34 OR miR34) AND (LSCC OR OPSCC OR OSCC OR HNSCC OR ORAL CANCER)).

The literature search closed on 10 October 2025 and was updated on 1 November 2025.

Data items were pre-specified by two reviewers and extracted as follows: bibliographic details (first author, year of publication), study setting (country), tumor site/subtype (OSCC, LSCC, HSCC, OPSCC, NPC) and sample size; patient and tumor characteristics (age, sex, smoking status, HPV positivity, follow-up duration, histologic grade, and stage); biomarker information (miR-34a plus any co-reported miRNAs together with the assay and the cut-off definition used to dichotomize low vs. high expression); and effect measures for prognostic endpoints (RR, Relative Risk or HR with 95% CIs for OS, PFS, RFS, DFS, and CSS).

When hazard ratios were available from both univariable and multivariable Cox models, we preferentially extracted the most fully adjusted estimate (along with its 95% confidence interval) to reduce confounding by established prognostic factors. If multiple miR-34a cut-offs were reported, we retained the cut-off designated by the original authors as primary (typically based on ROC analysis or median split), and we recorded the cut-off definition to support qualitative interpretation of heterogeneity.

In cases where only Kaplan–Meier survival curves were available, the hazard ratio was calculated using the Tierney method [20] extracting data from the curves using an application (KM2HR—Kaplan–Meier HR Extractor|Dioguardi Mario—University of Foggia, version 2.1) built by the authors in a python environment. The application is also available as Supplementary Material.

The KM2HR (2.1)—Kaplan–Meier HR Extractor was developed in Python (3.13.0) with a Tkinter GUI (8.6), using matplotlib (3.10.3) for visualization, numpy (2.2.6)/pandas (2.2.3) for numerical and tabular processing, openpyxl (3.1.5) for Excel export, and python-docx (1.1.2) for Word reporting.

A Kaplan–Meier (KM) curve image is loaded and calibrated; users digitize points for two arms (step functions enforced to be monotonically non-increasing) and may optionally input numbers-at-risk (NAR) at prespecified times.

Event counts and risk sets are reconstructed interval-by-interval from survival drops, constraining to the provided NAR where available; in the absence of NAR, light censoring is applied on flat segments to maintain plausible risk-set decay.

The treatment effect follows the Parmar–Tierney framework: the log-rank components O–E (observed minus expected) and V (its variance) are accumulated across intervals.

Effect estimates are computed as log (HR) = OE/V, SE = 1/√V, HR = exp (log (HR)), with 95% CIs = exp (log(HR) ± 1.96·SE).

The principal assumption is proportional hazards (on average over time).

Outputs include a Word report (figure, HR, tables) and an Excel workbook (summary: HR, logHR, SE, OE, V; digitized curves; interval grid; NAR).

The KM2HR tool was further assessed through a two-step validation process. First, an internal computational verification was performed to confirm the correct implementation of the formulae used for hazard ratio reconstruction. The exported O–E and V values were used to independently recalculate the logHR, standard error, HR, 95% confidence intervals, and reciprocal HRs, thereby verifying the internal consistency of the calculations.

Second, the KM2HR workflow was externally benchmarked using Kaplan–Meier curves for which published or displayed HR values were available. This benchmark set included curves from published studies on miRNAs in head and neck cancer and HNSCC curves derived from the TCGA/KM Plotter database. For each benchmark curve, the digitized Kaplan–Meier coordinates, numbers-at-risk tables, HR direction, assumptions used for risk-set reconstruction, reconstructed HRs, and degree of agreement with the published or displayed HR were recorded.

The complete HR reconstruction workflow and validation process are provided in the Supplementary Materials, including the KM2HR Python source code, digitized curve data, numbers-at-risk tables, Excel formula-check worksheets, validation benchmark tables, and KM2HR exported reports.

Additionally, the TCGA (The Cancer Genome Atlas) database, containing a cohort of patients with HNSCC, was consulted to extract HR values related to prognostic indices associated with miR-34a expression. These values were excluded from the meta-analysis to avoid bias due to data heterogeneity, as the TCGA cohort included approximately 522 patients, which was four times larger than the largest population study.

The risk of bias in individual studies was assessed by two authors (M.D. and S.C.) using evaluation criteria derived from the REMARK (Reporting Recommendations for Tumor Marker Prognostic Studies) protocol. Studies with a high risk of bias were excluded from the meta-analysis.

Heterogeneity among studies was assessed using Higgins’ index (I^2^) and the Chi^2^ test. For the meta-analysis, particularly for calculating the aggregated HR or RR, the Reviewer Manager 5.4 software (Cochrane Collaboration, Copenhagen, Denmark) was used.

The quality of evidence was assessed using the online software GRADEpro Guideline Development Tool (GRADEpro GDT, Evidence Prime, Hamilton, ON, Canada, https://gdt.gradepro.org/app/ access data: 10 November 2025).

Small study effects were assessed using the Doi plot and LFK index [21], which are more appropriate than *p* value–based methods for meta-analyses including a limited number of studies. The Trial Sequential Analysis (TSA) was performed using Stata 15 software (StataCorp, College Station, TX, USA) with the implementation of R 4.5 software and the installation of the id-bounds and metacumbounds commands.

## 3. Results

### 3.1. Selection of Studies

The searches conducted in ScienceDirect, SCOPUS, PubMed, and Cochrane Central Trial yielded 137 bibliographic sources. After removing duplicates, 95 potentially eligible articles remained. Of these, only 8 articles underwent full-text screening, and only 4 fully met the inclusion and exclusion criteria. Consequently, the relevant extracted data from these 4 studies were included in the meta-analysis.

Additionally, grey literature searches (http://www.opengrey.eu, accessed on 1 November 2025), Google Scholar, Data Archiving and Networked Services (DANS) EASY Archive and previous systematic reviews using “miR-34a” as a keyword did not identify any further studies eligible for inclusion in the meta-analysis.

Furthermore, a TCGA analysis was performed using the Kaplan–Meier Plotter database portal (https://kmplot.com/analysis/, accessed on 1 November 2025), and HR data were extracted.

**Figure 1:**
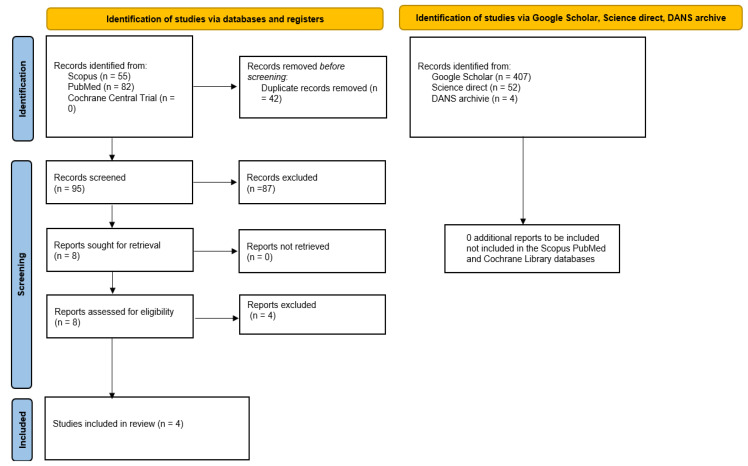
Entire selection and screening procedures are described in the PRISMA flowchart.

The complete procedure for the identification, selection, and inclusion of studies is outlined in the flowchart shown in Figure 1.

### 3.2. Characteristics of the Data and Included Studies

The systematic review included four retrospective studies: Shen et al., 2012 [22]; Ogawa et al., 2012 [23]; Piotrowski et al., 2021 [24]; and Ren et al., 2023 [25]. The total number of patients with HNSCC across the studies was 318; among these, 183 cases were OSCC, 102 were LSCC, 24 were sinonasal squamous cell carcinomas, and 9 were OPSCC (Table 1).

Overall survival and disease-free survival were the most frequently reported prognostic endpoints, whereas no data were available for relapse free survival (RFS), cancer specific survival, or progression free survival. Piotrowski et al., 2021 [24] reported OS separately for three patient groups (37 OSCC, 9 OPSCC, and 33 LSCC), which were analyzed as distinct cohorts in the meta-analysis.

Two studies provided Kaplan–Meier survival curves specifically, the OPSCC cohort in Piotrowski et al., 2021 [24] and the study by Ren et al., 2023 [25] for which hazard ratios (HRs) were not available. Survival data and HRs were, therefore, extracted using the Tierney method [9], and the resulting estimates are presented in Table 2.

For Shen et al. [22], the DFS estimate of 4.02 (1.67–9.69; low vs. high) was taken from two prior reviews Dioguardi et al. [8] and Huang et al., 2021 [26] because the original study provided only a Kaplan–Meier curve; given that a published estimate already existed, we did not re-derive an HR using the Tierney method in this instance [20,27]. For Ogawa et al., 2012 [23], CSS could not be estimated because there were no events in the high-expression group.

Regarding risk factors, alcohol consumption and smoking were investigated only by Ren et al., 2023 [25] (85 smokers and 81 habitual alcohol consumers among 146 OSCC), whereas HPV status was explicitly addressed only by Piotrowski et al., 2021 [24], in which HPV-positive malignancies were listed among the exclusion criteria. Therefore, harmonized HPV/p16 data were not available across the included studies, and subgroup analyses according to HPV status could not be performed. However, no additional data were available on the number of cigarettes smoked or the amount of alcohol consumed.

Shen et al., 2012 [22] did not report the sex of enrolled patients; among the remaining cases, 183 were male and 66 were female. Consequently, the REMARK-based risk-of-bias assessment for Shen et al., 2012 [22] received a lower score than the other studies. The estimated mean patient age was approximately 59.8 years.

All four studies were published between 2012 and 2023. The unweighted mean follow-up was 66 months, with values ranging from approximately 50 to 150 months. All survival prognostic indices included in the meta-analysis were extracted and are summarized in Table 2.

### 3.3. Risk of Bias in Studies

The risk of bias in the included studies was assessed using a classification derived from the REMARK guidelines [28]. Specifically, the REMARK-based appraisal was structured into six domains: sample description, clinical data, marker quantification, prognostic endpoint reporting, statistical analysis, and adjustment for classical prognostic factors. Each domain was assigned a score from 1 to 3: 1 indicated inadequate reporting or a high level of concern, 2 indicated partially adequate reporting or some concern, and 3 indicated adequate reporting or a low level of concern. The total score therefore ranged from 6 to 18, with higher scores indicating better methodological quality. Scores between 15 and 18 were considered to indicate high methodological quality, scores between 11 and 14 intermediate quality, and scores between 6 and 10 low methodological quality. The REMARK-based assessment was used to guide the interpretation of risk of bias and GRADE certainty, and was not applied as an automatic exclusion criterion after eligibility screening (Table 3).

Below are the principal shortcomings of the studies, according to the REMARK based appraisal of the prognostic data considered in this systematic review. Two studies showed major issues:

1. Shen et al., 2012 [22], deficiencies in the Statistics and Classical prognostic factors domains (score 1, Inadequate). A multivariable Cox model is mentioned with coefficients and *p* values, but hazard ratios with 95% confidence intervals for the high- versus low-expression comparison are not reported; therefore, HRs must be reconstructed from the Kaplan–Meier curves (an estimate has, in fact, been provided in previous systematic reviews [8]). For Classical prognostic factors, a DFS T stage correlation is cited, but it is unclear whether and how the model adjusted for established covariates (age, full TNM stage, nodal status, sex); the variables included are not specified. In addition, patient sex is not reported under clinical data.
2. Ren et al., 2023 [25] deficiencies in the Statistics and Classical prognostic factors domains. As above, HRs need to be extracted from Kaplan–Meier curves (Statistics), and no multivariable survival modelling adjusting for age and stage was performed.

### 3.4. TCGA Cohort Analysis Results

**Figure 2:**
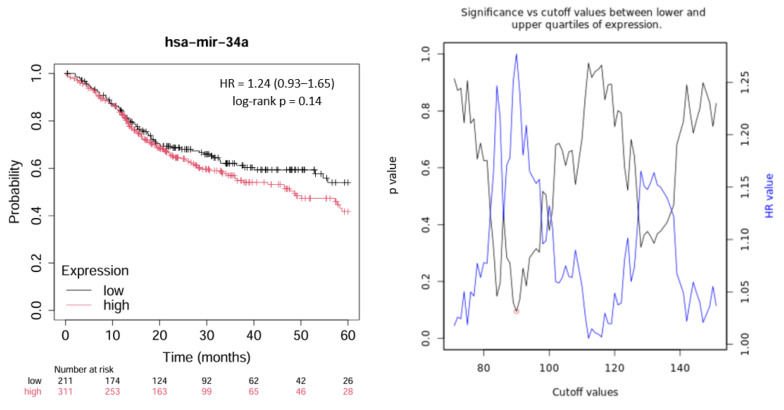
hsa-miR-34a. Kaplan–Meier curves for overall survival (OS) according to miR-34a expression in patients with HNSCC (TCGA cohort). Curves were generated using the public Kaplan–Meier Plotter web application (http://kmplot.com/analysis/; accessed 10 November 2025). The figure and underlying data can be readily reproduced via the portal. Auto cut-off plot. Automatically generated cut-off selection from the Kaplan–Meier Plotter (http://kmplot.com/analysis/; accessed 10 November 2025). Significance versus candidate cut-off values across the lower to upper quartiles of expression is shown; red circle: optimal.

Using the Kaplan–Meier Plotter portal (kmplot.com; accessed 10 November 2025), we interrogated the TCGA HNSCC cohort (n = 522) to visualize survival according to miR-34a expression (Figure 2). The platform dichotomizes samples into “low” and “high” groups via a data-driven threshold; the selected cut-off and its associated *p*-value are reported in Supplementary Material.

Briefly, the portal scans candidate cut-offs across the expression distribution (from the first to the fourth quartile) and fits a Cox proportional hazards model at each step [29]. The cut-off linked to the smallest *p*-value is used as the default threshold for group assignment. Where multiple cut-offs produced comparably low *p*-values, we retained the setting yielding the largest absolute hazard ratio. Because this approach involves repeated testing, the portal also computes a false discovery rate using the Benjamini–Hochberg procedure to control for multiplicity [30].

The tool then provides the resulting Kaplan–Meier plot using the chosen threshold (Figure 2), together with a visual summary of *p* values across candidate cut-offs, facilitating an appraisal of the robustness of the selection.

The bioinformatics analysis of miR-34a reported an HR for OS between low and high expression groups of 1.24 CI 95% [0.93–1.65], with a log-rank *p*-value of 0.14. The median survival for the low-expression cohort was 17.37 months, while it was 15.2 months for the high-expression cohort.

The bioinformatic analysis performed through the TCGA portal is presented separately as an exploratory external comparator and contextual validation analysis, rather than as part of the primary pooled evidence. The HR reported by the Kaplan–Meier Plotter for miR-34a in the TCGA HNSCC cohort (HR 1.24; 95% CI 0.93–1.65; *p* = 0.14) was considered and compared as a reference estimate for the direction and approximate magnitude of the association observed in the published clinical cohorts. Although this result was not statistically significant, it showed a trend towards poorer overall survival in the low miR-34a expression group, consistently with the fixed-effect pooled estimate.

Including this single large database-derived cohort in the pooled estimate would also have dominated the analysis and introduced substantial methodological heterogeneity.

A limitation of this analysis is that the log-rank test *p*-value is above 0.05, indicating low statistical significance (Figure 2).

In addition, selected TCGA/KM Plotter HNSCC curves were used for methodological benchmarking of the KM2HR tool, because the platform provides Kaplan–Meier curves, numbers at risk and displayed HR estimates. These curves allowed comparison between KM2HR-reconstructed HRs and the corresponding displayed HRs. This technical validation step was kept separate from the clinical meta-analysis.

### 3.5. Meta-Analysis, Sensitivity Analysis, Subgroup Analysis, Publication Bias

The meta-analysis of the data was conducted using Review Manager 5.4 software (Cochrane Collaboration, Copenhagen, Denmark), which was also used to generate the forest plot and funnel plot images.

**Figure 3:**
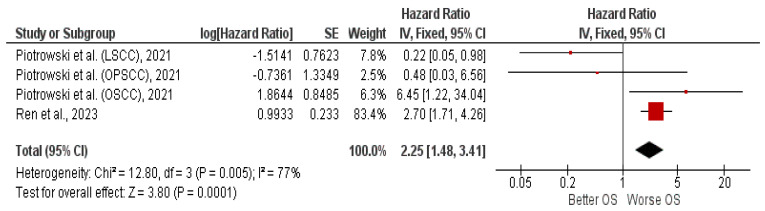
Forest plot of the fixed-effects model of the meta-analysis: OS, HR = 2.25. 95% CI: [1.48, 3.41]; df = degrees of freedom; I^2^ = Higgins’ heterogeneity index (I^2^ < 50% indicates irrelevant heterogeneity; I^2^ > 75% indicates significant heterogeneity); CI = confidence intervals; P = *p*-value; SE = standard error; IV = inverse-variance and Z Wald z-test statistic used to test the overall pooled effect. The graph for each study shows the lead author and year of publication, the hazard ratio (HR) with confidence intervals, the log HR standard error, and the weight of each study expressed as a percentage. The final value is expressed in bold with the corresponding confidence intervals. The black line indicates the position of the average value, and the light black diamond represents the measure of the average effect; Red squares represent the individual study hazard-ratio estimates, with the size of each square proportional to the study weight. The final effects consistently favor a worsening of OS in patients with low tissue expression of miR-34a; Piotrowski et al., 2021 [24], Ren et al., 2023 [25].

The meta-analysis focused on the HR for OS between low and high tissue expression of miR-34a. Fixed effects were applied, and the log hazard ratio and standard deviation (SD) between the two groups (low and high expression) were calculated. The aggregated HR value was 2.25 [1.48, 3.41], indicating a worsening of OS in patients with low miR-34a expression. The black diamond in the forest plot, representing the effect size, narrowly missed the line of no effect (Figure 3).

The studies included in the meta-analysis per la OS were Piotrowski et al., 2021 [24] (with three distinct cohorts of patients, stratified according to the type of HNSCC) and Ren et al., 2023 [25].

Only two cohorts of patients (LSCC and OPSCC) di Piotrowski et al., 2021 [24] crossed the line of no effect with their confidence intervals.

**Figure 4:**
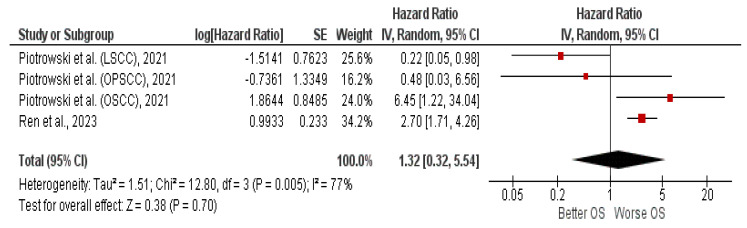
Random-effects meta-analysis of the association between low miR-34a expression and overall survival in patients with head and neck squamous cell carcinoma. Forest plot of HR for OS comparing patients with low versus high miR-34a expression. Squares represent study-specific HR, with their size proportional to the inverse-variance weight; horizontal lines indicate 95% confidence intervals. The diamond denotes the pooled HR (random-effects model). An HR > 1 indicates worse overall survival in the low miR-34a expression group. CI = confidence intervals; P = *p*-value; SE = standard error; IV = inverse-variance and Z Wald z-test statistic used to test the overall pooled effect. Heterogeneity: OS, HR = 1.32. 95% CI: [0.32, 5.54]; Tau^2^ = 1.51; Chi^2^ = 12.80, df = 3 (*p* = 0.005); I^2^ = 77%; Piotrowski et al., 2021 [24], Ren et al., 2023 [25].

In addition, a random-effects analysis was performed, whereby the HR for OS decreased to 1.32 [0.32, 5.54] (Figure 4), indicating only a trend towards worse OS in patients with low miR-34a expression. It should be noted that the central diamond crosses the line of no effect, indicating a lack of statistical significance, while the heterogeneity quantified by the I^2^ inconsistency index remained unchanged at 77% despite the use of a random-effects model.

**Figure 5:**
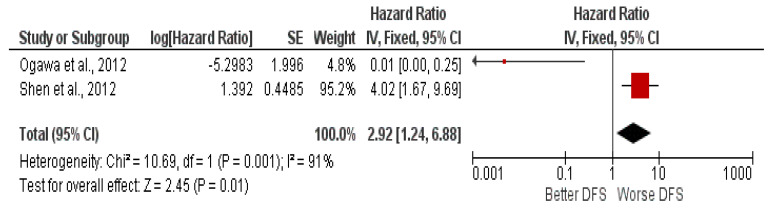
Fixed-effects meta-analysis of the association between low miR-34a expression and DFS. Red squares represent the individual study hazard-ratio estimates, with the size of each square proportional to the study weight. The black diamond represents the pooled effect estimate and its 95% confidence interval. Bold font indicates the overall pooled estimate and related summary statistics; DFS, HR = 2.92. 95% CI: [1.24, 6.88]; Chi^2^ = 10.69, df = 1 (*p* = 0.001); I^2^ = 91%. CI = confidence intervals; P = *p*-value; SE = standard error; IV = inverse-variance and Z Wald z-test statistic used to test the overall pooled effect; Ogawa et al., 2012 [23], Shen et al., 2012 [22].

**Figure 6:**
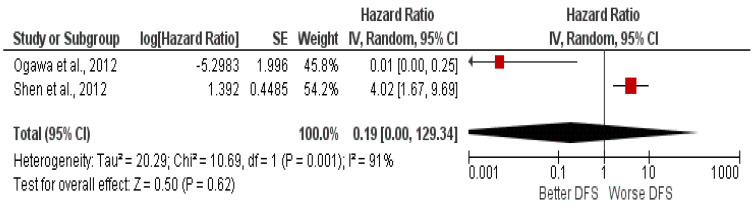
Random-effects meta-analysis of the association between low miR-34a expression and DFS. Red squares represent the individual study hazard-ratio estimates, with the size of each square proportional to the study weight. The black diamond represents the pooled effect estimate and its 95% confidence interval. Bold font indicates the overall pooled estimate and related summary statistics; DFS, HR = 0.19. 95% CI: [0.00, 129.34]; Tau^2^ = 20.29 Chi^2^ = 10.69, df = 1 (*p* = 0.001); I^2^ = 91%. CI = confidence intervals; P = *p*-value; SE = standard error; IV = inverse-variance and Z Wald z-test statistic used to test the overall pooled effect; When a fixed-effects model is applied, the result is driven almost entirely by the Shen et al., 2012 [22], whereas under a random-effects model the weights become more balanced and the mean effect is essentially cancelled out because of the high heterogeneity; Ogawa et al., 2012 [23].

For the second meta-analysis, which investigated the HR for DFS comparing low versus high miR-34a expression, data from Shen et al. (2012) [22] and Ogawa et al. (2012) [23] were pooled, and both fixed-effects (Figure 5) and random-effects models (Figure 6) were applied. Using a fixed-effects model, the pooled HR was 2.92 (95% CI: 1.24–6.88), indicating a worse prognosis in patients with low miR-34a expression. By contrast, with the random-effects model the HR reversed to 0.19 (95% CI: 0.00–129.34), crossing the line of no effect.

With the fixed-effects model, the result is effectively dominated by the most precise study (Shen et al., 2012) [22], because the weights are inversely proportional to the variance. Under the random-effects model, however, the very high heterogeneity increases τ^2^, making the study weights more similar; as a consequence, the opposing study signals counterbalance each other and the pooled effect is null, with very wide confidence intervals.

The DFS analysis should be interpreted with particular caution. Only two studies were available, their effect estimates were in opposite directions, and heterogeneity was very high (I^2^ = 91%). Therefore, the DFS findings are exploratory, statistically unstable, and should not be used to support firm conclusions regarding the prognostic value of miR-34a.

**Figure 7:**
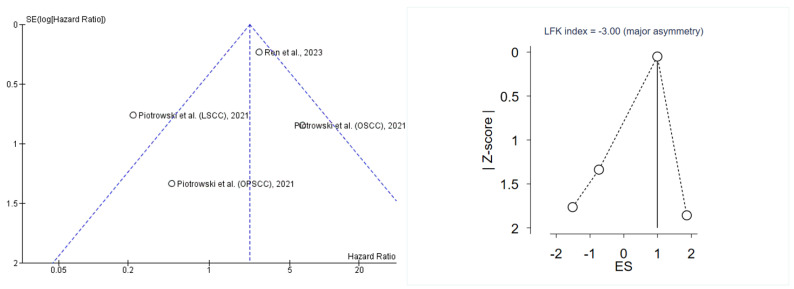
Funnel plot and DOI plot for the meta-analysis HR OS miR-34a. Left: Funnel plot of log(HR) versus SE[log(HR)] for four comparisons (Piotrowski: LSCC/OPSCC/OSCC; Ren: OSCC). The vertical dashed line marks the pooled effect; slanted dashed lines denote the 95% pseudo-confidence limits. Right: Doi plot (effect size = log[HR] on the x-axis; |Z-score| on the y-axis) showing LFK index = −3.00, consistent with major left-sided asymmetry. Ogawa et al., 2012 [23], Shen et al., 2012 [22], Piotrowski et al., 2021 [24], Ren et al., 2023 [25].

A visual assessment of publication bias was also performed by inspecting the distribution of studies in funnel plots (Figure 7). An asymmetry was observed, which is more likely to be attributable to clinical and methodological heterogeneity (differences in tumor site and cut-off values) than to genuine publication bias. Owing to the small number of studies and, in particular, the high heterogeneity, the Egger test was considered underpowered and unreliable (high risk of false negatives) and was, therefore, not used for inference. For the OS endpoint (k = 4), the Egger test yielded an intercept of −1.51 (SE 1.82), t(2) = −0.83, *p* = 0.49, 95% CI −9.36 to 6.33. There was thus no statistical evidence of small-study effects; however, given the limited number of comparisons and the substantial heterogeneity (I^2^ ≈ 77%), this result should be regarded as inconclusive rather than as proof of absence of bias [31].

Using the Doi plot with LFK index (implemented in STATA 15; the LFK index quantifies Doi plot asymmetry, with |LFK| < 1 indicating no asymmetry, 1–2 minor asymmetry and >2 major asymmetry), the pattern again suggested that small-study effects might be present. Nonetheless, given the small number of comparisons and the marked clinical heterogeneity (different primary sites, cut-offs/assays and HPV status), it is more plausible that the asymmetry reflects genuine between-study heterogeneity rather than publication bias (Figure 7).

### 3.6. Trial Sequential Analysis

Trial sequential analysis (TSA) was conducted to evaluate the robustness of the meta-analysis results, adjusting them to minimize type I and type II errors. The analysis was performed using Stata 15 (StataCorp, College Station, TX, USA) integrated with R 4.5 software through the ‘metacumbounds’ commands, as described by Miladinovic et al. [32]. The O’Brien–Fleming spending function was applied under a random-effects model.

**Figure 8:**
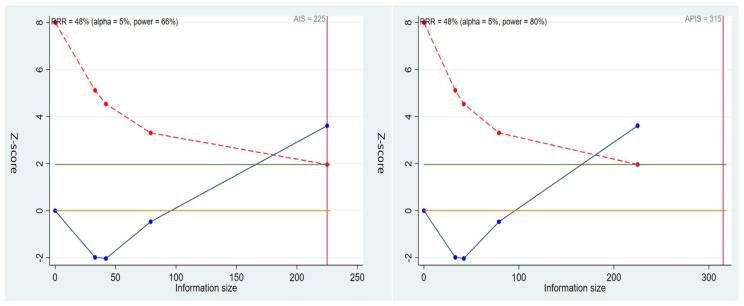
AIS, light green line (Z = 1.98); dashed red line (monitoring boundary); blue line (cumulative z curve); red line (sample size). APIS, light green line (Z = 1.98); dashed red line (monitoring boundary); blue line (cumulative z curve); red line (sample size). The yellow horizontal line indicates the null-effect line, corresponding to Z = 0, and separates the positive and negative directions of the cumulative Z-curve.

The APIS (*a priori* information size) and AIS (accrued information size) commands were utilized via the dialog box to determine the optimal sample size and the power of the results. Assumptions included an average survival rate of 49% [33], a relative risk reduction (RRR) of 48.5%, based on previous studies evaluating prognostic factors in HNSCC [34], an alpha value of 5% (type I error), and a beta value of 20% (type II error). (Figure 8).

The accrued information size (AIS) represents the number of participants actually accumulated in the cumulative meta-analysis, whereas the a priori information size (APIS) represents the required sample size calculated on the basis of the pre-specified assumptions regarding alpha, beta, expected survival rate and relative risk reduction. In the present analysis, the AIS was 225 patients, whereas the APIS was 315 patients; therefore, the planned information size was not reached.

The TSA was performed assuming an average survival rate of 49%, a RRR of 48% between the low and high miR-34a expression groups, an alpha level of 5% and a beta level of 20%. According to the Lan–DeMets trial sequential monitoring framework described by Miladinovic et al., crossing the monitoring boundary before reaching the required information size may suggest early evidence of an effect [32]. In the present analysis, the cumulative Z-curve crossed the trial sequential monitoring boundary, indicating that the predefined effect was observed in the available data. However, the accrued information size remained below the a priori information size, and the estimated power was approximately 66%, below the conventional 80% threshold. Therefore, the total number of participants required to reach the planned information size was not achieved. Consequently, the TSA findings should be interpreted as supporting only a preliminary signal and should not be considered conclusive evidence of a prognostic effect.

### 3.7. Grade

The authors also used GRADE pro-GDT to assess the quality of the evidence. (Table 4). The certainty of evidence was assessed using the GRADE approach. For OS, the certainty was rated as low because the evidence derived from retrospective cohorts and was affected by risk of bias, substantial inconsistency, and suspected small-study effects. For DFS, the certainty was rated as very low because only two studies were available, the study-specific estimates showed opposite directions, heterogeneity was very high and the confidence interval under the random-effects model was extremely wide.

## 4. Discussion

A systematic review with meta-analysis and trial sequential analysis (TSA) was conducted to investigate the potential use of altered miR-34a expression as a prognostic biomarker for survival in HNSCC. This systematic review represents the first TSA-based meta-analysis focusing on miR-34a in HNSCC and included four studies and seven study/site-specific cohorts, in addition to the TCGA cohort, which comprises approximately 522 patients with HNSCC.

Overall, 318 patients from the four studies were included in the meta-analysis. To minimize publication bias, the grey literature was also searched. TSA results indicated that, assuming a 48% relative risk reduction (RRR) between low- and high-expression groups, the meta-analysis reached adequate statistical power to cautiously support a genuine prognostic role, although the APIS boundary line was not crossed. Several studies have shown that miR-34a may be downregulated in tumor tissues and under expressed in many solid cancers, with its overexpression generally associated with inhibition of tumor growth [35,36,37,38,39].

### 4.1. Role of miR-34a in Molecular Regulation and Tumor Invasiveness in HNSCC

The miR-34 family, comprising three members (miR-34a, miR-34b, and miR-34c), is involved in regulation of the cell cycle, downregulation of epithelial–mesenchymal transition (EMT), suppression of the stem-like phenotype, induction of apoptosis and senescence, and inhibition of glycolysis. From a biological standpoint, miR-34a is widely regarded as a key downstream effector of TP53 signaling. Accordingly, reduced miR-34a expression may represent a functional readout of TP53 pathway disruption, which is frequent in HNSCC through somatic TP53 alterations and, in HPV-driven tumors, through viral oncoprotein–mediated TP53 inactivation. This framework offers a plausible explanation for between-study differences linked to tumor site and HPV status, and underscores the importance of reporting HPV/p16 status in prognostic biomarker studies.

In particular, miR-34a is part of this family, and evidence from tumor cell lines clearly shows its downregulation, as reported by Gaur et al. in 2007 [40], making it a plausible candidate biomarker. Shen et al. (2012) [22] reported interesting findings regarding the possible role of miR-34a in targeting survivin: miR-34a reduced survivin expression and significantly suppressed cell proliferation. Moreover, in LSCC specimens, miR-34a levels were downregulated whereas survivin expression was upregulated, and a positive correlation was observed between survival rate and miR-34a upregulation.

By contrast, Piotrowski et al. [24] observed miR-34a-5p upregulation in LSCC, in disagreement with Shen et al., [22] and a worse prognosis in patients with miR-34a downregulation [24]. Specifically, Piotrowski reported that miR-34a alone does not achieve the predefined sensitivity/specificity thresholds required to serve as a “strong” tumor–normal discrimination biomarker, despite being upregulated in OSCC compared with normal tissue, and that its prognostic meaning varies according to tumor site: protective in OSCC and unfavorable in LSCC [24].

Studies by Ogawa et al., performed on both cell lines and tissue samples, further support miR-34a as a candidate prognostic biomarker. Ogawa proposed that miR-34a regulates genes on which TP53 exerts its tumor-suppressor functions, and that these downstream genes controlled by miR-34a influence survival outcomes [23]. Consistent with this hypothesis, his data showed lower miR-34a expression in tumors that subsequently recurred. Indeed, as highlighted by Hermeking, p53 regulates the expression not only of protein-coding genes but also of non-coding RNAs, including miRNAs; among these, members of the miR-34 family are directly induced by p53 [41].

Ren et al., 2023 [25] demonstrated that miR-34a expression is reduced in tissues and cell lines derived from highly aggressive OSCC. In particular, miR-34a can decrease PA28γ expression and inhibit OSCC invasion and migration. They further confirmed that the circFANCA/miR-34a/PA28γ axis facilitates cellular invasion and migration and enhances the metastatic potential of OSCC cells through a sponge effect on miR-34a.

In addition to effects on tumor-cell intrinsic programs, miR-34a has been implicated in pathways relevant to the tumor microenvironment, including cellular senescence, inflammatory signaling, and immune evasion. If confirmed in HNSCC, these mechanisms could help explain why miR-34a levels might correlate not only with survival but also with patterns of relapse and response to adjuvant therapies. Future clinical studies would benefit from integrating miR-34a assessment with contemporaneous information on treatment modalities (surgery, radiotherapy, systemic therapy) and immune-related markers to better contextualize observed prognostic associations.

### 4.2. Meta-Analysis and TSA

The meta-analysis based on the HR for OS yielded a pooled fixed-effects estimate of HR = 2.25. 95% CI: [1.48, 3.41], which was largely driven, in terms of weight, by the study with the narrowest confidence intervals (Ren et al.), indicating poorer survival in patients with low miR-34a expression. When a random-effects model was applied, the pooled HR shifted towards the null HR 1.32, 95% CI: [0.32, 5.54] and only suggested a trend towards a worse prognosis, with increased uncertainty. This was reflected by Higgins’ inconsistency index (I^2^), which remained high at 77% and did not change under the random-effects model. Egger’s test and the Doi plot with LFK index, used purely for methodological purposes to explore publication bias, were inconclusive because of the insufficient number of studies and were therefore not considered informative. Sensitivity analyses and subgroup analyses were not performed, as the small number of included cohorts was deemed inadequate for these approaches.

The pooled OS estimate should be interpreted with caution because it combines clinically and biologically heterogeneous HNSCC subsites, including OSCC, LSCC, OPSCC, and sinonasal squamous cell carcinoma. These tumors differ in anatomical origin, aetiology, HPV/p16 association, treatment pathways and prognosis, and these factors may modify the prognostic effect of miR-34a. Because of the small number of available cohorts and incomplete HPV/p16 reporting, reliable site-specific subgroup analyses, HPV/p16-stratified analyses or meta-regression could not be performed. Therefore, the pooled estimate should be regarded as exploratory and hypothesis-generating.

The TCGA cohort, which was not included in the meta-analysis, provided results consistent with our findings, showing an HR for OS of 1.24 95% CI: [0.93, 1.65]. These TCGA data were considered statistically significant by the original authors and support a more favorable prognosis in patients with higher miR-34a expression.

For the second meta-analysis, which evaluated DFS and included only two studies reporting opposing HR estimates, the fixed-effects model favored a worsening of DFS in the presence of low miR-34a expression (predominantly driven by the weight of the Shen et al. study; HR 2.92, 95% CI: [1.24, 6.88], whereas the pooled effect reversed under the random-effects model (HR 0.19, 95% CI: [0.00, 129.34].

Accordingly, the DFS findings should be regarded as exploratory only. Given the very small number of studies, the opposite direction of the individual estimates and the very high heterogeneity, these results cannot be used to support a firm prognostic conclusion for miR-34a.

However, TSA (performed on the four patient cohorts, i.e., on the HR for OS) confirmed that, although the APIS of 315 patients was not reached, the AIS of 225 patients and an estimated power of 66% nonetheless provided sufficient statistical power to suggest that the effect is likely to be genuine. The certainty of the evidence, assessed using the GRADE framework, was rated as low for both meta-analyses, mainly due to the retrospective nature of the included studies and the absence of randomized controlled clinical trials.

From a clinical perspective, an HR in the range suggested by the fixed-effects model would be potentially meaningful for stratifying postoperative surveillance or adjuvant treatment discussions; however, the instability of the estimate under random-effects modeling highlights that the current evidence is not yet sufficiently consistent for clinical implementation. A further contributor to heterogeneity is the common practice of dichotomizing continuous miR expression using study-specific thresholds, which can amplify apparent effects and limit comparability. Reporting miR-34a as a continuous predictor (or using standardized, pre-registered cut-offs) and presenting both adjusted and unadjusted models would substantially improve interpretability and future evidence synthesis.

### 4.3. Risk Factors

From the analysis of the included studies, no robust data on risk factors emerged. In fact, only Ren et al. [25] reported the number of alcohol consumers and smokers, whereas Piotrowski stated that HPV16-positive patients were excluded from the analysis [24].

However, a broader examination of the literature provides some relevant insights. Kalfert et al. (2015) showed that, in OPSCC, high miR-34a expression levels were associated with p16-positive cases (a surrogate marker for HPV16) [42]. In addition, Wu et al. (2021), through a TCGA-based analysis, identified TP53 mutations and 1p loss as being associated with low miR-34a levels; miR-34a acts as a tumor suppressor (targeting MET) [43]. Since HPV-negative forms (more often related to smoking and alcohol consumption) more frequently TP53 mutations, this study plausibly links classical risk factors to a reduction in miR-34a via TP53.

### 4.4. Limitations

This review has several important limitations:

First, the evidence base is limited and entirely retrospective: only four studies (seven cohorts), comprising a total sample of 318 patients, were eligible, and some cohorts were very small (for example, OPSCC n = 9; sinonasal n = 24). This limits precision and increases the risk of small-study effects.

Second, clinical and methodological heterogeneity was substantial. Some studies did not distinguish between anatomical subsites (OSCC, LSCC, OPSCC, sinonasal), used different assays and normalization strategies for miR-34a, and adopted heterogeneous, study-defined cut-offs; HPV status and other risk factors were reported inconsistently (for example, exclusively HPV-positive in one study, only smoking/alcohol in another); these subsites differ in biology, HPV/p16 association, prognosis, and treatment pathways. Owing to the small number of cohorts and incomplete HPV/p16 reporting, site-specific and HPV/p16-stratified analyses were not feasible. This resulted in marked inconsistency for overall survival (I^2^ ≈ 77%) and divergent results between fixed- and random-effects models; for DFS, the cumulative effect under the fixed-effects model reversed direction under the random-effects model, underscoring its fragility.

Third, incomplete reporting in the primary studies necessitated indirect estimation of effect sizes. HRs had to be reconstructed from Kaplan–Meier curves for some cohorts using the method described by Tierney.

Assessment of small-study effects/publication bias was underpowered. While Egger’s test was not statistically significant, the Doi plot yielded an LFK index consistent with major asymmetry; given the very small k and pronounced heterogeneity, these patterns are difficult to interpret and do not rule out reporting bias.

The TSA was based on assumptions (baseline OS ≈ 49%, pre-specified relative risk reduction ≈ 48.5%, α = 5%, β = 20%) and crossed the monitoring boundaries before the *a priori* information size was reached. The accrued information size remained below the target; therefore, the TSA findings must be interpreted with caution.

The bioinformatic exploration of the TCGA HNSCC cohort (using an automatic cut-off approach) yielded a non-significant association for OS (HR ≈ 1.24; *p* ≈ 0.14) and was excluded from the meta-analysis to avoid domination of the pooled estimate.

Finally, the limited number of eligible cohorts precluded planned subgroup, sensitivity, and meta-regression analyses (for example, by site, HPV status, assay/cut-off, treatment), which would have been necessary to account for heterogeneity and assess effect modification. Consequently, the certainty of the evidence rated using GRADE ranged from low to very low, due to risk of bias, inconsistency, and imprecision.

Additional limitations include potential residual confounding and differences in covariate adjustment across cohorts, because some primary studies reported only unadjusted survival analyses. Moreover, variations in tissue handling (fresh-frozen vs. FFPE), RNA extraction kits, and reference genes used for normalization can introduce systematic measurement differences that are rarely quantified. Finally, selective reporting (e.g., focusing on the most favorable cut-off or endpoint) cannot be excluded in a small evidence base, reinforcing the need for prospective protocols, transparent reporting, and data sharing to enable individual-participant data meta-analysis.

Overall, these limitations indicate that any prognostic signal of tissue miR-34a in HNSCC should be considered hypothesis-generating. Larger, prospective, site-specific studies with standardized assays, pre-defined cut-offs, comprehensive covariate adjustment (including HPV) and harmonized treatments are required to confirm or refute the association observed.

## 5. Conclusions

Low tissue expression of miR-34a was associated with poorer overall survival in the fixed-effect model (HR 2.25; 95% CI 1.48–3.41). However, this association was no longer statistically significant under the random-effects model and was accompanied by substantial heterogeneity (I^2^ ≈ 77%). Therefore, the overall survival findings should be interpreted as a possible prognostic signal rather than as definitive evidence of an independent prognostic role.

For disease-free survival, the evidence was particularly weak. Only two studies were available, the effect estimates were unstable and diverged between fixed-effect and random-effects models, and heterogeneity was very high. Consequently, the DFS findings should be considered exploratory and should not be used to support firm conclusions regarding the prognostic value of miR-34a.

Trial sequential analysis did not provide conclusive evidence, as the accrued information size remained below the a priori information size. Overall, miR-34a remains a biologically plausible and promising candidate biomarker, but the certainty of the evidence is low. Larger prospective, site-specific studies with standardized assays, pre-defined thresholds, and appropriate adjustment for clinical covariates, including HPV/p16 status, are required before miR-34a can be considered for clinical prognostic stratification. If validated, miR-34a could be evaluated as part of multi-marker prognostic panels alongside established clinicopathological variables, using standardized analytical pipelines and externally validated thresholds to support translation into routine pathology workflows.

## Figures and Tables

**Table 1:** Data extracted from the 4 studies included, providing information regarding the type of tumor, the location of the tumor, the number of patients with data concerning the average age, the average or maximum follow-up, gender, and the common risk factors in the patients are reported to be smoking, alcohol, and HPV positivity; TNM (T: tumor size; N: regional lymph nodes; M: distant metastasis); TNM staging; cTNM, clinical TNM staging; N/A, not available; Ma (male); Fe (female); R (range); y (years); smoking (Sm); alcohol (Alc); SEM (standard error mean); RT (retrospective study); OPSCC (Oro pharyngeal squamous cell carcinoma); LSCC (larynx squamous cell carcinoma); \: data not present.

First Author, Date	Country	Study Design	Tumor Type/Tumor Site	miR	Follow-Up Months	Patient (Ma, Fe)	Age (Years)	Smoking	Alcohol	HPV	Staging
Shen et al., 2012 [22]	China	RT	69 LSCC	miR-34a	36	\	<60 y 33, ≥60 y 36	\	\	\	TNM staging: I–II 42, III–IV 27
Ogawa et al., 2012 [23]	Japan	RT	24 HNSCC (24 Sinonasal Squamous Cell Carcinomas)	miR-34a	53	16 Ma, 8 Fe	>60 y 14, <60 y 10	\	\	\	T stage T2 1, T3 10, T4a 13
Piotrowski et al., 2021 [24]	Poland	RT	79 HNSCC (37 OSCC, 9 OPSCC, 33 LSCC)	miR-146a, miR-449a, miR-126, miR-34a, miR-34b, miR-34c, miR-217, miR-378c, miR-6510, miR-96, miR-149, miR-133a,	~50	60 Ma, 19 Fe	OSCC 59.3 (±11.2), OPSCC 52.9 (±12.1), LSCC 62.2 (±12.5)	\	\	HPV-positive malignancies excluded	T stage OSCC T1–2 26, T3–4 11; LSCC T1–2 4, T3–4 28, NA 1; OPSCC T1-2 6, T3-4 3
Ren et al., 2023 [25]	China	RT	146 OSCC	miR-34a	~125	107 Ma, 39 Fe	≤60 y 80, <60 y 66	65 no, 81 yes	60 no, 86 yes	\	T stageT1 26, T2 65, T3 20, T4 35

**Table 2:** The values of HR (95% confidence interval) and RR for the different prognostic indices of survival are shown in the Table; overall survival (OS); diseases free survival (DFS); recurrence free survival (RFS); cancer specific survival (CSS); progression free survival (PFS); relative risk (RR); low versus high expression (L-H); high versus low expression (H-L).

First Author, Date	miR	Number, Tumor Type/Tumor Site	OS	DFS	CSS	RFS	PFS	RR
Shen et al., 2012 [22]	miR-34a	69 LSCC	/	4.02 (1.67–9.69) L-H	/	/	/	/
Ogawa et al., 2012 [23]	miR-34a	24 HNSCC (24 Sinonasal Squamous Cell Carcinomas)	/	0.005 (0.00–0.29) L-H p 0.011	0 not estimable			
Piotrowski et al., 2021 [24]	miR-34a	37 OSCC	6.452 (SE = 0.131), *p* = 0.028 L-H					
		33 LSCC	HR = 0.289 (SE = 2.171), *p* = 0.047 L-H					
		9 OPSCC	Kaplan Meier 0.479 (95% CI 0.035–6.566)					
Ren et al., 2023 [25]	miR-34a	146 OSCC	Kaplan Meier 2.707 (95% CI 1.713–4.278) L-H					

**Table 3:** Risk of bias assessment within the studies, with scores ranging from 8 to 10 indicating low quality, 11 to 14 indicating intermediate quality, and 15 to 18 indicating high quality.

First Author, Data	Sample	Clinical Data	Marker Quantification	Prognostication	Statistics	Classical Prognostic Factors	Score
Shen et al., 2012 [22]	3	2	3	2	1	1	12
Ogawa et al., 2012 [23]	3	3	3	3	2	3	17
Piotrowski et al., 2021 [24]	3	3	2	2	3	3	16
Ren et al., 2023 [25]	3	2	3	2	1	1	12

**Table 4:** Evaluation of GRADEpro GDT; CI, confidence interval; HR, hazard ratio. ⨁◯◯◯ Very low, ⨁⨁◯◯ Low.

Outcome	N° of Studies	Study Design	Risk of Bias	Inconsistency	Indirectness	Imprecision	Other considerations	N° of Patients	Relative (95% CI)	Absolute (95% CI)	Certainty
Overall survival (OS)	2	Retrospective cohorts	Serious	Serious	Not serious	Not serious	Publication bias suspected	225	HR 2.25 (1.48–3.41)	\	Low (⊕⊕◯◯)
Disease-free survival (DFS)	2	Retrospective cohorts	Serious ^a^	Very serious ^b^	Not serious	Very serious (wide CI; k = 2)	\	93	HR 0.19 (≈0.00–129.34)	\	Very low (⊕◯◯◯)

^a^ Downgraded by one level for serious risk of bias due to the retrospective design of the included prognostic-marker cohorts and possible residual confounding related to incomplete adjustment for established prognostic factors. ^b^ Downgraded by two levels for very serious inconsistency because the DFS analysis showed substantial heterogeneity (I^2^ = 91%) and markedly divergent effect estimates across studies.

## Data Availability

Data is contained within the article and Supplementary Material. All data generated or analyzed during this study are included in this published article.

## Supplementary Materials

The following supporting information can be downloaded at: https://www.mdpi.com/article/10.3390/ijms27114909/s1.
