# Supplementary material for: Prognostic Impact of miR-34a in Head and Neck Squamous Cell Carcinoma: A Systematic Review with Meta-Analysis and Trial Sequential Analysis
Source: Int J Mol Sci. 2026 May 29;27(11):4909. doi: 10.3390/ijms27114909 (PMC13256702; doi:10.3390/ijms27114909)
Supplement: Supplementary file 1 [file ijms-27-04909-s001.zip › cuttoff (1).pdf]

"cutoff value"	"p value"
71	0.913360257355866
72	0.87235059186773
73	0.879788667766904
74	0.757610830905121
75	0.906803614262958
76	0.756241675112793
77	0.77233365489055
78	0.630663537286964
79	0.686676103274574
80	0.624790778758775
81	0.625922166536621
82	0.440195193115637
83	0.316096856108225
84	0.147915344069203
85	0.194830452640969
86	0.446131585537735
87	0.284459367035367
88	0.264332800596584
89	0.126138335228096
90	0.0947473875474893
91	0.138114824099329
92	0.247016797465764
93	0.184202315007716
94	0.284006715856958
96	0.315711907982639
97	0.30286004044104
98	0.517073325203458
99	0.504308266963878
100	0.379181964028481
101	0.454493641808478
102	0.681117701594027
103	0.686166068102729
104	0.660307770359881
105	0.607681956392532
106	0.655975168258586
107	0.660972370829644
108	0.541167498031849
110	0.701941094201227
111	0.848431684152305
112	0.968066393884382
113	0.917545889244479
114	0.938351280618516
115	0.946208122204506
116	0.961029142231459
117	0.83914845673626
118	0.892017411225966
119	0.894227032624182
120	0.744428559307471
121	0.801146498811917
122	0.792929516129781
123	0.60257945600479
124	0.521066562189293
125	0.69934712953549
126	0.640815135215579
127	0.476598736566531
128	0.320197108437756
129	0.363651947315218
130	0.375947422737493
131	0.358214189632028
132	0.334132784399151
133	0.367317648534025
134	0.376615045293954

136 0.40776412454536
138 0.468140405987832
139 0.682364620796054
141 0.763720089197961
142 0.891539691407987
143 0.799122431671266
144 0.72187786605718
145 0.768198011092581
146 0.807962656333683
147 0.899292063791512
149 0.829568437902748
150 0.745365896468672
151 0.826352554469384
