# Supplementary material for: Prognostic Impact of miR-34a in Head and Neck Squamous Cell Carcinoma: A Systematic Review with Meta-Analysis and Trial Sequential Analysis
Source: Int J Mol Sci. 2026 May 29;27(11):4909. doi: 10.3390/ijms27114909 (PMC13256702; doi:10.3390/ijms27114909)
Supplement: Supplementary file 1 [file ijms-27-04909-s001.zip › KM2HR workflow/Piotrowski/KM_HR_report.pdf]

Kaplan–Meier → Hazard Ratio (Tierney method)

2025-10-30 08:24

Time axis: 0.0 – 55.0 | **Initial N:** N1=4, N2=5 | **Use NAR:** Yes

Result

HR (A vs B) = 0.479 (95% CI 0.035 – 6.566)

HR (B vs A) = 2.087 (95% CI 0.152 – 28.607)

logHR_AB = -0.7359, SE = 1.3356, O−E = -0.413, V = 0.561

Traced curves

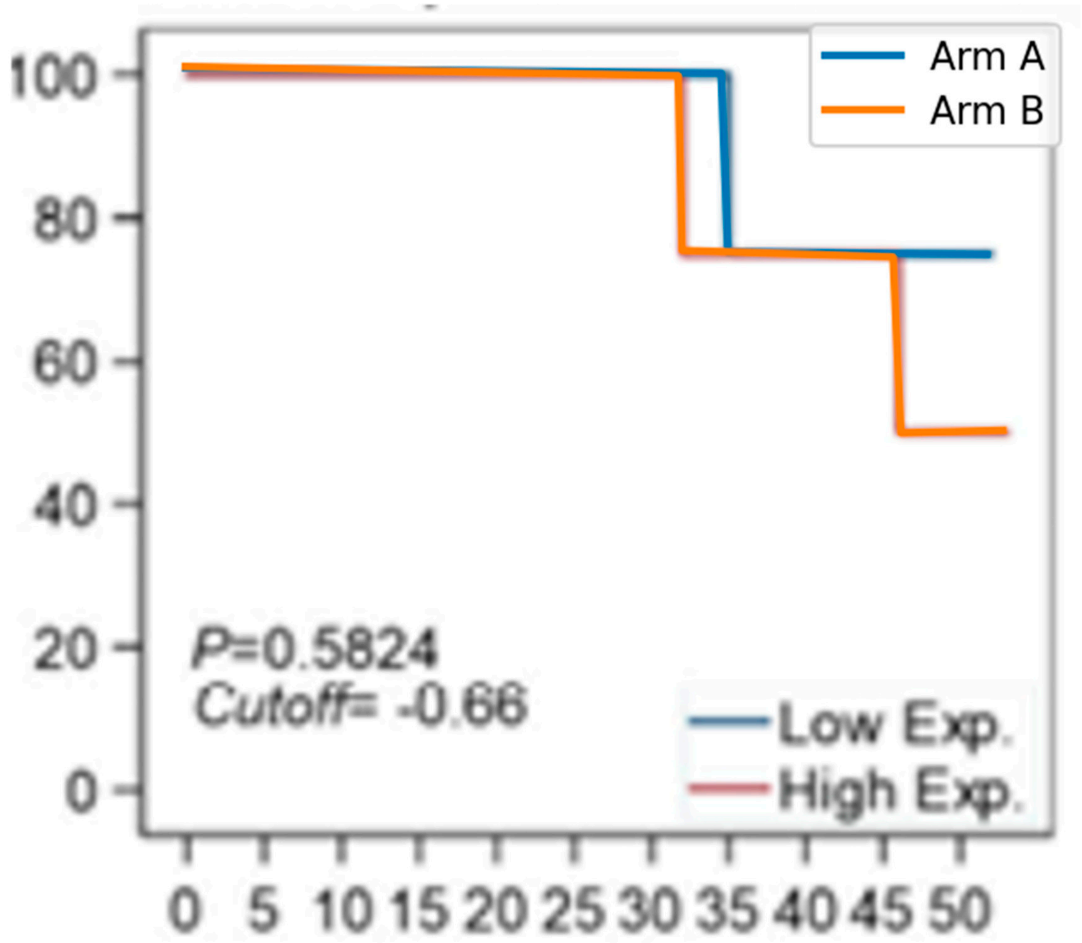

Numbers-at-Risk

time	arm1	arm2

0	4	5
5	4	4
10	4	4
15	4	4
20	4	4
25	4	4
30	4	3
35	3	3
40	2	3
45	2	2

Curve data (A & B)

t_A	S_A	t_B	S_B
2.68095	0.951424	2.68095	0.953099
35.096	0.944724	32.4963	0.941374
35.5022	0.721943	32.74	0.723618
51.1817	0.718593	45.4948	0.715243
		45.9823	0.495812
		52.1566	0.495812
