# Supplementary material for: Prognostic Impact of miR-34a in Head and Neck Squamous Cell Carcinoma: A Systematic Review with Meta-Analysis and Trial Sequential Analysis
Source: Int J Mol Sci. 2026 May 29;27(11):4909. doi: 10.3390/ijms27114909 (PMC13256702; doi:10.3390/ijms27114909)
Supplement: Supplementary file 1 [file ijms-27-04909-s001.zip › KM2HR workflow/ren/KM_HR_report.pdf]

Kaplan–Meier → Hazard Ratio (Tierney method)

2025-10-30 07:00

Time axis: 0.0 to 150.0 | **Initial N:** N1=88, N2=58 | **Use NAR:** Yes

Result

HR (A vs B) = 0.369 (95% CI 0.234 – 0.584)

HR (B vs A) = 2.707 (95% CI 1.713 – 4.278)

logHR_AB = -0.9958, SE = 0.2335, O−E = -18.267, V = 18.345

Traced curves

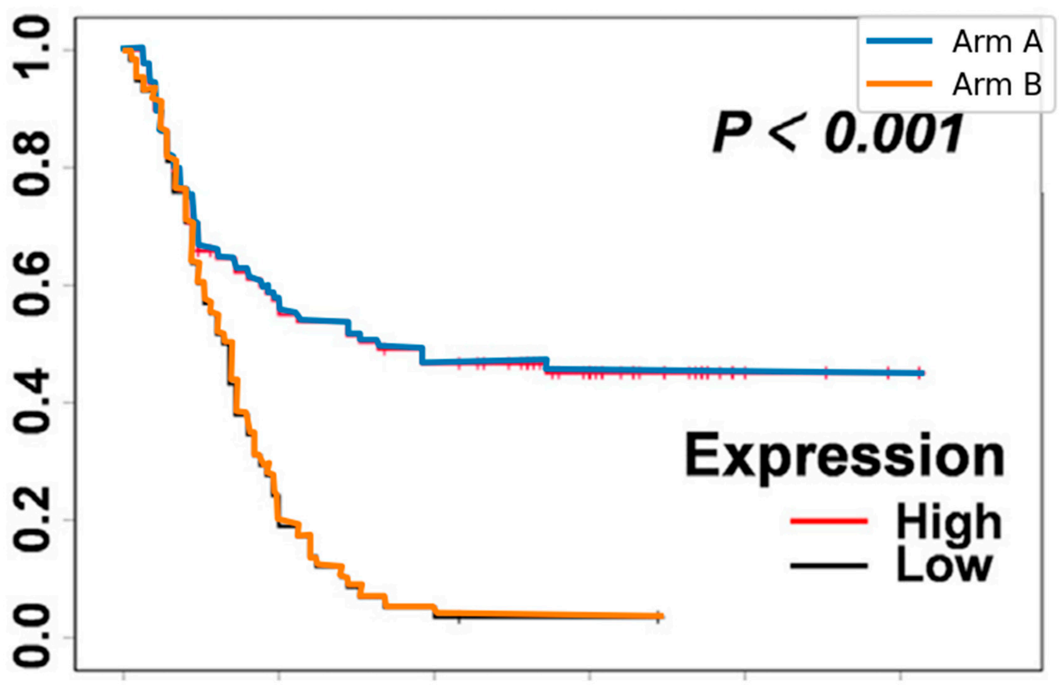

Numbers-at-Risk

time	arm1	arm2
0	88	58
25	63	33
50	38	8
75	13	0

100	0	0
125	0	0

Curve data (A & B)

t_A	S_A	t_B	S_B
7.54098	0.952998	7.86885	0.949757
10.4918	0.952998	8.68852	0.949757
10.6557	0.930308	8.85246	0.936791
11.4754	0.930308	9.5082	0.936791
11.6393	0.902755	9.5082	0.909238
12.459	0.901135	10.6557	0.909238
12.623	0.857374	10.6557	0.889789
13.1148	0.828201	11.9672	0.876823
13.4426	0.828201	12.459	0.876823
14.2623	0.824959	13.2787	0.831442
14.2623	0.792545	13.4426	0.831442
15.2459	0.787682	14.2623	0.82658
15.4098	0.769854	14.2623	0.787682
16.2295	0.769854	15.5738	0.73906
16.3934	0.73906	15.7377	0.73906
17.377	0.737439	17.2131	0.737439
17.541	0.726094	17.2131	0.690438
18.1967	0.726094	18.0328	0.627229
18.5246	0.687196	18.3607	0.627229
19.0164	0.685575	19.0164	0.594814
19.1803	0.65154	19.3443	0.594814

22.1311	0.645057	20	0.594814
22.2951	0.633712	20.1639	0.568882
24.5902	0.632091	20.9836	0.549433
25.082	0.615883	21.1475	0.549433
26.7213	0.615883	22.1311	0.518639
27.0492	0.602917	22.2951	0.518639
28.8525	0.596434	23.1148	0.515397
29.1803	0.58671	23.2787	0.502431
30	0.58671	24.2623	0.444084
30	0.578606	24.4262	0.444084
30.8197	0.578606	25.082	0.395462
30.9836	0.570502	25.2459	0.395462
31.6393	0.570502	26.5574	0.393841
31.8033	0.552674	27.2131	0.363047
34.2623	0.547812	27.8689	0.363047
35.082	0.536467	27.8689	0.329011
42.459	0.533225	28.5246	0.329011
42.459	0.515397	29.3443	0.312804
44.2623	0.515397	29.8361	0.301459
44.2623	0.505673	30.1639	0.301459
46.8852	0.505673	30.8197	0.298217
47.2131	0.495948	30.9836	0.272285
53.9344	0.492707	31.3115	0.267423
53.9344	0.470016	31.4754	0.230146
73.1148	0.460292	34.5902	0.204214
73.2787	0.460292	34.7541	0.204214

131.475	0.453809	36.5574	0.204214
		36.5574	0.170178
		37.377	0.170178
		37.7049	0.160454
		41.1475	0.144246
		41.4754	0.144246
		42.2951	0.139384
		42.459	0.12966
		44.2623	0.111831
		44.5902	0.111831
		48.0328	0.111831
		48.3607	0.095624
		55.5738	0.095624
		56.2295	0.0858995
		90.9836	0.0810373
