# Supplementary material for: Prognostic Impact of miR-34a in Head and Neck Squamous Cell Carcinoma: A Systematic Review with Meta-Analysis and Trial Sequential Analysis
Source: Int J Mol Sci. 2026 May 29;27(11):4909. doi: 10.3390/ijms27114909 (PMC13256702; doi:10.3390/ijms27114909)
Supplement: Supplementary file 1 [file ijms-27-04909-s001.zip › validation/KM2HR_validation_benchmark_summary.pdf]

Supplementary Table. KM2HR Validation and Benchmarking Summary

Internal formula check, HR direction harmonisation and external benchmarking of Kaplan-Meier-derived hazard ratios

Purpose

This supplementary table summarises the validation and benchmarking curves used to document the KM2HR workflow. The validation strategy included: (i) internal recalculation of logHR, standard error, hazard ratio and 95% confidence intervals from the exported O-E and V values; (ii) explicit harmonisation of HR direction; and (iii) comparison of reconstructed HRs with published or displayed HRs from Kaplan-Meier curves with available numbers-at-risk.

Abbreviations and acronyms

Abbreviation / term	Explanation
**ID**	Validation identifier assigned to each benchmark curve.
**KM**	Kaplan-Meier.
**KM2HR**	Kaplan-Meier to Hazard Ratio tool developed to reconstruct HRs from digitised Kaplan-Meier curves.
**HR**	Hazard ratio.
**95% CI**	95% confidence interval.
**OS**	Overall survival.
**DFS**	Disease-free survival.
**NAR**	Numbers-at-risk; number of patients still at risk at specified time points.
**TCGA**	The Cancer Genome Atlas.
**KM Plotter**	Public Kaplan-Meier Plotter database web tool.
**HNSCC**	Head and neck squamous cell carcinoma.
**HNC**	Head and neck cancer.
**OSCC**	Oral squamous cell carcinoma.
**LSCC**	Laryngeal squamous cell carcinoma.
**NPC**	Nasopharyngeal carcinoma.
**miR**	microRNA.
**hsa-miR**	Human mature microRNA nomenclature.
**Arm A / Arm B**	The two groups digitised in KM2HR. HR A vs B and HR B vs A are both exported to allow direction matching.
**Low / High**	Low-expression and high-expression groups, according to the original study or database cut-off.
**HR direction**	Direction in which the published or displayed HR was reported, e.g. high vs low or low vs high.
**Compared KM2HR HR**	The reconstructed KM2HR HR selected for comparison after matching the published/displayed HR direction.
**Agreement**	Approximate relative difference between the compared KM2HR HR and the published/displayed HR.
**Published-study validation**	Benchmark based on curves from primary published studies.
**External HNSCC/HNC benchmark**	Additional head-and-neck miRNA benchmark curve used for technical validation.
**TCGA/KM Plotter benchmark**	Database-derived technical benchmark from TCGA HNSCC curves generated using KM Plotter; not included in the pooled meta-analysis.

Agreement categories

Category	Approximate relative difference
**Excellent**	<5%
**Good**	5-10%
**Acceptable**	10-20%
**Moderate discrepancy**	20-30%, usually interpreted with caution when small or unbalanced risk sets are present
**Unstable/stress-test**	>30% or highly unstable curves with few events or long tails

Validation and benchmarking summary

Note: The compared KM2HR HR was selected after matching the published/displayed HR direction. For example, when Arm A was low expression and Arm B was high expression, a displayed HR reported as high vs low was compared with KM2HR HR B vs A.

ID	Study / dataset	miRNA / curve	Endpoint	Cancer type / dataset	Arm A	Arm B	Published / displayed HR	HR direction	Compared KM2HR HR	Agreement	Category
**V1**	Piotrowski et al., 2021	Oral miR-34a-5p	OS	Oral cancer / OSCC	Low	High	0.155	High vs Low	0.124	~20.0%	Published-study validation - acceptable/borderline
**V2**	Piotrowski et al., 2021	Oral miR-96-5p	OS	Oral cancer / OSCC	Low	High	0.225	High vs Low	0.240	~6.7%	Published-study validation - good
**V3**	Piotrowski et al., 2021	Oral miR-133a-5p	OS	Oral cancer / OSCC	Low	High	0.153	High vs Low	0.112	~26.8%	Published-study validation - moderate discrepancy
**V4**	Piotrowski et al., 2021	Larynx miR-146a-5p	OS	Laryngeal cancer / LSCC	Low	High	9.671	High vs Low	9.741	~0.7%	Published-study validation - excellent
**V5**	de Jong et al., 2015	miR-203	Local recurrence-free probability	Laryngeal cancer / HNSCC	High	Low	0.364	High vs Low	0.355	~2.5%	External HNSCC benchmark - excellent
**V6**	Liu et al., 2013	miR-451	OS	NPC / head-and-neck	High	Low	1.98	Low vs High	2.004	~1.2%	External HNC benchmark - excellent
**V7**	Liu et al., 2013	miR-451	DFS	NPC / head-and-neck	High	Low	1.68	Low vs High	1.730	~3.0%	External HNC benchmark - excellent
**V8**	TCGA/KM Plotter	hsa-miR-21	OS	HNSCC / TCGA-derived	Low	High	1.23	High vs Low	1.238	~0.7%	TCGA/KM Plotter benchmark - excellent
**V9**	TCGA/KM Plotter	hsa-miR-155	OS	HNSCC / TCGA-derived	Low	High	0.73	High vs Low	0.656	~10.1%	TCGA/KM Plotter benchmark - acceptable
**V10**	TCGA/KM Plotter	hsa-miR-31	OS, 60 months	HNSCC / TCGA-derived	Low	High	1.27	High vs Low	1.221	~3.9%	TCGA/KM Plotter benchmark - excellent
**V11**	TCGA/KM Plotter	hsa-miR-197	OS, 60 months	HNSCC / TCGA-derived	Low	High	1.48	High vs Low	1.443	~2.5%	TCGA/KM Plotter benchmark - excellent
**V12**	TCGA/KM Plotter	hsa-miR-100	OS, 60 months	HNSCC / TCGA-derived	Low	High	1.93	High vs Low	1.814	~6.0%	TCGA/KM Plotter benchmark - good

Interpretative note

The TCGA/KM Plotter curves were used as database-derived technical benchmarks because the displayed HRs, confidence intervals and numbers-at-risk could be directly compared with the reconstructed HRs. These curves were classified separately from published-study validation curves and were not used to derive pooled clinical estimates in the meta-analysis.

The validation should therefore be interpreted as a technical check of the KM2HR workflow, including formula implementation, HR direction harmonisation, risk-set handling and agreement with external HR benchmarks.
