# Supplementary material for: Prognostic Impact of miR-34a in Head and Neck Squamous Cell Carcinoma: A Systematic Review with Meta-Analysis and Trial Sequential Analysis
Source: Int J Mol Sci. 2026 May 29;27(11):4909. doi: 10.3390/ijms27114909 (PMC13256702; doi:10.3390/ijms27114909)
Supplement: Supplementary file 1 [file ijms-27-04909-s001.zip › validation/Set 1 — Published-paper validation/mir 133a oral OS Piotrowski et al.,/KM2HR_report.pdf]

KM2HR — Kaplan–Meier → Hazard Ratio (Tierney method)

2026-05-09 11:43

Author: Dioguardi Mario — Università di Foggia

Time axis: 0.0 – 50.0 | **Initial N:** N1=6, N2=31 | **Use NAR:** Yes

Result

HR (A vs B) = 8.960 (95% CI 1.137 – 70.616)

HR (B vs A) = 0.112 (95% CI 0.014 – 0.880)

logHR_AB = 2.1927, SE = 1.0533, O−E = 1.976, V = 0.901

Traced curves

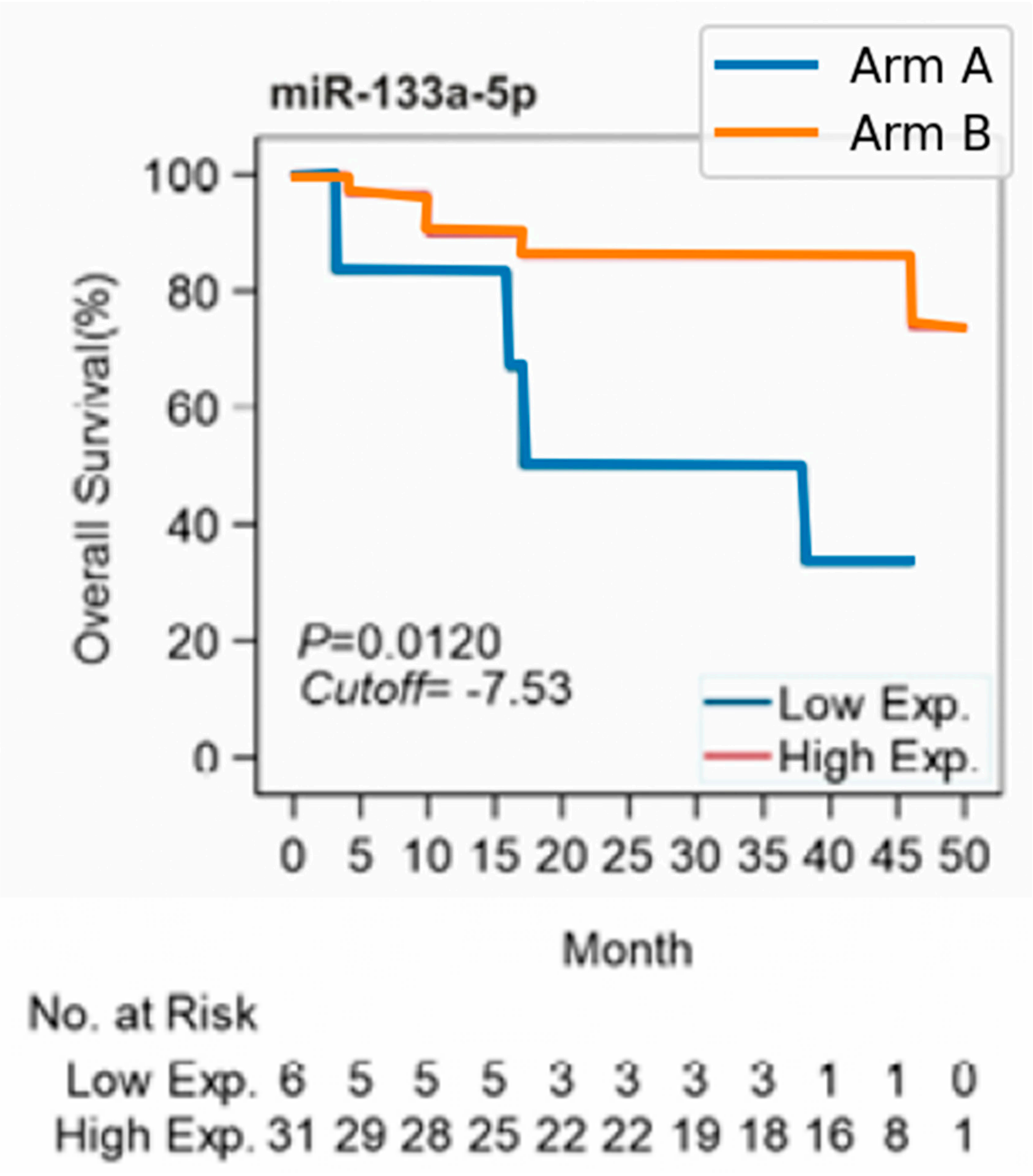

Numbers-at-Risk

time	arm1	arm2
0	6	31
5	5	29
10	5	28

15	5	25
20	3	22
25	3	22
30	3	19
35	1	18
40	1	16
45	1	8
50	0	1

Curve data (A & B)

t_A	S_A	t_B	S_B
0.142045	0.993846	0.142045	0.990769
3.125	0.993846	4.11932	0.990769
3.26705	0.843077	4.11932	0.969231
15.767	0.84	9.80114	0.907692
16.0511	0.689231	9.94318	0.907692
17.0455	0.689231	16.9034	0.867692
17.3295	0.529231	17.0455	0.867692
37.7841	0.526154	45.8807	0.864615
38.2102	0.372308	46.0227	0.756923
45.8807	0.372308	49.7159	0.747692
