# Supplementary material for: Prognostic Impact of miR-34a in Head and Neck Squamous Cell Carcinoma: A Systematic Review with Meta-Analysis and Trial Sequential Analysis
Source: Int J Mol Sci. 2026 May 29;27(11):4909. doi: 10.3390/ijms27114909 (PMC13256702; doi:10.3390/ijms27114909)
Supplement: Supplementary file 1 [file ijms-27-04909-s001.zip › validation/Set 1 — Published-paper validation/mir 146a larinx OS Piotrowski et al.,/KM2HR_report.pdf]

KM2HR — Kaplan–Meier → Hazard Ratio (Tierney method)

2026-05-09 11:19

Author: Dioguardi Mario — Università di Foggia

Time axis: 0.0 – 50.0 | **Initial N:** N1=28, N2=5 | **Use NAR:** Yes

Result

HR (A vs B) = 0.103 (95% CI 0.015 – 0.698)

HR (B vs A) = 9.741 (95% CI 1.434 – 66.190)

logHR_AB = -2.2763, SE = 0.9776, O−E = -2.382, V = 1.046

Traced curves

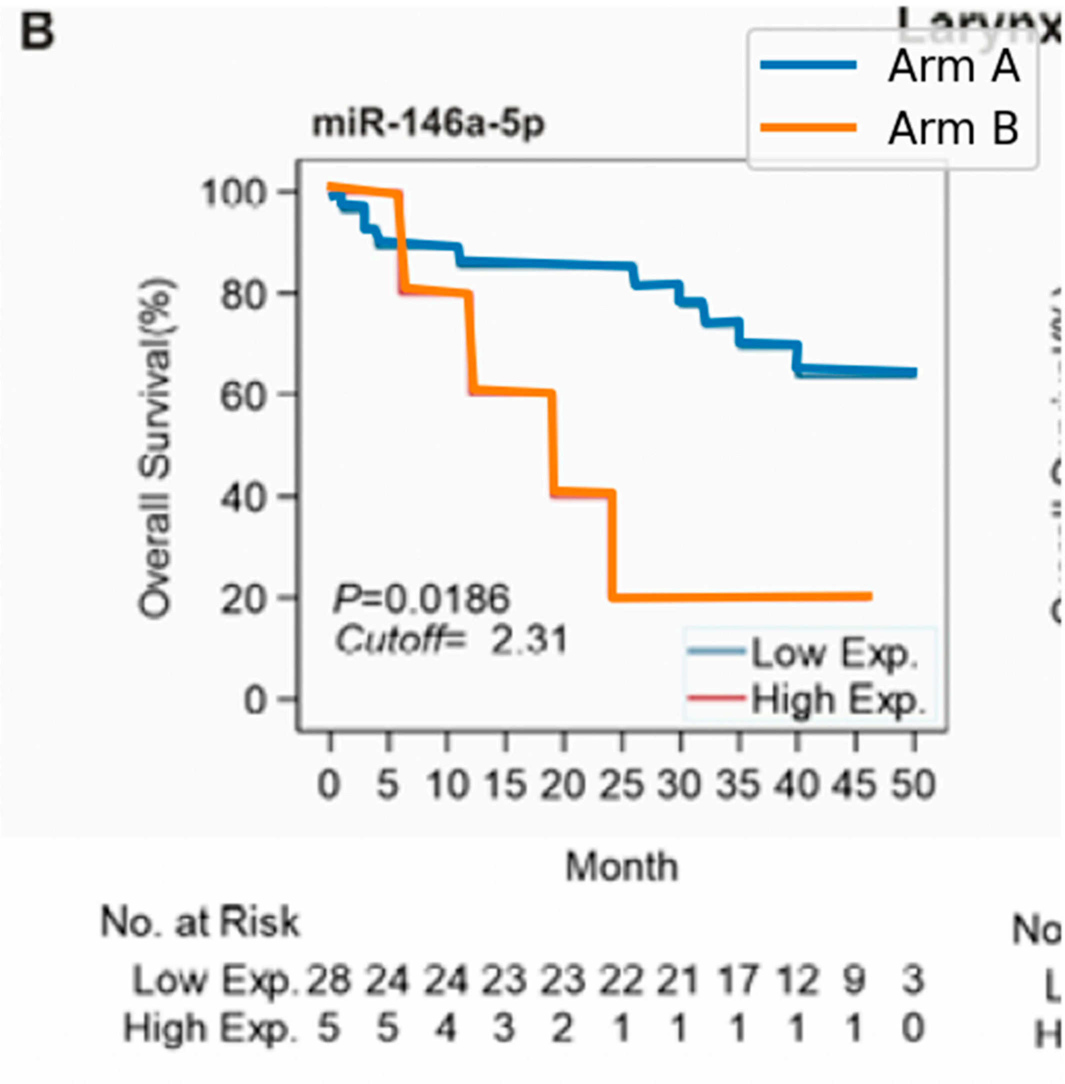

Numbers-at-Risk

time	arm1	arm2
0	28	5
5	24	5
10	24	4
15	23	3
20	23	2

25	22	1
30	21	1
35	17	1
40	12	1
45	9	1
50	3	0

Curve data (A & B)

t_A	S_A	t_B	S_B
0.33557	0.992727	0.167785	1
1.00671	0.992727	5.87248	0.996364
1.00671	0.978182	6.54362	0.821818
3.02013	0.974545	11.9128	0.810909
3.02013	0.930909	12.4161	0.632727
3.85906	0.930909	19.1275	0.625455
4.36242	0.909091	19.2953	0.443636
11.0738	0.898182	24.3289	0.44
11.2416	0.872727	24.3289	0.243636
26.0067	0.861818	46.3087	0.243636
26.3423	0.825455		
30.0336	0.825455		
30.0336	0.796364		
32.047	0.796364		
32.3826	0.756364		
35.2349	0.756364		
35.2349	0.72		

40.1007	0.672727
40.2685	0.672727
50.1678	0.665455
