# Supplementary material for: Prognostic Impact of miR-34a in Head and Neck Squamous Cell Carcinoma: A Systematic Review with Meta-Analysis and Trial Sequential Analysis
Source: Int J Mol Sci. 2026 May 29;27(11):4909. doi: 10.3390/ijms27114909 (PMC13256702; doi:10.3390/ijms27114909)
Supplement: Supplementary file 1 [file ijms-27-04909-s001.zip › validation/Set 1 — Published-paper validation/mir 203 larinx LR de Jong et al.,/KM2HR_report.pdf]

KM2HR — Kaplan–Meier → Hazard Ratio (Tierney method)

2026-05-11 06:20

Author: Dioguardi Mario — Università di Foggia

Time axis: 0.0 – 5.0 | **Initial N:** N1=17, N2=17 | **Use NAR:** Yes

Result

HR (A vs B) = 0.355 (95% CI 0.133 – 0.944)

HR (B vs A) = 2.820 (95% CI 1.060 – 7.505)

logHR_AB = -1.0368, SE = 0.4994, O−E = -4.158, V = 4.010

Traced curves

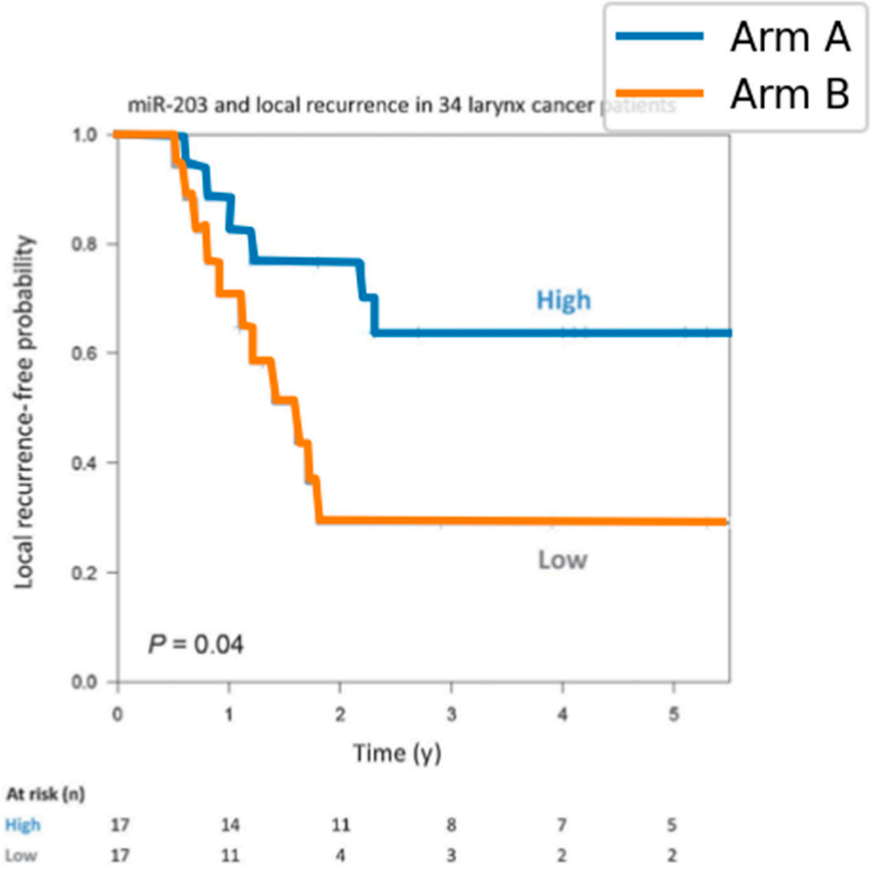

Numbers-at-Risk

time	arm1	arm2
0	17	17
1	14	11
2	11	4
3	8	3
4	7	2
5	5	2

Curve data (A & B)

t_A	S_A	t_B	S_B
0.0159744	0.996403	0.0159744	0.996403
0.559105	0.992806	0.479233	0.996403
0.57508	0.946043	0.495208	0.94964
0.734824	0.935252	0.543131	0.942446
0.750799	0.884892	0.57508	0.888489
0.926518	0.823741	0.623003	0.888489
0.942492	0.823741	0.654952	0.823741
1.10224	0.820144	0.734824	0.823741
1.13419	0.766187	0.750799	0.766187
1.98083	0.76259	0.846645	0.76259
2.01278	0.697842	0.846645	0.705036
2.10863	0.697842	1.02236	0.705036
2.10863	0.633094	1.03834	0.647482
4.98403	0.633094	1.11821	0.643885
		1.11821	0.582734
		1.26198	0.582734

1.3099	0.510791
1.45367	0.510791
1.5016	0.431655
1.5655	0.431655
1.58147	0.366906
1.62939	0.366906
1.66134	0.291367
4.9361	0.28777
