# Supplementary material for: Prognostic Impact of miR-34a in Head and Neck Squamous Cell Carcinoma: A Systematic Review with Meta-Analysis and Trial Sequential Analysis
Source: Int J Mol Sci. 2026 May 29;27(11):4909. doi: 10.3390/ijms27114909 (PMC13256702; doi:10.3390/ijms27114909)
Supplement: Supplementary file 1 [file ijms-27-04909-s001.zip › validation/Set 1 — Published-paper validation/mir 34a oral OS Piotrowski et al.,/KM2HR_report.pdf]

KM2HR — Kaplan–Meier → Hazard Ratio (Tierney method)

2026-05-09 10:04

Author: Dioguardi Mario — Università di Foggia

Time axis: 0.0 – 50.0 | **Initial N:** N1=11, N2=26 | **Use NAR:** Yes

Result

HR (A vs B) = 8.051 (95% CI 1.490 – 43.496)

HR (B vs A) = 0.124 (95% CI 0.023 – 0.671)

logHR_AB = 2.0858, SE = 0.8607, O−E = 2.816, V = 1.350

Traced curves

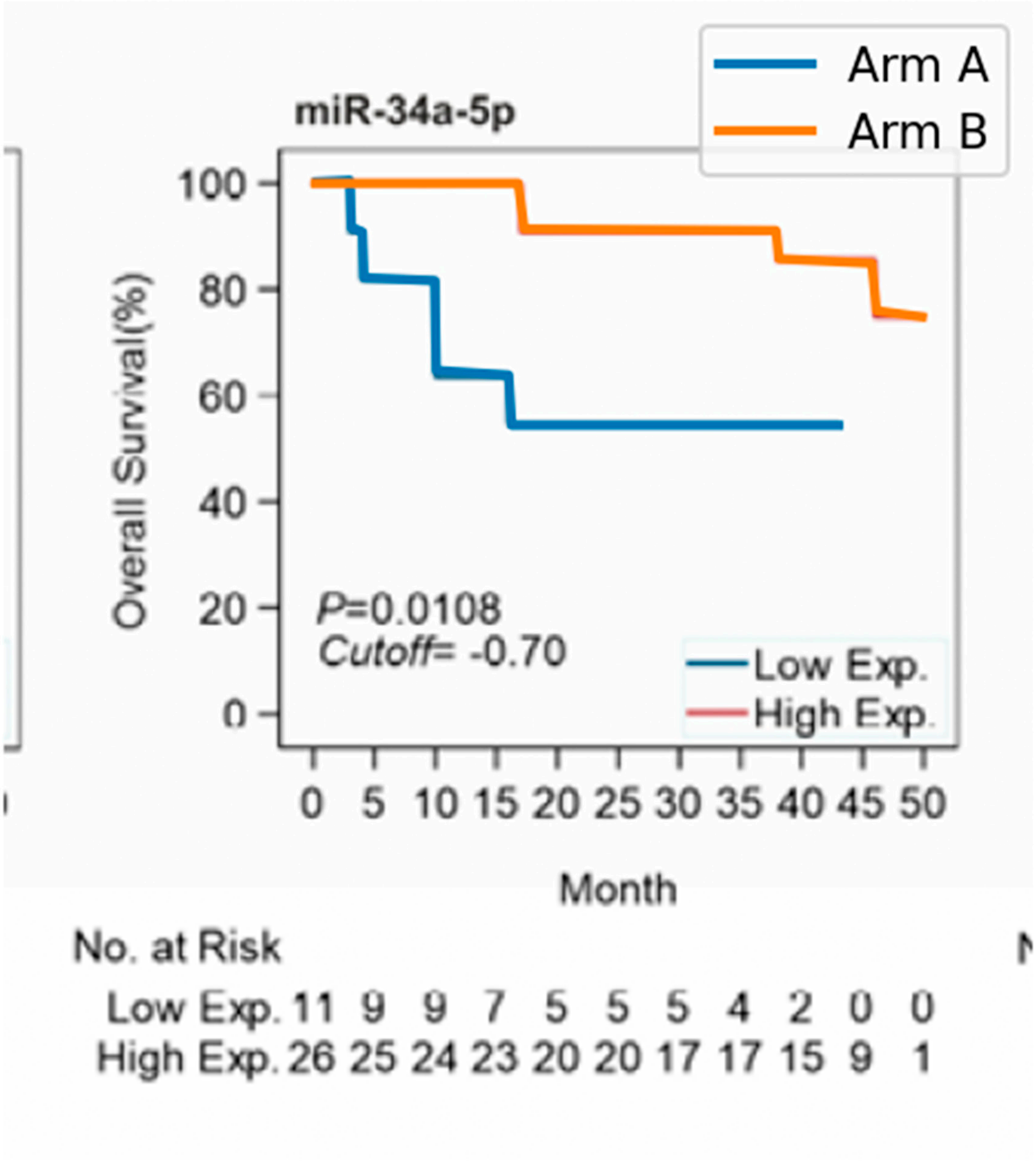

Numbers-at-Risk

time	arm1	arm2
0	11	26
5	9	25
10	9	24

15	7	23
20	5	20
25	5	20
30	5	17
35	4	17
40	2	15
45	0	9
50	0	1

Curve data (A & B)

t_A	S_A	t_B	S_B
0.385604	0.99723	0.257069	0.99446
3.08483	0.99723	16.838	0.99446
3.21337	0.916898	17.3522	0.914127
4.11311	0.908587	37.9177	0.911357
4.24165	0.828255	38.1748	0.861496
10.0257	0.822715	45.7584	0.853186
10.1542	0.66482	46.144	0.770083
16.0668	0.65651	49.8715	0.759003
16.3239	0.567867		
43.0591	0.567867		
