# Supplementary material for: Prognostic Impact of miR-34a in Head and Neck Squamous Cell Carcinoma: A Systematic Review with Meta-Analysis and Trial Sequential Analysis
Source: Int J Mol Sci. 2026 May 29;27(11):4909. doi: 10.3390/ijms27114909 (PMC13256702; doi:10.3390/ijms27114909)
Supplement: Supplementary file 1 [file ijms-27-04909-s001.zip › validation/Set 1 — Published-paper validation/mir 451 NPC DFS Liu et al.,/KM2HR_report.pdf]

KM2HR — Kaplan–Meier → Hazard Ratio (Tierney method)

2026-05-11 06:52

Author: Dioguardi Mario — Università di Foggia

Time axis: 0.0 – 96.0 | **Initial N:** N1=140, N2=140 | **Use NAR:** Yes

Result

HR (A vs B) = 0.578 (95% CI 0.370 – 0.903)

HR (B vs A) = 1.730 (95% CI 1.108 – 2.702)

logHR_AB = -0.5482, SE = 0.2275, O−E = -10.591, V = 19.321

Traced curves

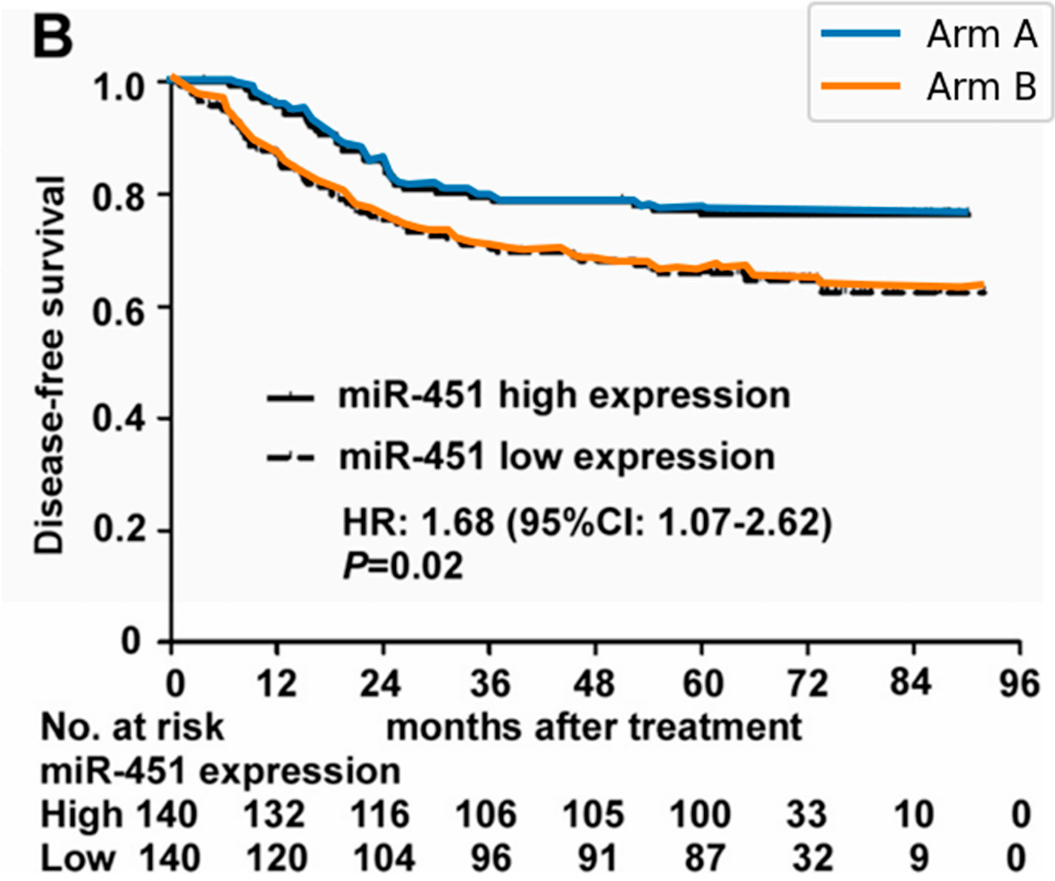

Numbers-at-Risk

time	arm1	arm2
0	140	140
12	132	120
24	116	104
36	106	96
48	105	91
60	100	87
72	33	32
84	10	9
96	0	0

Curve data (A & B)

t_A	S_A	t_B	S_B
-0.223256	0.996466	0.223256	1
6.69767	0.996466	2.90233	0.971731
7.14419	0.992933	5.80465	0.964664
9.15349	0.985866	6.25116	0.943463
9.37674	0.975265	7.5907	0.918728
11.8326	0.954064	9.15349	0.890459
12.7256	0.954064	12.0558	0.865724
13.6186	0.943463	12.7256	0.85159
14.9581	0.943463	14.5116	0.833922
15.8512	0.925795	16.5209	0.816254
18.0837	0.90106	19.4233	0.798587
18.7535	0.890459	20.7628	0.773852
19.4233	0.883392	22.5488	0.766784

21.4326	0.876325	24.7814	0.749117
22.3256	0.85159	26.3442	0.738516
23.8884	0.85159	27.907	0.731449
24.7814	0.823322	29.0233	0.727915
25.6744	0.812721	31.2558	0.727915
26.7907	0.809187	32.1488	0.713781
29.693	0.809187	33.7116	0.706714
30.8093	0.80212	36.8372	0.699647
33.4884	0.80212	37.9535	0.696113
34.6047	0.791519	39.7395	0.69258
35.9442	0.791519	43.9814	0.69258
37.0605	0.780919	45.5442	0.681979
52.2419	0.780919	46.214	0.678445
53.1349	0.770318	47.5535	0.678445
54.0279	0.770318	50.2326	0.671378
55.1442	0.766784	52.9116	0.671378
60.0558	0.766784	53.8047	0.671378
60.5023	0.766784	55.1442	0.657244
89.7488	0.759717	57.1535	0.657244
		59.386	0.657244
		61.6186	0.657244
		62.2884	0.657244
		64.9674	0.657244
		65.8605	0.646643
		71.8884	0.64311
		73.0047	0.64311

73.4512	0.632509
79.4791	0.628975
89.3023	0.625442
91.5349	0.625442
