# Supplementary material for: Prognostic Impact of miR-34a in Head and Neck Squamous Cell Carcinoma: A Systematic Review with Meta-Analysis and Trial Sequential Analysis
Source: Int J Mol Sci. 2026 May 29;27(11):4909. doi: 10.3390/ijms27114909 (PMC13256702; doi:10.3390/ijms27114909)
Supplement: Supplementary file 1 [file ijms-27-04909-s001.zip › validation/Set 1 — Published-paper validation/mir 451 NPC OS Liu et al.,/KM2HR_report.pdf]

KM2HR — Kaplan–Meier → Hazard Ratio (Tierney method)

2026-05-11 06:39

Author: Dioguardi Mario — Università di Foggia

Time axis: 0.0 – 96.0 | **Initial N:** N1=140, N2=140 | **Use NAR:** Yes

Result

HR (A vs B) = 0.499 (95% CI 0.300 – 0.830)

HR (B vs A) = 2.004 (95% CI 1.205 – 3.333)

logHR_AB = -0.6953, SE = 0.2595, O–E = -10.327, V = 14.852

Traced curves

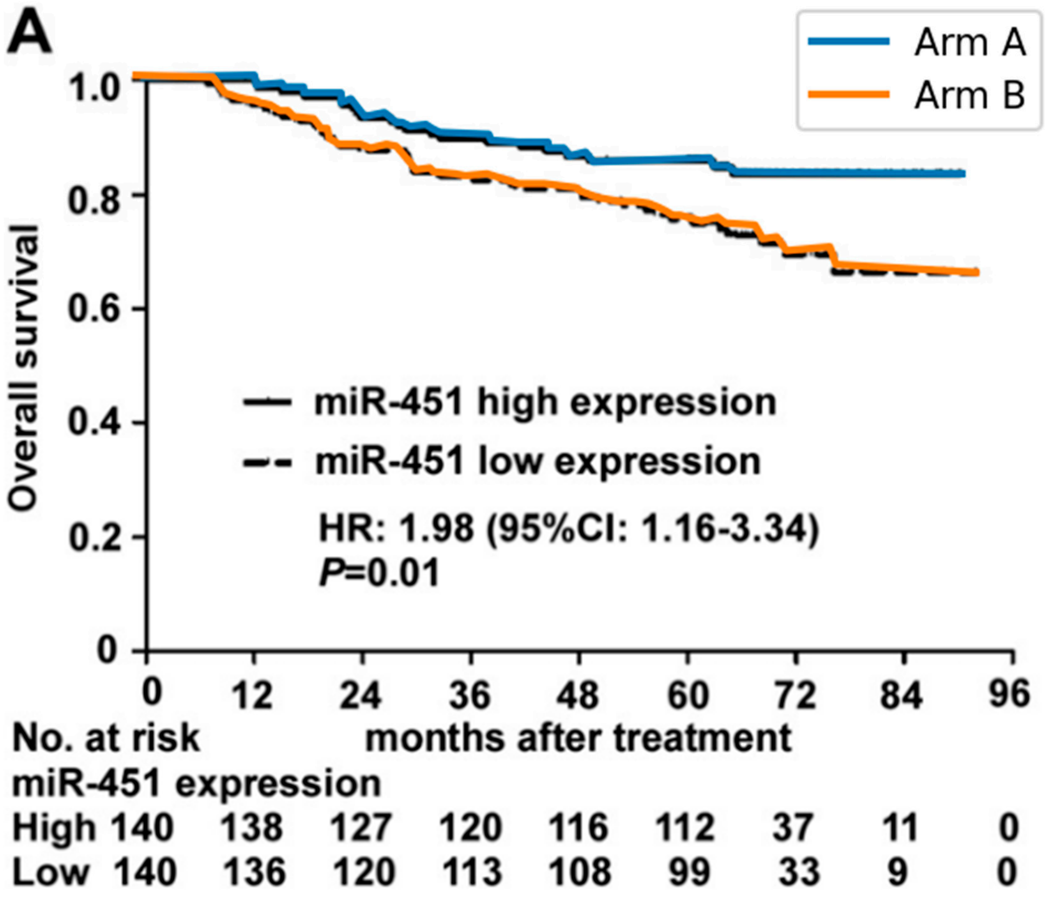

Numbers-at-Risk

time	arm1	arm2
0	140	140
12	138	136
24	127	120
36	120	113
48	116	108
60	112	99
72	37	33
84	11	9
96	0	0

Curve data (A & B)

t_A	S_A	t_B	S_B
-0.218679	1	-1.09339	1
12.0273	1	7.43508	1
12.246	0.989619	8.09112	0.989619
15.0888	0.989619	8.74715	0.975779
15.3075	0.986159	10.0592	0.968858
17.4943	0.986159	10.9339	0.965398
17.4943	0.975779	12.246	0.961938
21.6492	0.975779	12.6834	0.958478
21.6492	0.958478	13.7768	0.955017
22.7426	0.958478	14.8702	0.944637
24.2733	0.934256	15.7449	0.944637
26.4601	0.934256	16.4009	0.934256
27.7722	0.923875	18.5877	0.930796

28.4282	0.923875	19.4624	0.913495
29.303	0.916955	20.1185	0.913495
31.0524	0.916955	20.3371	0.896194
31.7084	0.913495	21.4305	0.885813
32.5831	0.906574	23.836	0.885813
38.0501	0.903114	24.9294	0.878893
38.2688	0.892734	26.6788	0.878893
41.3303	0.889273	27.7722	0.878893
41.7677	0.889273	29.0843	0.858131
44.6105	0.889273	29.959	0.84083
44.6105	0.878893	31.4897	0.84083
46.3599	0.878893	31.9271	0.83737
47.0159	0.865052	34.3326	0.83391
48.7654	0.865052	35.426	0.83045
49.6401	0.854671	37.8314	0.83045
62.5421	0.854671	41.3303	0.816609
62.9795	0.847751	44.1731	0.816609
64.5103	0.847751	47.672	0.809689
65.385	0.83737	48.328	0.802768
90.533	0.83391	50.0774	0.792388
		52.4829	0.785467
		54.2323	0.785467
		55.7631	0.782007
		56.8565	0.775087
		57.7312	0.768166
		58.3872	0.761246

59.262	0.761246
61.6674	0.750865
63.4169	0.750865
64.2916	0.747405
67.5718	0.743945
68.4465	0.719723
69.9772	0.719723
71.0706	0.698962
75.8815	0.698962
76.5376	0.67474
92.0638	0.6609
