# Supplementary material for: Prognostic Impact of miR-34a in Head and Neck Squamous Cell Carcinoma: A Systematic Review with Meta-Analysis and Trial Sequential Analysis
Source: Int J Mol Sci. 2026 May 29;27(11):4909. doi: 10.3390/ijms27114909 (PMC13256702; doi:10.3390/ijms27114909)
Supplement: Supplementary file 1 [file ijms-27-04909-s001.zip › validation/Set 1 — Published-paper validation/mir 96 oral OS Piotrowski et al.,/KM2HR_report.pdf]

KM2HR — Kaplan–Meier → Hazard Ratio (Tierney method)

2026-05-09 10:46

Author: Dioguardi Mario — Università di Foggia

Time axis: 0.0 – 50.0 | **Initial N:** N1=15, N2=22 | **Use NAR:** Yes

Result

HR (A vs B) = 4.173 (95% CI 0.992 – 17.554)

HR (B vs A) = 0.240 (95% CI 0.057 – 1.008)

logHR_AB = 1.4287, SE = 0.7330, O−E = 2.659, V = 1.861

Traced curves

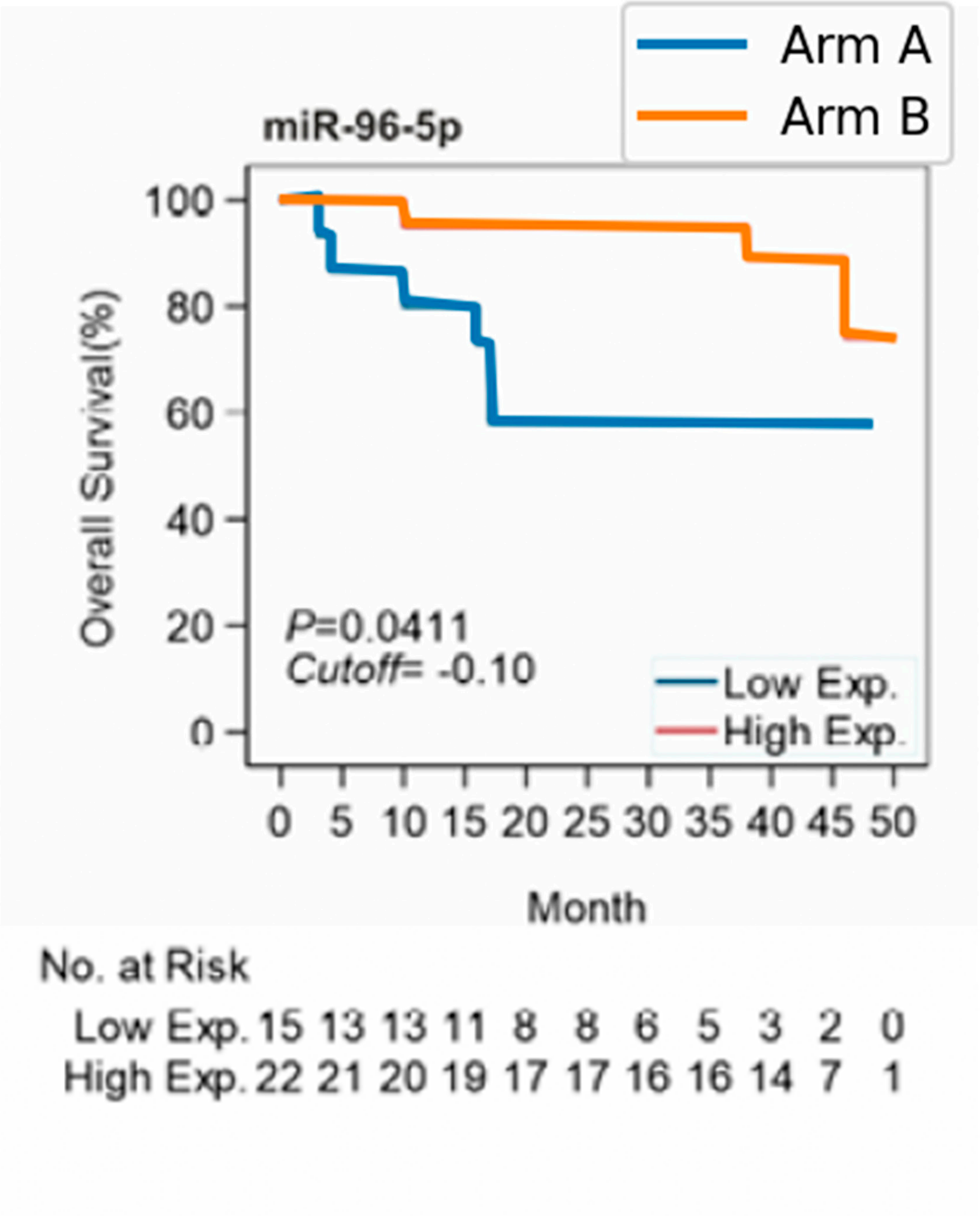

Numbers-at-Risk

time	arm1	arm2
0	15	22

5	13	21
10	13	20
15	11	19
20	8	17
25	8	17
30	6	16
35	5	16
40	3	14
45	2	7
50	0	1

Curve data (A & B)

t_A	S_A	t_B	S_B
0.438596	0.996825	0.438596	0.996825
3.21637	0.996825	9.94152	0.993651
3.21637	0.942857	10.3801	0.955556
4.23977	0.933333	37.8655	0.946032
4.23977	0.87619	38.0117	0.895238
9.94152	0.869841	45.9064	0.888889
10.2339	0.819048	45.9064	0.761905
15.9357	0.806349	49.7076	0.752381
15.9357	0.749206		
17.1053	0.742857		
17.3977	0.606349		
47.807	0.6		
