# Supplementary material for: Prognostic Impact of miR-34a in Head and Neck Squamous Cell Carcinoma: A Systematic Review with Meta-Analysis and Trial Sequential Analysis
Source: Int J Mol Sci. 2026 May 29;27(11):4909. doi: 10.3390/ijms27114909 (PMC13256702; doi:10.3390/ijms27114909)
Supplement: Supplementary file 1 [file ijms-27-04909-s001.zip › validation/Set 2 — TCGAKM Plotter database-derived validation/TCGA mir 155 HNSCC/KM2HR_report.pdf]

KM2HR — Kaplan–Meier → Hazard Ratio (Tierney method)

2026-05-11 07:53

Author: Dioguardi Mario — Università di Foggia

Time axis: 0.0 – 120.0 | **Initial N:** N1=130, N2=392 | **Use NAR:** Yes

Result

HR (A vs B) = 1.524 (95% CI 1.108 – 2.096)

HR (B vs A) = 0.656 (95% CI 0.477 – 0.903)

logHR_AB = 0.4212, SE = 0.1626, O−E = 15.924, V = 37.809

Traced curves

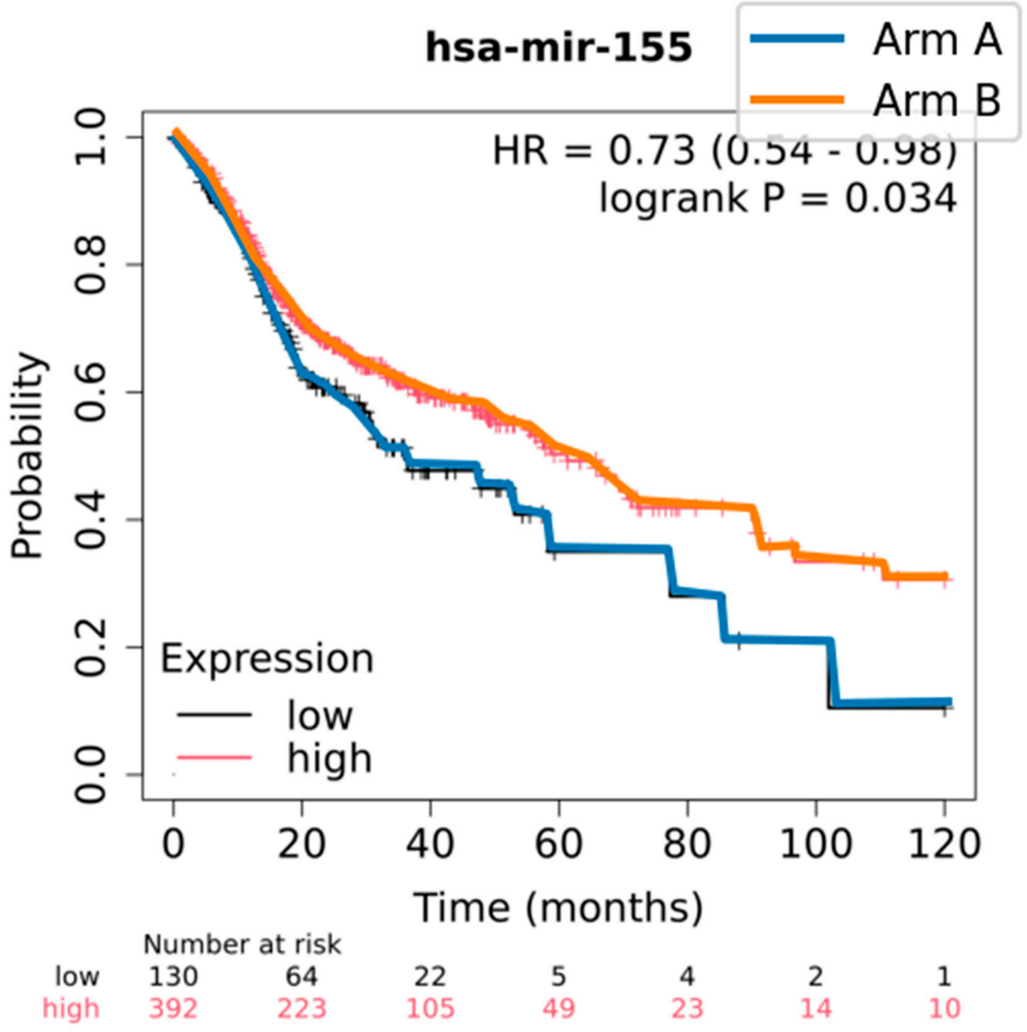

Numbers-at-Risk

time	arm1	arm2
0	130	392
20	64	223
40	22	105
60	5	49
80	4	23

100	2	14
120	1	10

Curve data (A & B)

t_A	S_A	t_B	S_B
0.604534	0.990826	0.906801	1
3.6272	0.948012	6.04534	0.93578
6.34761	0.908257	9.67254	0.868502
8.76574	0.868502	12.9975	0.804281
12.3929	0.801223	17.8338	0.743119
13.602	0.770642	21.461	0.69419
16.927	0.69419	28.1108	0.651376
20.2519	0.623853	32.3426	0.629969
21.7632	0.617737	36.5743	0.611621
23.8791	0.605505	43.2242	0.584098
25.3904	0.590214	48.3627	0.577982
28.1108	0.571865	51.3854	0.553517
31.1335	0.535168	55.6171	0.541284
33.2494	0.507645	59.2443	0.510703
35.9698	0.507645	64.3829	0.492355
36.8766	0.48318	69.2191	0.449541
47.1537	0.480122	72.2418	0.425076
47.7582	0.452599	89.7733	0.412844
52.2922	0.449541	90.3778	0.394495
53.199	0.412844	91.2846	0.351682
58.0353	0.40367	96.4232	0.351682

58.6398	0.351682	96.4232	0.33945
76.7758	0.348624	110.025	0.327217
77.6826	0.284404	110.63	0.30581
84.937	0.275229	119.395	0.30581
85.5416	0.207951		
101.864	0.204893		
102.771	0.107034		
120	0.107034		
