# Supplementary material for: Prognostic Impact of miR-34a in Head and Neck Squamous Cell Carcinoma: A Systematic Review with Meta-Analysis and Trial Sequential Analysis
Source: Int J Mol Sci. 2026 May 29;27(11):4909. doi: 10.3390/ijms27114909 (PMC13256702; doi:10.3390/ijms27114909)
Supplement: Supplementary file 1 [file ijms-27-04909-s001.zip › validation/Set 2 — TCGAKM Plotter database-derived validation/TCGA mir 21 HNSCC/KM2HR_report.pdf]

## KM2HR — Kaplan–Meier → Hazard Ratio (Tierney method)

2026-05-11 07:17

Author: Dioguardi Mario — Università di Foggia

**Time axis:** 0.0 – 120.0 | **Initial N:** N1=235, N2=287 | **Use NAR:** Yes

### Result

HR (A vs B) = 0.808 (95% CI 0.616 – 1.059)

HR (B vs A) = 1.238 (95% CI 0.944 – 1.624)

logHR_AB = -0.2136, SE = 0.1383, O−E = -11.163, V = 52.255

## Traced curves

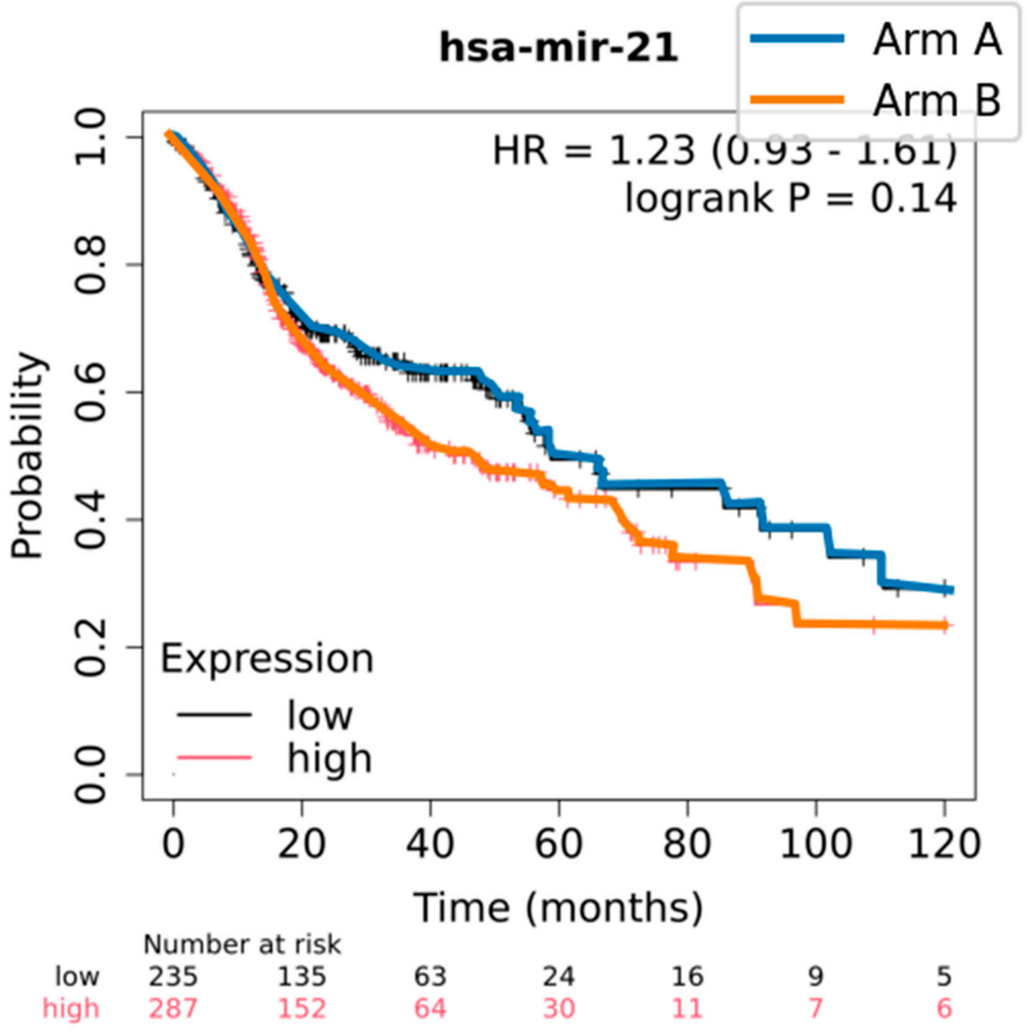


## Numbers-at-Risk

| time | arm1 | arm2 |
|---|---|---|
| 0 | 235 | 287 |
| 20 | 135 | 152 |
| 40 | 63 | 64 |
| 60 | 24 | 30 |
| 80 | 16 | 11 |

| | | |
|---|---|---|
| 100 | 9 | 7 |
| 120 | 5 | 6 |

## Curve data (A & B)

| t_A | S_A | t_B | S_B |
|---|---|---|---|
| 4.09756 | 0.997059 | 3.80488 | 0.997059 |
| 4.97561 | 0.994118 | 11.122 | 0.911765 |
| 8.4878 | 0.952941 | 15.8049 | 0.838235 |
| 10.5366 | 0.923529 | 18.1463 | 0.788235 |
| 12.2927 | 0.885294 | 19.6098 | 0.744118 |
| 14.6341 | 0.852941 | 22.2439 | 0.705882 |
| 18.439 | 0.785294 | 26.9268 | 0.65 |
| 21.9512 | 0.744118 | 29.2683 | 0.632353 |
| 25.1707 | 0.708824 | 34.5366 | 0.594118 |
| 28.9756 | 0.7 | 38.9268 | 0.561765 |
| 31.3171 | 0.688235 | 43.0244 | 0.529412 |
| 33.3659 | 0.673529 | 46.2439 | 0.520588 |
| 35.7073 | 0.658824 | 48.5854 | 0.520588 |
| 38.0488 | 0.65 | 49.7561 | 0.511765 |
| 40.0976 | 0.647059 | 51.2195 | 0.494118 |
| 44.1951 | 0.641176 | 59.4146 | 0.485294 |
| 50.0488 | 0.641176 | 59.7073 | 0.470588 |
| 50.6341 | 0.629412 | 60.878 | 0.470588 |
| 52.0976 | 0.620588 | 61.7561 | 0.461765 |
| 53.2683 | 0.602941 | 63.5122 | 0.461765 |
| 55.6098 | 0.585294 | 63.5122 | 0.45 |

| | | | |
|---|---|---|---|
| 56.1951 | 0.585294 | 69.9512 | 0.447059 |
| 57.6585 | 0.564706 | 71.122 | 0.429412 |
| 57.9512 | 0.564706 | 72 | 0.411765 |
| 58.2439 | 0.564706 | 74.0488 | 0.394118 |
| 58.8293 | 0.55 | 74.0488 | 0.385294 |
| 60.5854 | 0.55 | 79.0244 | 0.361765 |
| 60.5854 | 0.532353 | 79.3171 | 0.361765 |
| 61.1707 | 0.517647 | 90.439 | 0.355882 |
| 67.9024 | 0.494118 | 91.3171 | 0.329412 |
| 68.1951 | 0.494118 | 91.6098 | 0.329412 |
| 68.4878 | 0.470588 | 91.9024 | 0.3 |
| 68.7805 | 0.470588 | 97.4634 | 0.291176 |
| 86.3415 | 0.470588 | 97.7561 | 0.261765 |
| 87.5122 | 0.441176 | 119.707 | 0.258824 |
| 92.1951 | 0.441176 | | |
| 92.7805 | 0.405882 | | |
| 102.146 | 0.405882 | | |
| 102.732 | 0.367647 | | |
| 110.341 | 0.364706 | | |
| 110.341 | 0.323529 | | |
| 120.585 | 0.311765 | | |
