# Supplementary material for: Prognostic Impact of miR-34a in Head and Neck Squamous Cell Carcinoma: A Systematic Review with Meta-Analysis and Trial Sequential Analysis
Source: Int J Mol Sci. 2026 May 29;27(11):4909. doi: 10.3390/ijms27114909 (PMC13256702; doi:10.3390/ijms27114909)
Supplement: Supplementary file 1 [file ijms-27-04909-s001.zip › validation/Set 2 — TCGAKM Plotter database-derived validation/TCGA mir 31 HNSCC/KM2HR_report.pdf]

KM2HR — Kaplan–Meier → Hazard Ratio (Tierney method)

2026-05-11 08:03

Author: Dioguardi Mario — Università di Foggia

Time axis: 0.0 – 60.0 | **Initial N:** N1=185, N2=337 | **Use NAR:** Yes

Result

HR (A vs B) = 0.819 (95% CI 0.618 – 1.086)

HR (B vs A) = 1.221 (95% CI 0.921 – 1.619)

logHR_AB = -0.1998, SE = 0.1440, O−E = -9.639, V = 48.244

Traced curves

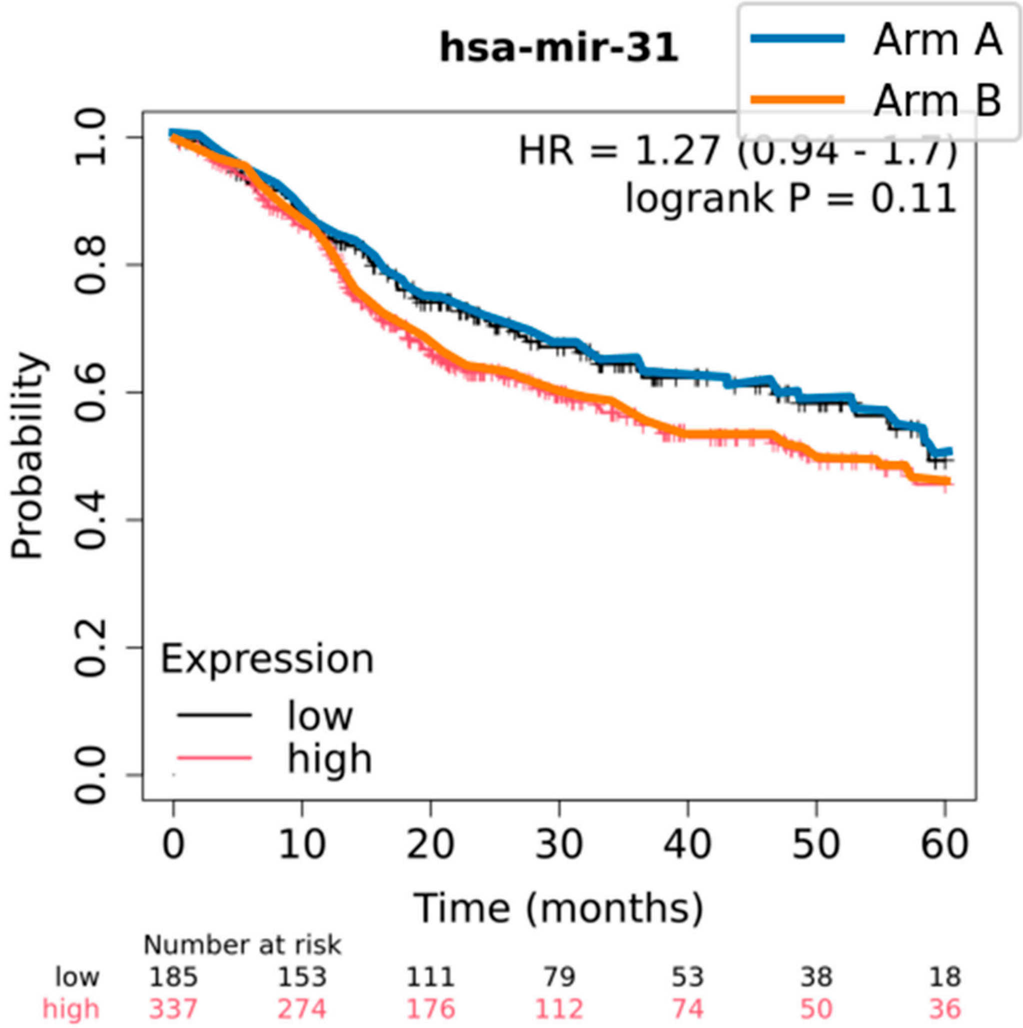

Numbers-at-Risk

time	arm1	arm2
0	185	337
10	153	274
20	111	176
30	79	112
40	53	74

50	38	50
60	18	36

Curve data (A & B)

t_A	S_A	t_B	S_B
-0.152284	1	0	0.993846
1.82741	1	3.35025	0.963077
3.35025	0.975385	5.48223	0.950769
5.48223	0.947692	6.54822	0.923077
7.91878	0.923077	8.22335	0.892308
9.13706	0.901538	10.8122	0.852308
10.203	0.876923	11.8782	0.824615
10.9645	0.861538	12.9442	0.790769
12.6396	0.843077	14.0102	0.756923
14.0102	0.833846	16.1421	0.72
15.533	0.809231	19.3401	0.683077
16.2944	0.787692	20.8629	0.658462
17.665	0.772308	22.6904	0.636923
17.9695	0.763077	25.736	0.627692
19.1878	0.747692	27.7157	0.612308
20.7107	0.744615	29.8477	0.596923
21.7766	0.735385	31.8274	0.587692
23.9086	0.716923	33.9594	0.581538
25.2792	0.707692	35.4822	0.563077
27.5635	0.692308	36.5482	0.550769
29.3909	0.673846	39.5939	0.529231

31.2183	0.673846	46.4467	0.529231
31.9797	0.661538	47.5127	0.513846
33.0457	0.646154	48.8832	0.507692
35.9391	0.646154	49.9492	0.492308
36.3959	0.627692	54.5178	0.489231
42.9442	0.618462	54.9746	0.48
42.9442	0.606154	56.802	0.48
46.2944	0.606154	57.2589	0.461538
46.9036	0.593846	60	0.455385
48.4264	0.593846		
48.5787	0.584615		
52.5381	0.584615		
52.8426	0.569231		
55.2792	0.566154		
56.1929	0.544615		
58.1726	0.538462		
58.3249	0.52		
58.6294	0.513846		
59.0863	0.498462		
60.1523	0.498462		
