# Supplementary material for: Prognostic Impact of miR-34a in Head and Neck Squamous Cell Carcinoma: A Systematic Review with Meta-Analysis and Trial Sequential Analysis
Source: Int J Mol Sci. 2026 May 29;27(11):4909. doi: 10.3390/ijms27114909 (PMC13256702; doi:10.3390/ijms27114909)
Supplement: Supplementary file 1 [file ijms-27-04909-s001.zip › validation/Set 2 — TCGAKM Plotter database-derived validation/TGCA mir 100 HNSCC/KM2HR_report.pdf]

KM2HR — Kaplan–Meier → Hazard Ratio (Tierney method)

2026-05-12 08:15

Author: Dioguardi Mario — Università di Foggia

Time axis: 0.0 – 60.0 | **Initial N:** N1=139, N2=383 | **Use NAR:** Yes

Result

HR (A vs B) = 0.551 (95% CI 0.406 – 0.749)

HR (B vs A) = 1.814 (95% CI 1.336 – 2.463)

logHR_AB = -0.5954, SE = 0.1560, O−E = -24.457, V = 41.078

Traced curves

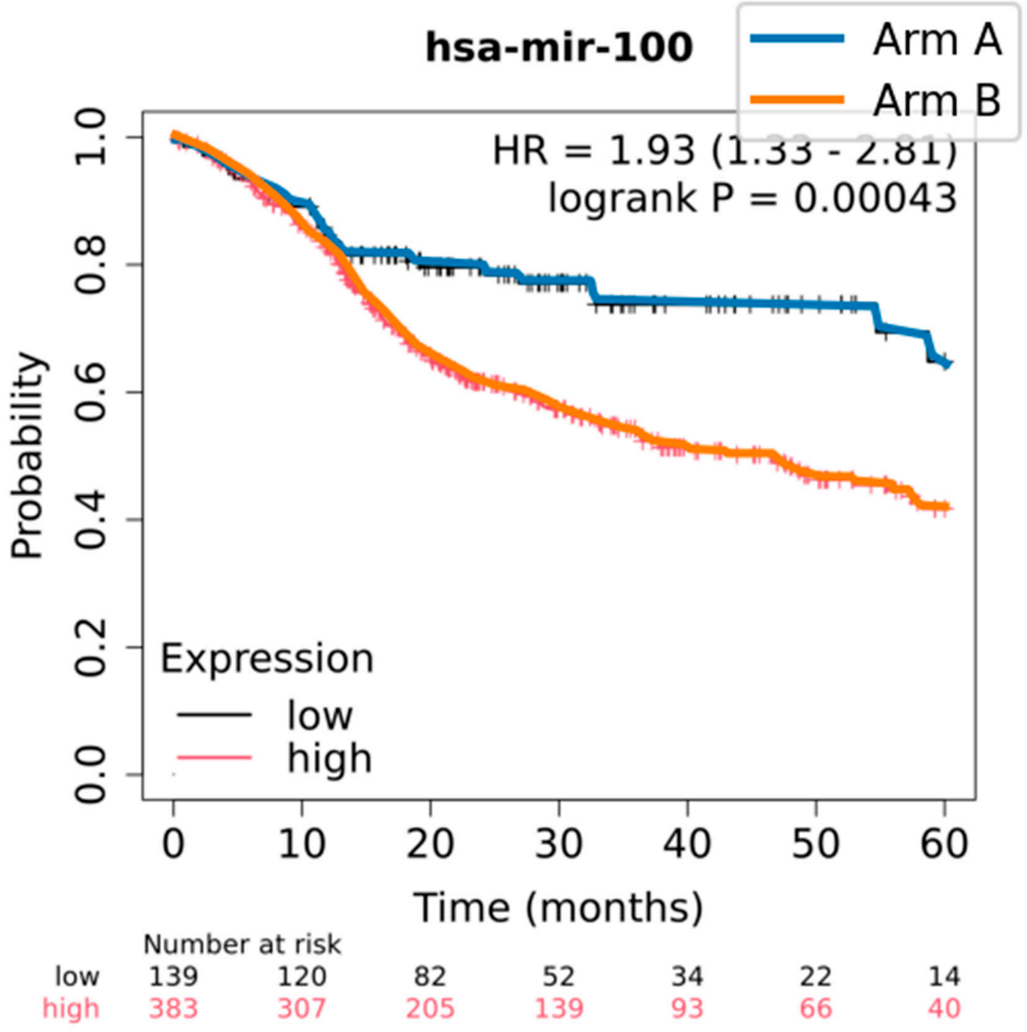

Numbers-at-Risk

time	arm1	arm2
0	139	383
10	120	307
20	82	205
30	52	139
40	34	93

50	22	66
60	14	40

Curve data (A & B)

t_A	S_A	t_B	S_B
0.11215	0.993228	0.11215	1
0.560748	0.993228	2.57944	0.979684
1.34579	0.986456	5.83178	0.939052
2.13084	0.979684	7.85047	0.905192
3.47664	0.963883	9.53271	0.873589
4.26168	0.952596	10.5421	0.848758
4.93458	0.945824	11.7757	0.832957
6.16822	0.93228	12.8972	0.812641
7.17757	0.923251	13.7944	0.785553
7.96262	0.916479	14.8037	0.75395
8.63551	0.907449	15.7009	0.738149
9.08411	0.89842	16.486	0.722348
10.5421	0.891648	17.6075	0.697517
11.215	0.869074	18.9533	0.670429
11.4393	0.86456	20.8598	0.647856
11.6636	0.853273	22.5421	0.629797
12.1121	0.846501	23.215	0.620767
12.3364	0.837472	25.1215	0.607223
13.0093	0.823928	27.2523	0.600451
13.2336	0.817156	29.0467	0.58465
18.2804	0.814898	30.0561	0.573363

18.729	0.803612	31.6262	0.562077
24.1121	0.79684	33.3084	0.55079
24.2243	0.785553	34.8785	0.541761
26.6916	0.783296	36	0.537246
27.1402	0.772009	36.4486	0.530474
32.4112	0.772009	37.2336	0.521445
32.6355	0.751693	39.7009	0.514673
32.8598	0.742664	40.2617	0.507901
54.5047	0.731377	42.8411	0.505643
54.8411	0.699774	43.0654	0.501129
58.5421	0.68623	46.5421	0.501129
58.9907	0.654628	47.4393	0.485327
60.1121	0.641084	48.6729	0.474041
		50.3551	0.465011
		52.5981	0.465011
		53.0467	0.458239
		55.8505	0.453725
		55.9626	0.444695
		57.0841	0.444695
		57.6449	0.428894
		58.0935	0.419865
		60	0.417607
