# Supplementary material for: Prognostic Impact of miR-34a in Head and Neck Squamous Cell Carcinoma: A Systematic Review with Meta-Analysis and Trial Sequential Analysis
Source: Int J Mol Sci. 2026 May 29;27(11):4909. doi: 10.3390/ijms27114909 (PMC13256702; doi:10.3390/ijms27114909)
Supplement: Supplementary file 1 [file ijms-27-04909-s001.zip › validation/Set 2 — TCGAKM Plotter database-derived validation/TGCA mir 197 HNSCC/KM2HR_report.pdf]

KM2HR — Kaplan–Meier → Hazard Ratio (Tierney method)

2026-05-11 08:14

Author: Dioguardi Mario — Università di Foggia

Time axis: 0.0 – 60.0 | **Initial N:** N1=229, N2=293 | **Use NAR:** Yes

Result

HR (A vs B) = 0.693 (95% CI 0.524 – 0.918)

HR (B vs A) = 1.442 (95% CI 1.089 – 1.909)

logHR_AB = -0.3661, SE = 0.1432, O−E = -17.866, V = 48.799

Traced curves

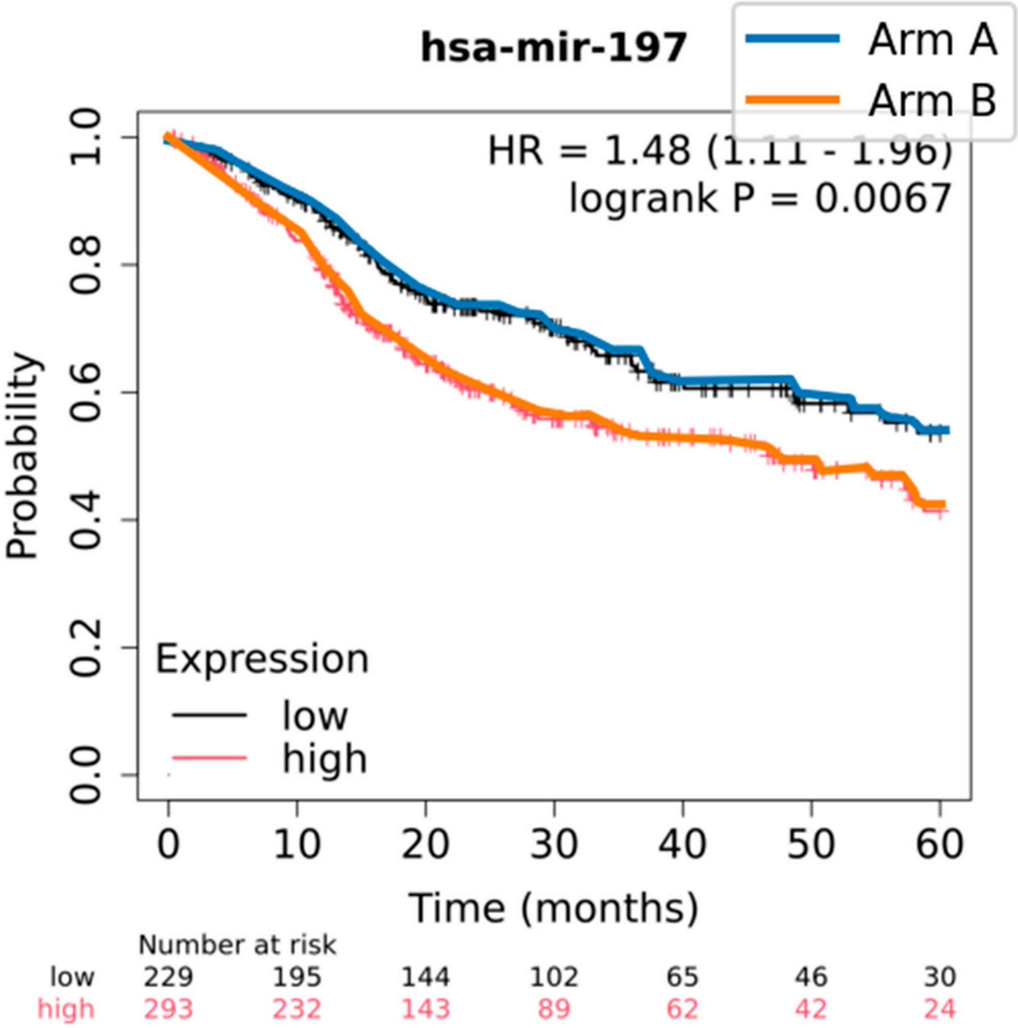

Numbers-at-Risk

time	arm1	arm2
0	229	293
10	195	232
20	144	143
30	102	89
40	65	62

50	46	42
60	30	24

Curve data (A & B)

t_A	S_A	t_B	S_B
-0.152284	0.987805	-0.152284	0.993902
3.65482	0.972561	5.02538	0.917683
5.17766	0.954268	6.85279	0.890244
8.83249	0.914634	10.203	0.844512
10.9645	0.893293	12.0305	0.789634
12.9442	0.865854	13.8579	0.753049
14.7716	0.829268	14.9239	0.716463
16.599	0.79878	21.1675	0.631098
19.3401	0.759146	23.9086	0.606707
22.3858	0.731707	28.6294	0.567073
25.5838	0.731707	31.066	0.557927
27.1066	0.719512	32.5888	0.557927
28.7817	0.716463	33.5025	0.551829
29.8477	0.695122	35.3299	0.533537
31.9797	0.685976	36.3959	0.527439
34.4162	0.661585	43.2487	0.521341
36.5482	0.661585	45.9898	0.512195
37.4619	0.625	46.599	0.509146
39.5939	0.612805	47.665	0.490854
48.2741	0.612805	50.2538	0.490854
48.8832	0.594512	50.7107	0.472561

52.9949	0.585366	54.2132	0.472561
53.1472	0.570122	54.6701	0.466463
54.9746	0.570122	56.9543	0.466463
55.5838	0.557927	57.868	0.442073
57.7157	0.551829	58.1726	0.42378
58.4772	0.536585	58.6294	0.420732
60.3046	0.536585	60	0.420732
