# Supplementary figures and images for: Prognostic Impact of miR-34a in Head and Neck Squamous Cell Carcinoma: A Systematic Review with Meta-Analysis and Trial Sequential Analysis
Source: Int J Mol Sci. 2026 May 29;27(11):4909. doi: 10.3390/ijms27114909 (PMC13256702; doi:10.3390/ijms27114909)

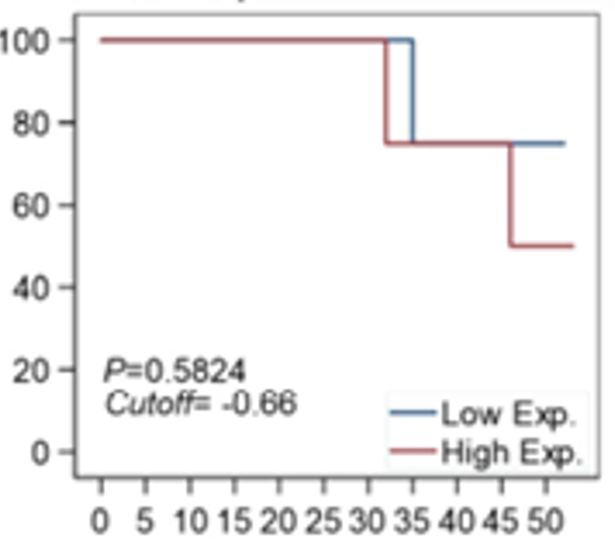

Supplement: Supplementary file 1 [file ijms-27-04909-s001.zip › KM2HR workflow/Piotrowski/Immagine 2025-10-30 082142.png]

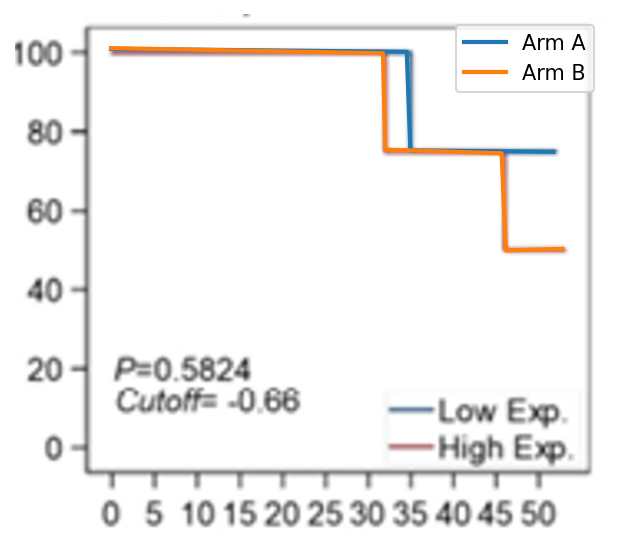

Supplement: Supplementary file 1 [file ijms-27-04909-s001.zip › KM2HR workflow/Piotrowski/KM_HR_report_plot.png]

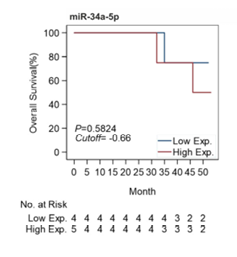

Supplement: Supplementary file 1 [file ijms-27-04909-s001.zip › KM2HR workflow/Piotrowski/orofai.png]

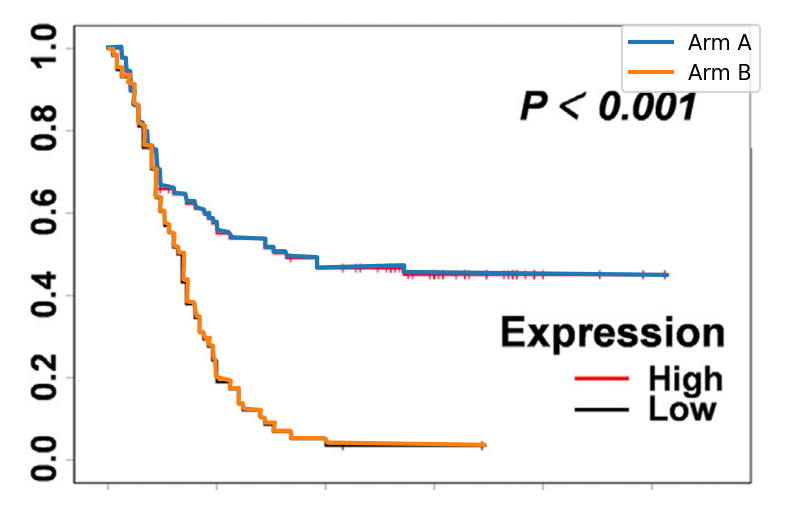

Supplement: Supplementary file 1 [file ijms-27-04909-s001.zip › KM2HR workflow/ren/KM_HR_report_plot.png]

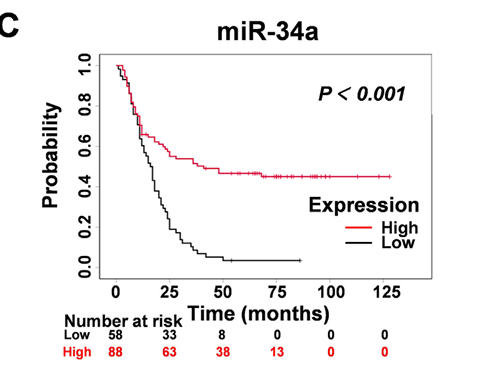

Supplement: Supplementary file 1 [file ijms-27-04909-s001.zip › KM2HR workflow/ren/ren.png]

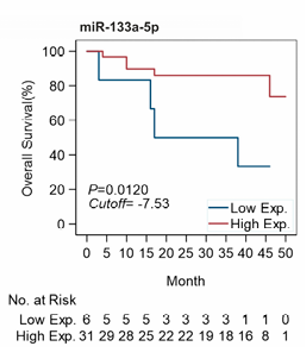

Supplement: Supplementary file 1 [file ijms-27-04909-s001.zip › validation/Set 1 — Published-paper validation/mir 133a oral OS Piotrowski et al.,/133a os oral.png]

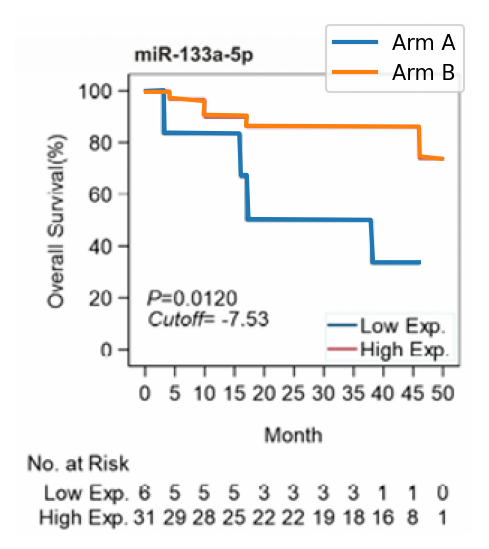

Supplement: Supplementary file 1 [file ijms-27-04909-s001.zip › validation/Set 1 — Published-paper validation/mir 133a oral OS Piotrowski et al.,/KM2HR_report_plot.png]

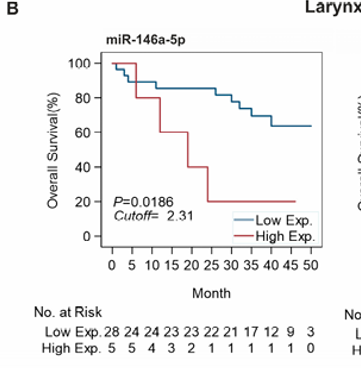

Supplement: Supplementary file 1 [file ijms-27-04909-s001.zip › validation/Set 1 — Published-paper validation/mir 146a larinx OS Piotrowski et al.,/146a os larinx.png]

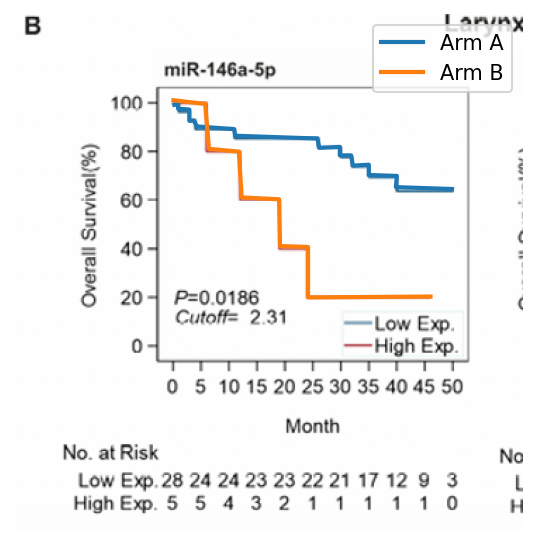

Supplement: Supplementary file 1 [file ijms-27-04909-s001.zip › validation/Set 1 — Published-paper validation/mir 146a larinx OS Piotrowski et al.,/KM2HR_report_plot.png]

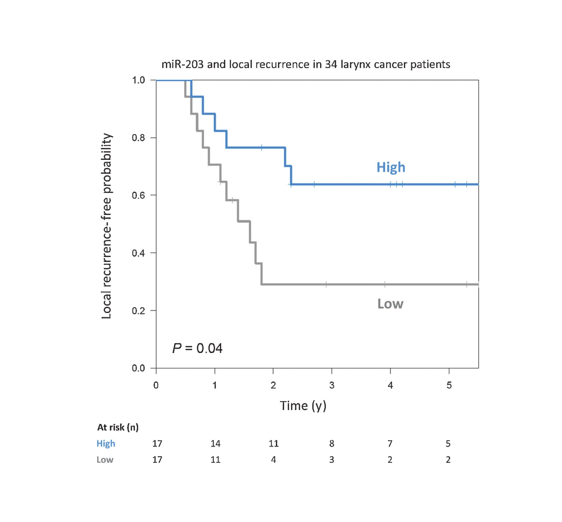

Supplement: Supplementary file 1 [file ijms-27-04909-s001.zip › validation/Set 1 — Published-paper validation/mir 203 larinx LR de Jong et al.,/Immagine 2026-05-11 061234.png]

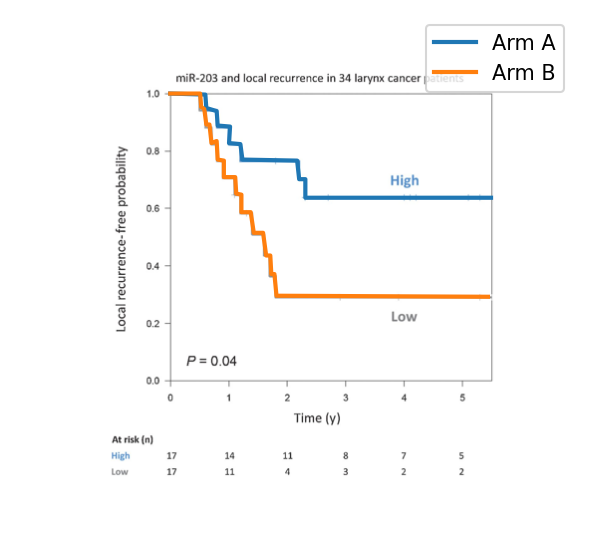

Supplement: Supplementary file 1 [file ijms-27-04909-s001.zip › validation/Set 1 — Published-paper validation/mir 203 larinx LR de Jong et al.,/KM2HR_report_plot.png]

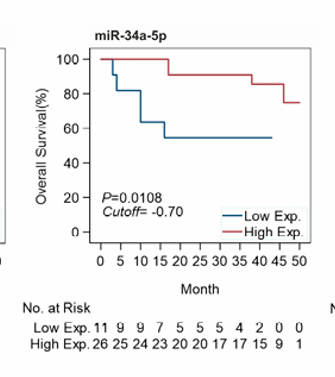

Supplement: Supplementary file 1 [file ijms-27-04909-s001.zip › validation/Set 1 — Published-paper validation/mir 34a oral OS Piotrowski et al.,/34 a os oral.png]

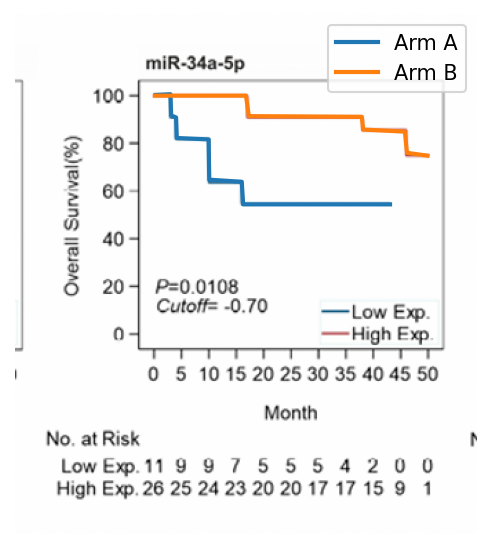

Supplement: Supplementary file 1 [file ijms-27-04909-s001.zip › validation/Set 1 — Published-paper validation/mir 34a oral OS Piotrowski et al.,/KM2HR_report_plot.png]

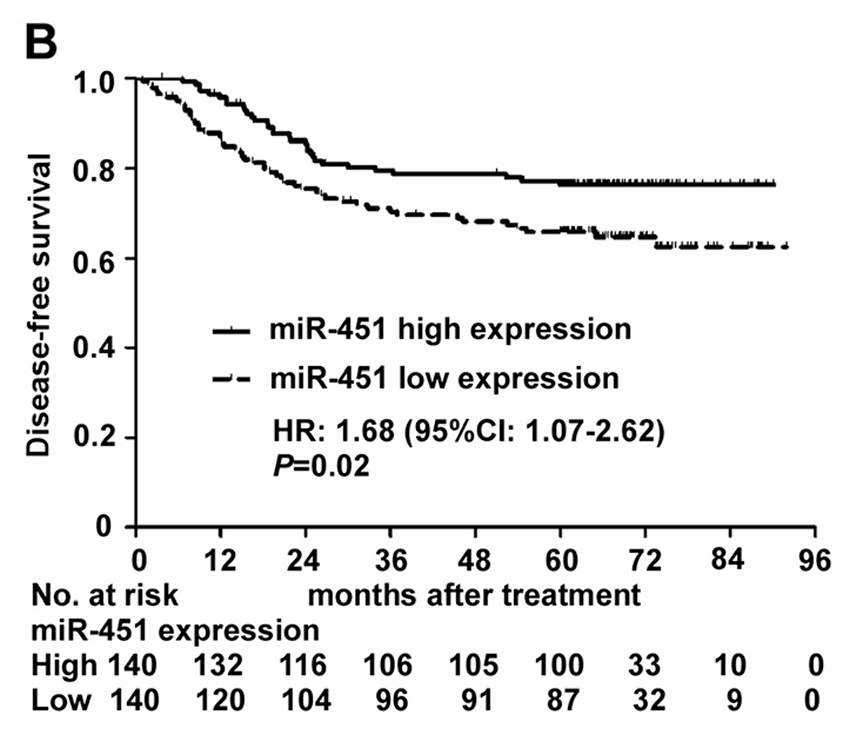

Supplement: Supplementary file 1 [file ijms-27-04909-s001.zip › validation/Set 1 — Published-paper validation/mir 451 NPC DFS Liu et al.,/Immagine 2026-05-11 064522.png]

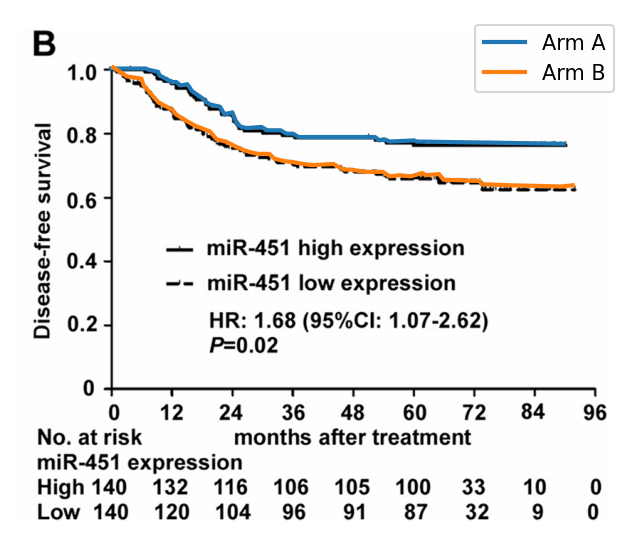

Supplement: Supplementary file 1 [file ijms-27-04909-s001.zip › validation/Set 1 — Published-paper validation/mir 451 NPC DFS Liu et al.,/KM2HR_report_plot.png]

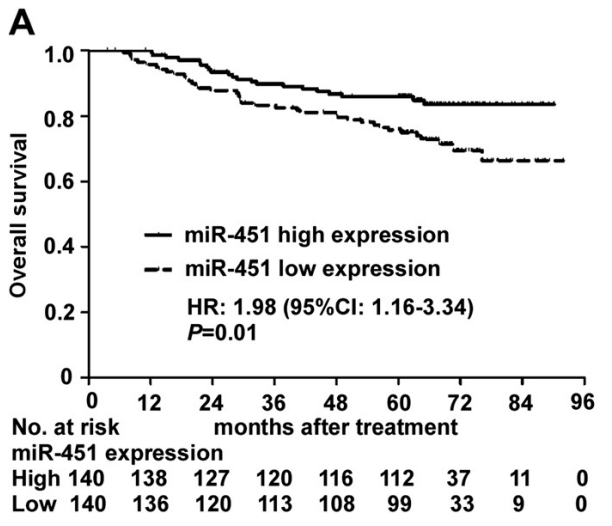

Supplement: Supplementary file 1 [file ijms-27-04909-s001.zip › validation/Set 1 — Published-paper validation/mir 451 NPC OS Liu et al.,/Immagine 2026-05-11 063122.png]

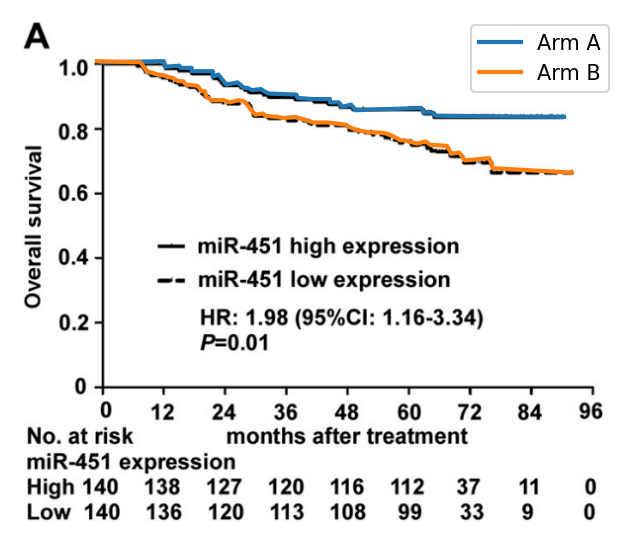

Supplement: Supplementary file 1 [file ijms-27-04909-s001.zip › validation/Set 1 — Published-paper validation/mir 451 NPC OS Liu et al.,/KM2HR_report_plot.png]

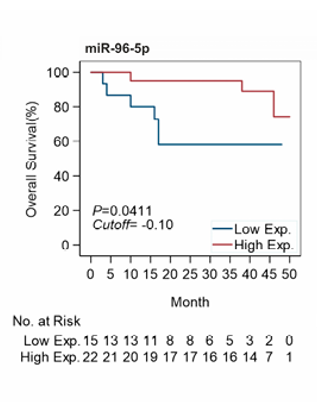

Supplement: Supplementary file 1 [file ijms-27-04909-s001.zip › validation/Set 1 — Published-paper validation/mir 96 oral OS Piotrowski et al.,/96 os oral.png]

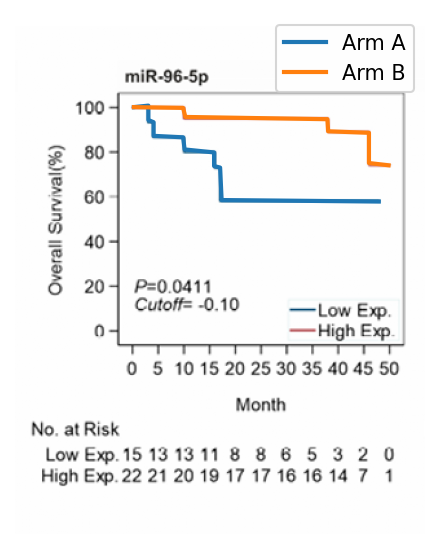

Supplement: Supplementary file 1 [file ijms-27-04909-s001.zip › validation/Set 1 — Published-paper validation/mir 96 oral OS Piotrowski et al.,/KM2HR_report_plot.png]

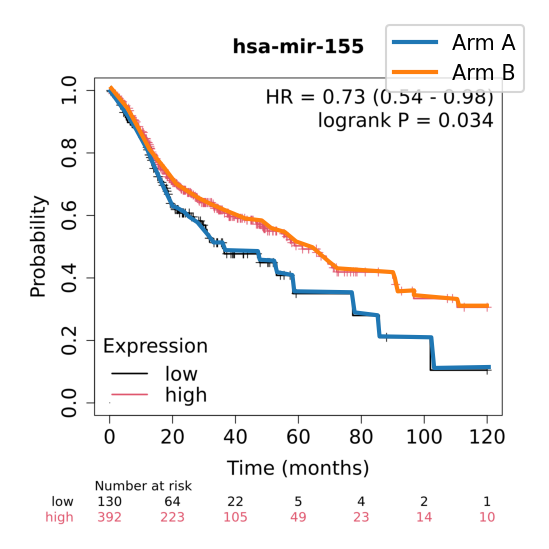

Supplement: Supplementary file 1 [file ijms-27-04909-s001.zip › validation/Set 2 — TCGAKM Plotter database-derived validation/TCGA mir 155 HNSCC/KM2HR_report_plot.png]

# hsa-mir-155

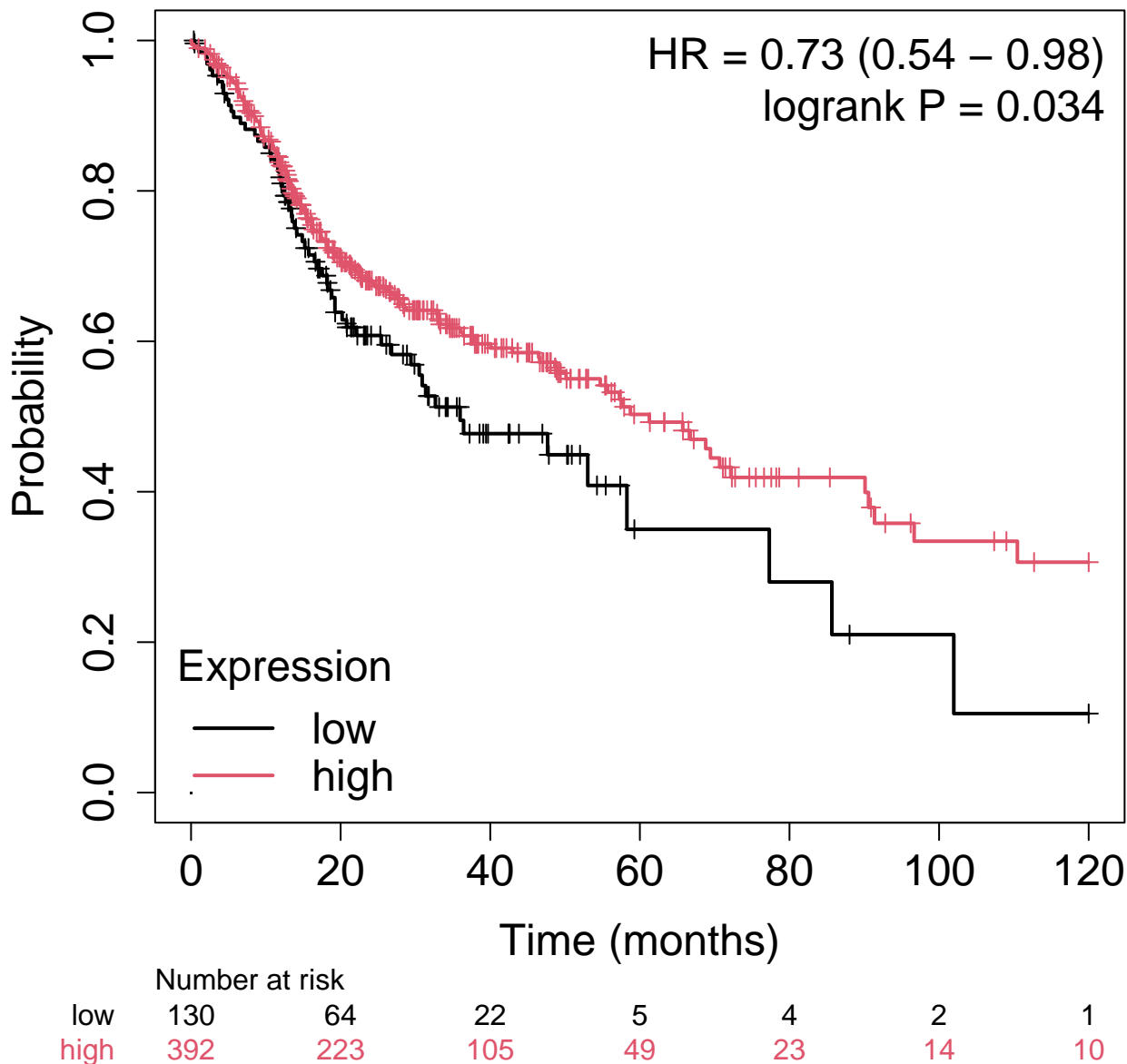

Supplement: Supplementary file 1 [file ijms-27-04909-s001.zip › validation/Set 2 — TCGAKM Plotter database-derived validation/TCGA mir 155 HNSCC/km_260511_051451_887000_6a0165cbd88f5_hsa-mir-155.pdf]

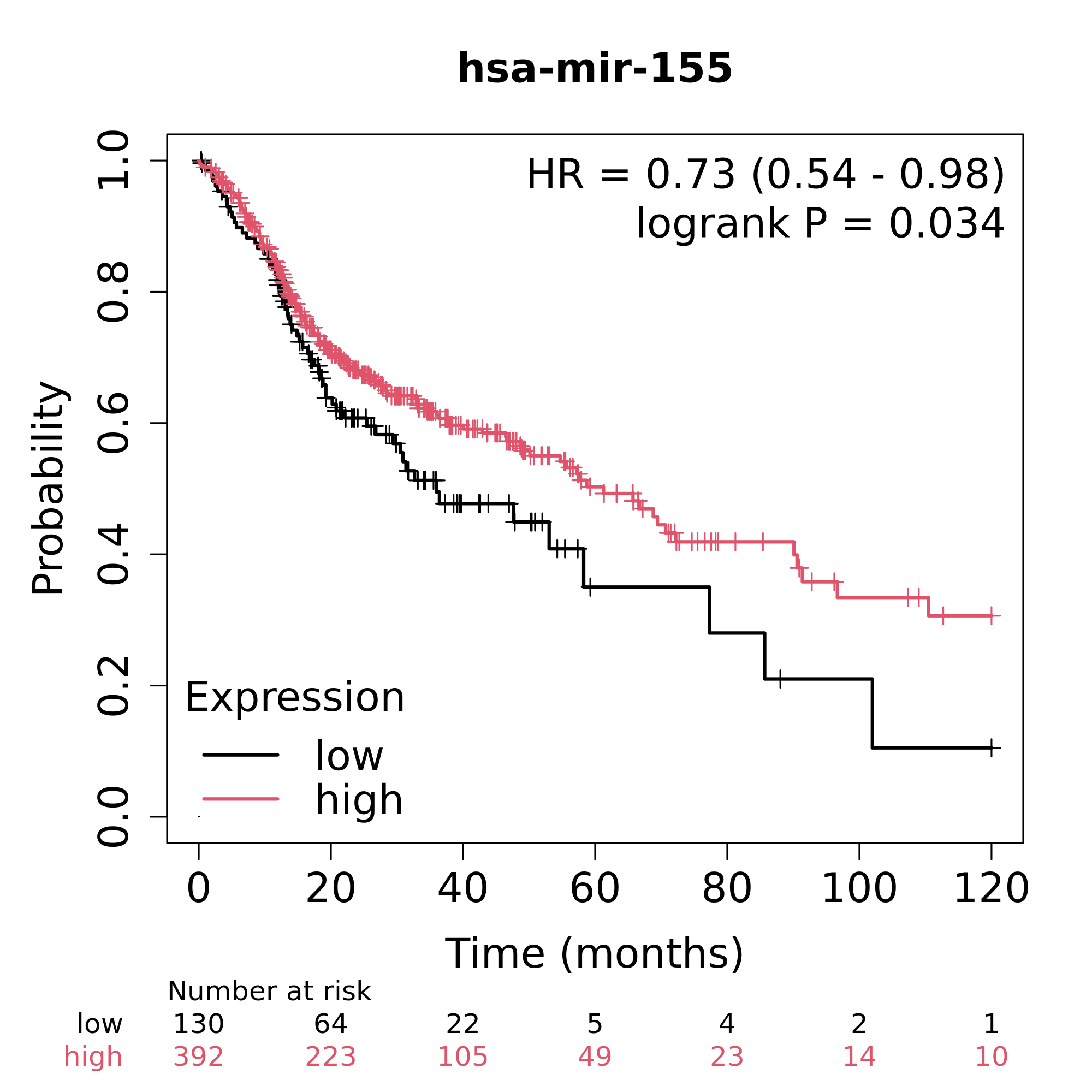

Supplement: Supplementary file 1 [file ijms-27-04909-s001.zip › validation/Set 2 — TCGAKM Plotter database-derived validation/TCGA mir 155 HNSCC/km_260511_051451_887000_6a0165cbd88f5_hsa-mir-155.png]

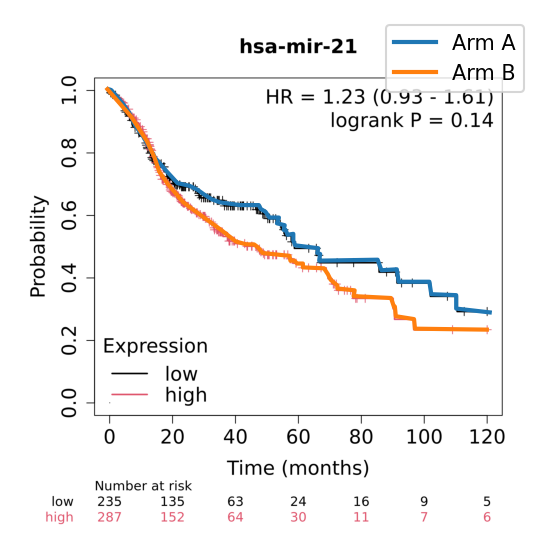

Supplement: Supplementary file 1 [file ijms-27-04909-s001.zip › validation/Set 2 — TCGAKM Plotter database-derived validation/TCGA mir 21 HNSCC/KM2HR_report_plot.png]

# hsa-mir-21

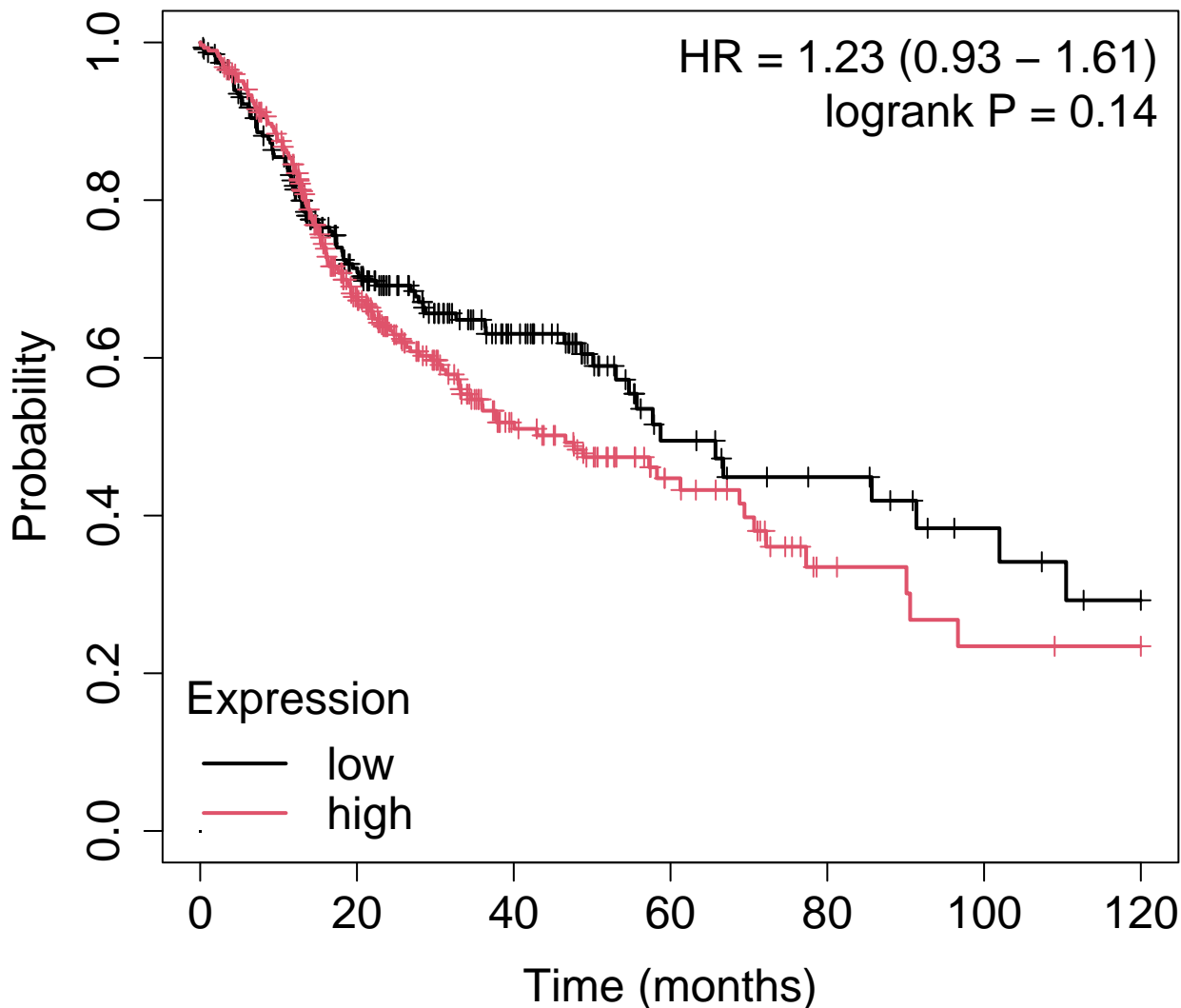

Number at risk

|      |     |     |    |    |    |   |   |
|------|-----|-----|----|----|----|---|---|
| low  | 235 | 135 | 63 | 24 | 16 | 9 | 5 |
| high | 287 | 152 | 64 | 30 | 11 | 7 | 6 |

Supplement: Supplementary file 1 [file ijms-27-04909-s001.zip › validation/Set 2 — TCGAKM Plotter database-derived validation/TCGA mir 21 HNSCC/km_260511_050121_994000_6a0162a1f2b1c_hsa-mir-21.pdf]

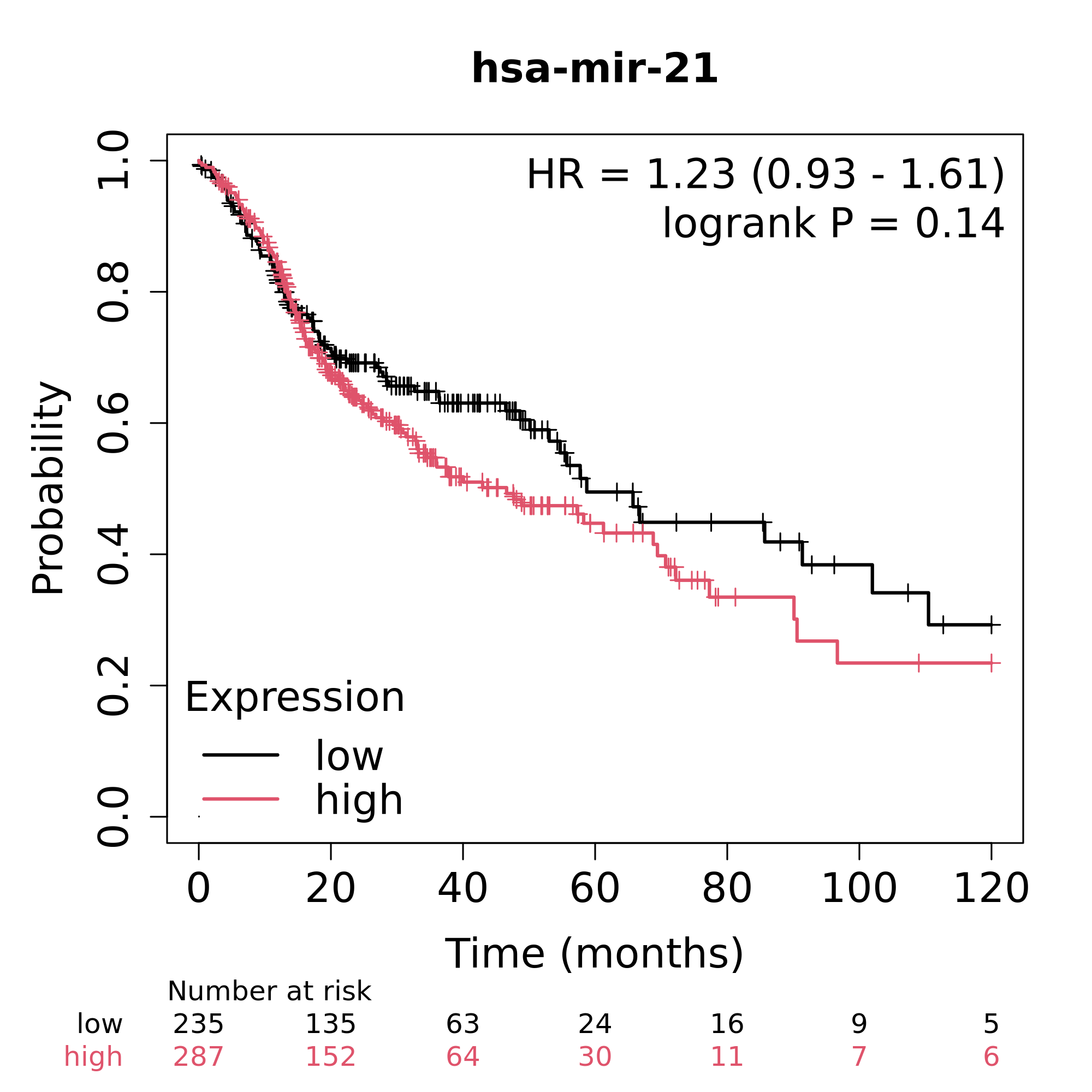

Supplement: Supplementary file 1 [file ijms-27-04909-s001.zip › validation/Set 2 — TCGAKM Plotter database-derived validation/TCGA mir 21 HNSCC/km_260511_050121_994000_6a0162a1f2b1c_hsa-mir-21.png]

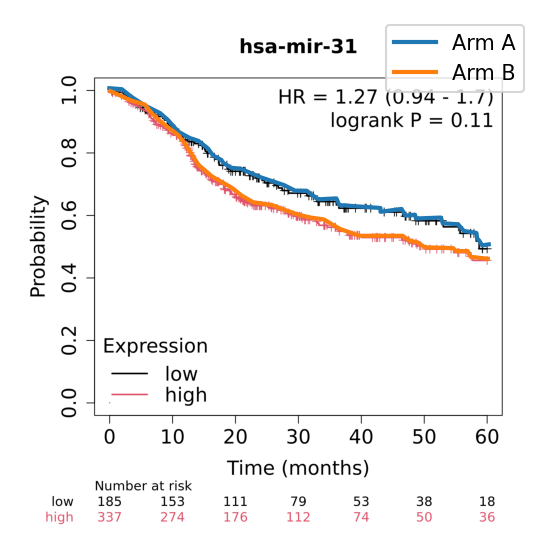

Supplement: Supplementary file 1 [file ijms-27-04909-s001.zip › validation/Set 2 — TCGAKM Plotter database-derived validation/TCGA mir 31 HNSCC/KM2HR_report_plot.png]

# hsa-mir-31

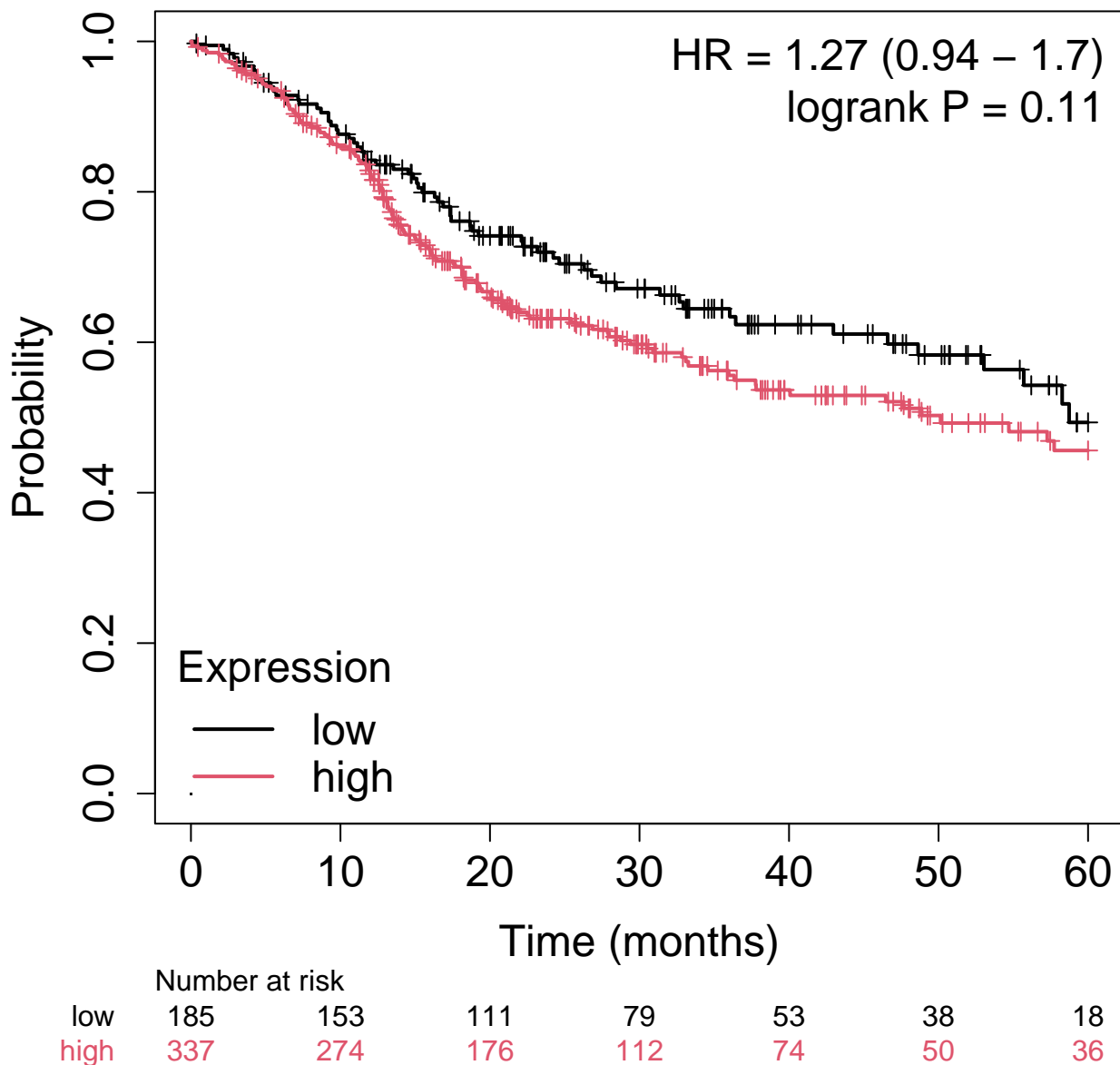

Supplement: Supplementary file 1 [file ijms-27-04909-s001.zip › validation/Set 2 — TCGAKM Plotter database-derived validation/TCGA mir 31 HNSCC/km_260511_055035_861700_6a016e2bd25ee_hsa-mir-31.pdf]

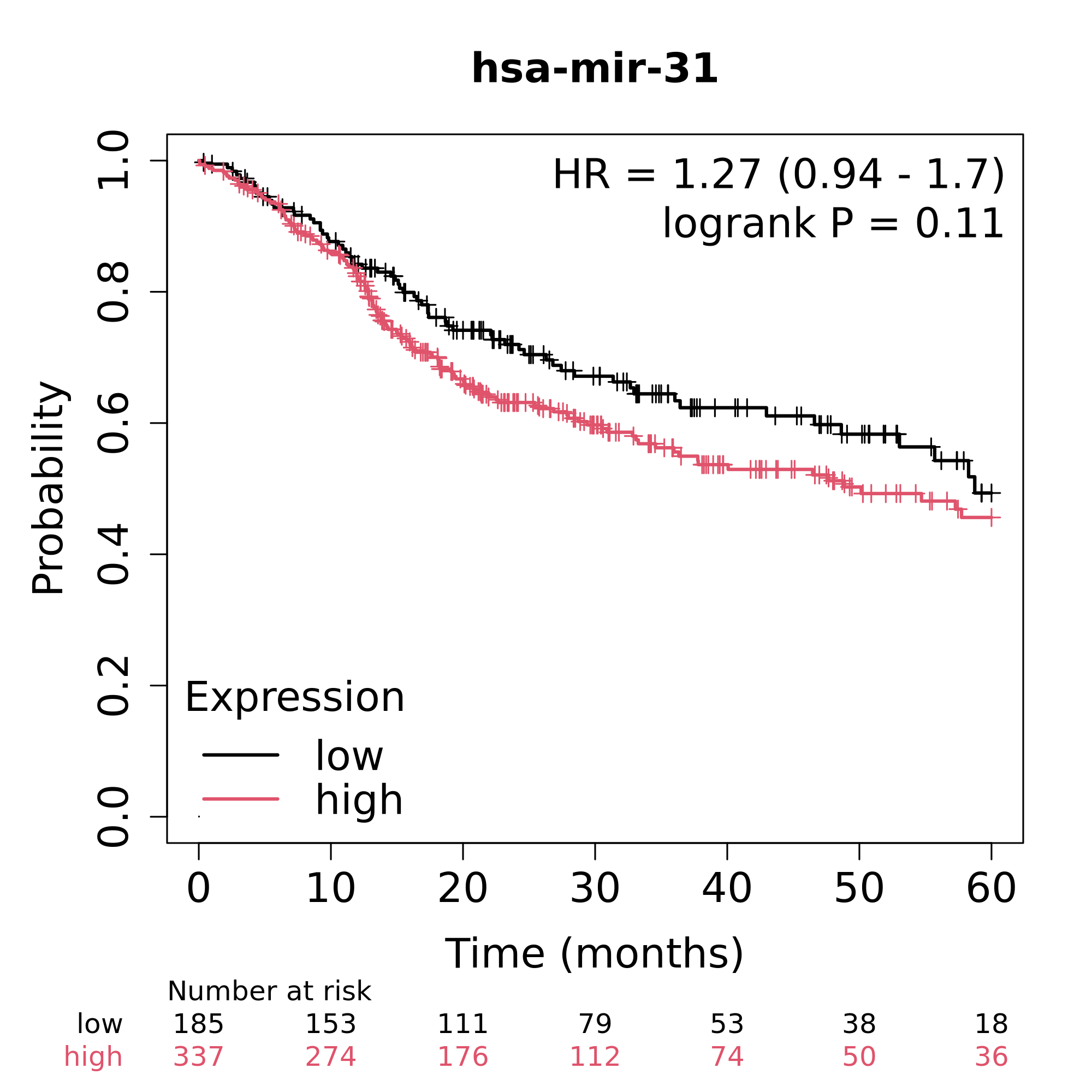

Supplement: Supplementary file 1 [file ijms-27-04909-s001.zip › validation/Set 2 — TCGAKM Plotter database-derived validation/TCGA mir 31 HNSCC/km_260511_055035_861700_6a016e2bd25ee_hsa-mir-31.png]

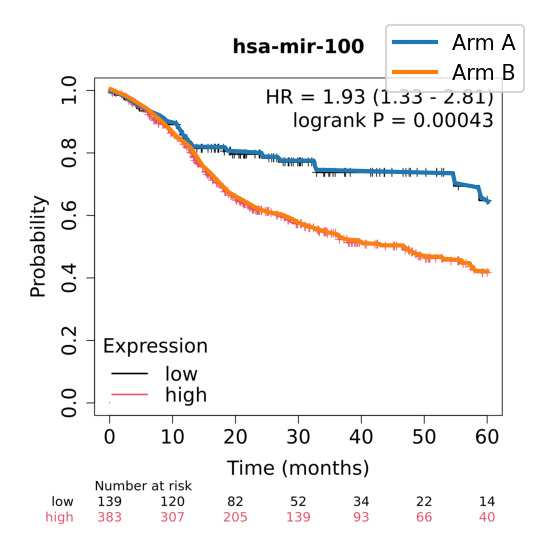

Supplement: Supplementary file 1 [file ijms-27-04909-s001.zip › validation/Set 2 — TCGAKM Plotter database-derived validation/TGCA mir 100 HNSCC/KM2HR_report_plot.png]

# hsa-mir-100

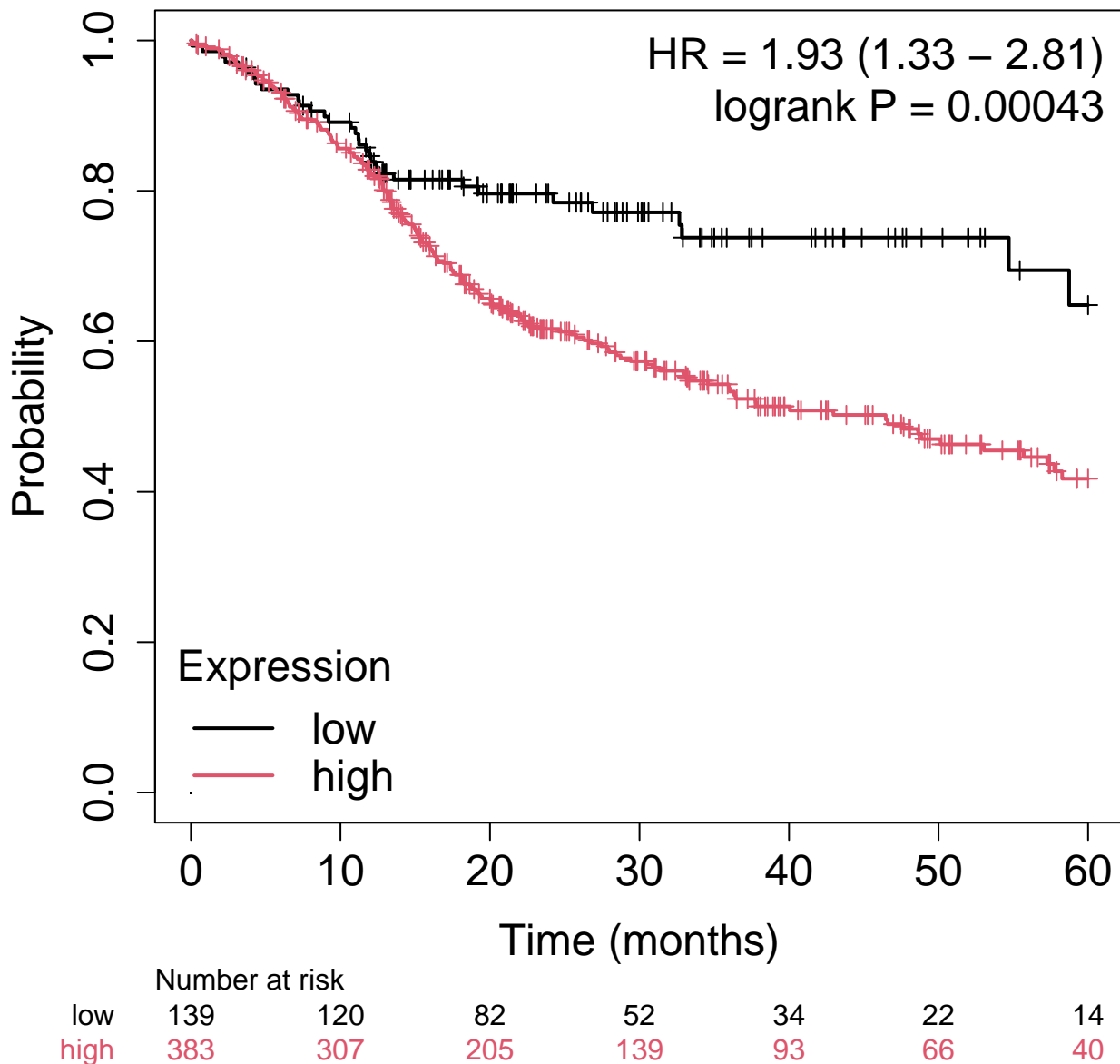

Supplement: Supplementary file 1 [file ijms-27-04909-s001.zip › validation/Set 2 — TCGAKM Plotter database-derived validation/TGCA mir 100 HNSCC/km_260512_055832_806400_6a02c188c4e09_hsa-mir-100.pdf]

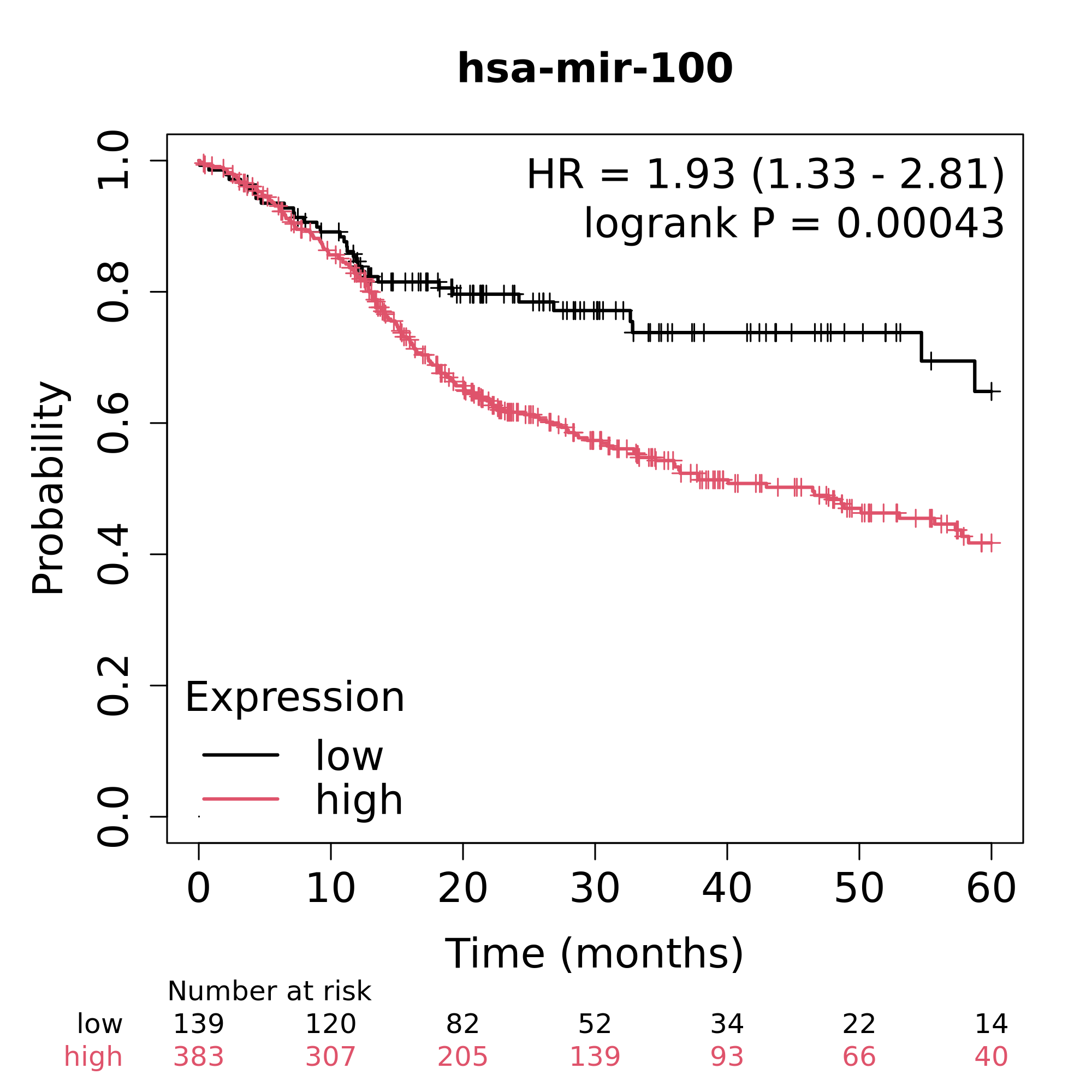

Supplement: Supplementary file 1 [file ijms-27-04909-s001.zip › validation/Set 2 — TCGAKM Plotter database-derived validation/TGCA mir 100 HNSCC/km_260512_055832_806400_6a02c188c4e09_hsa-mir-100.png]

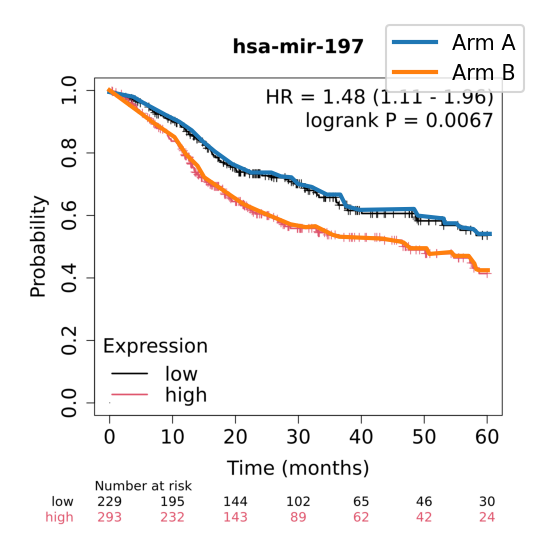

Supplement: Supplementary file 1 [file ijms-27-04909-s001.zip › validation/Set 2 — TCGAKM Plotter database-derived validation/TGCA mir 197 HNSCC/KM2HR_report_plot.png]

# hsa-mir-197

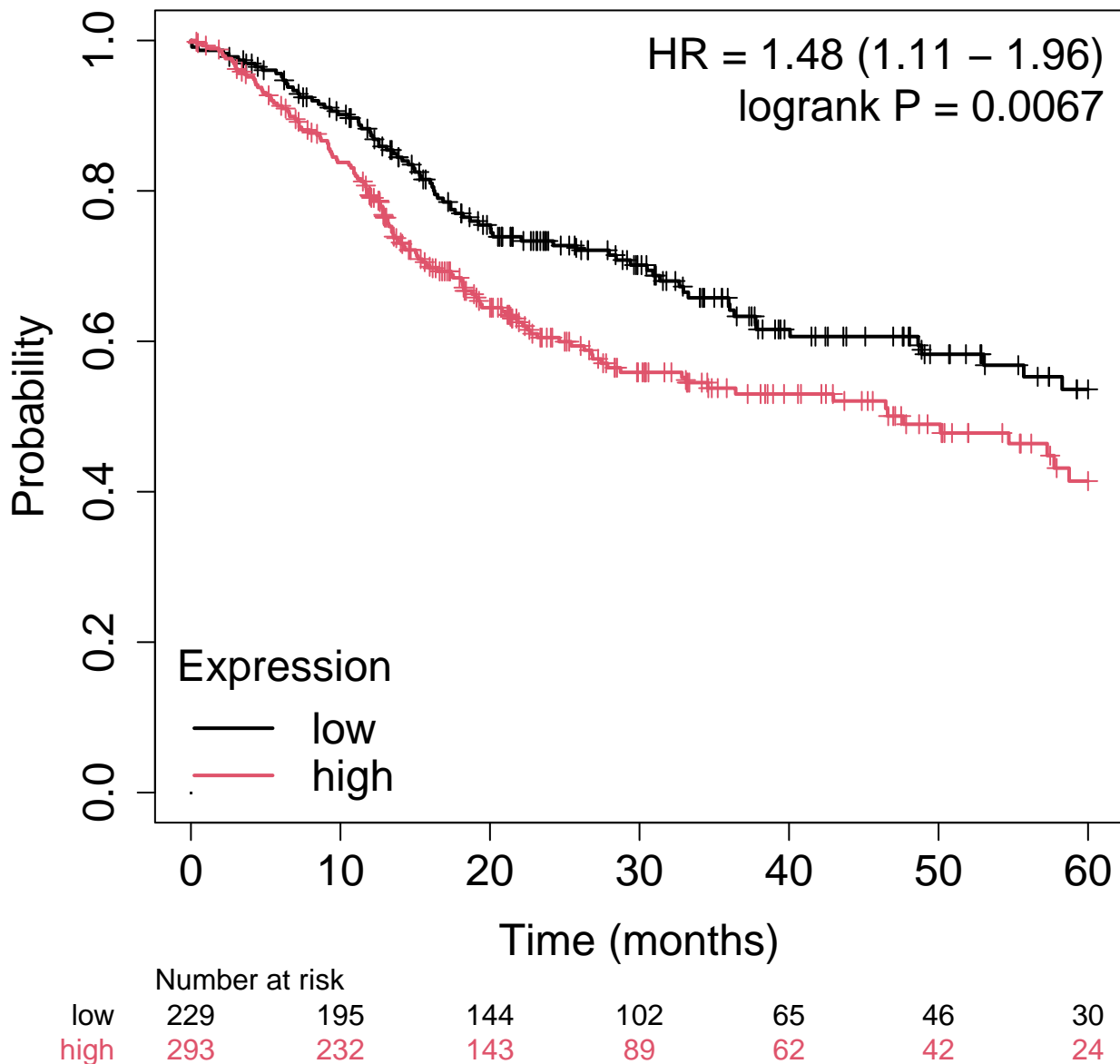

Supplement: Supplementary file 1 [file ijms-27-04909-s001.zip › validation/Set 2 — TCGAKM Plotter database-derived validation/TGCA mir 197 HNSCC/km_260511_055922_497700_6a01703a7985a_hsa-mir-197.pdf]

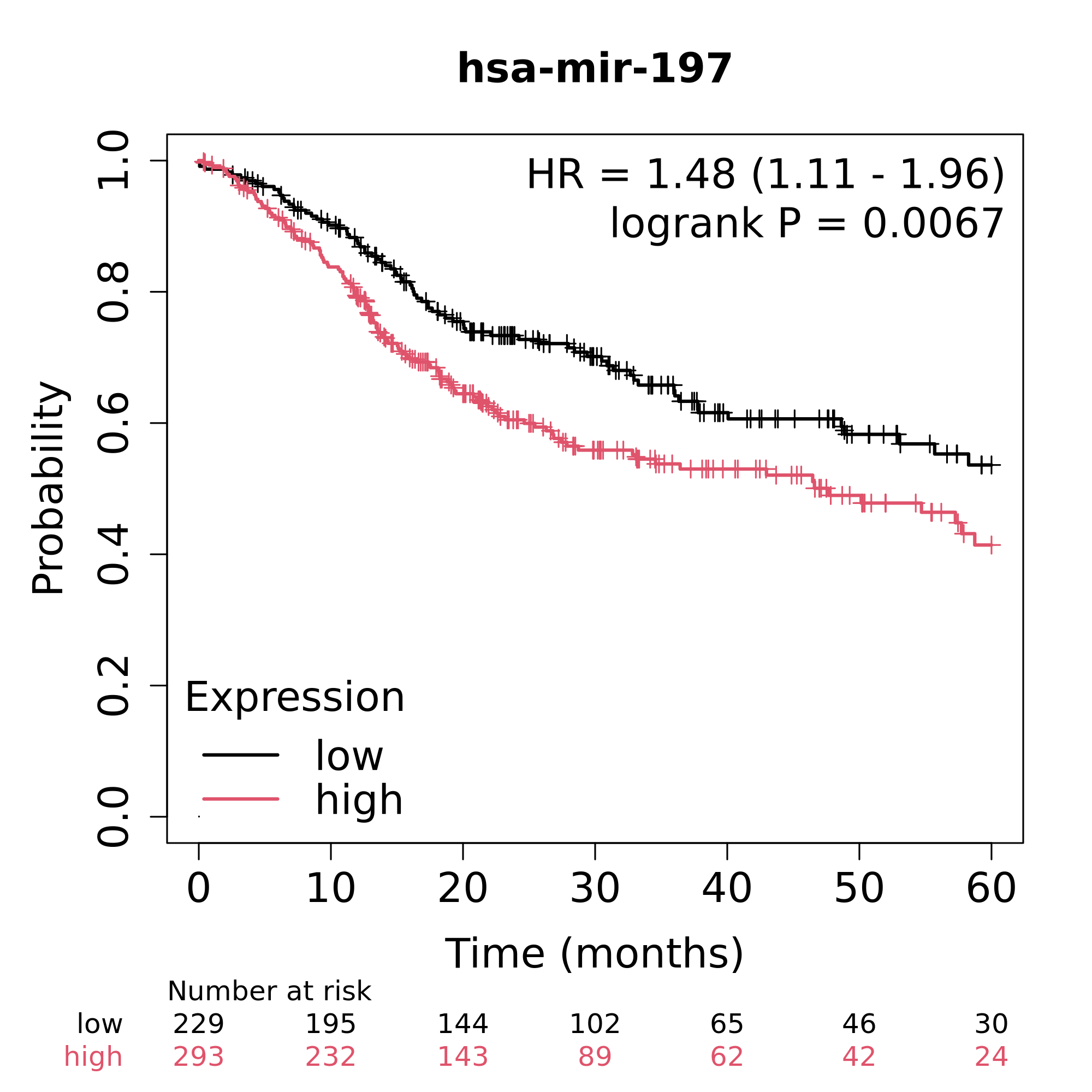

Supplement: Supplementary file 1 [file ijms-27-04909-s001.zip › validation/Set 2 — TCGAKM Plotter database-derived validation/TGCA mir 197 HNSCC/km_260511_055922_497700_6a01703a7985a_hsa-mir-197.png]
